# Efficacy and safety of European Medicines Agency (EMA)‐approved pharmacological, endoscopic, and surgical treatments in different classes of obesity: A network meta‐analysis of randomised controlled trials for the development of the SIO (Società Italiana Obesità) Italian guidelines for the diagnosis and treatment of overweight and obesity

**DOI:** 10.1111/dom.70204

**Published:** 2025-10-20

**Authors:** Rocco Barazzoni, Matteo Monami, Silvio Buscemi, Luca Busetto, Maurizio De Luca, Giuseppe Navarra, Benedetta Ragghianti, Giovanni Antonio Silverii, Amanda Belluzzi, Edoardo Mannucci, Paolo Sbraccia

**Affiliations:** ^1^ Department of Medical, Surgical and Health Sciences University of Trieste Trieste Italy; ^2^ Italian Society of Obesity (SIO) Pisa Italy; ^3^ Diabetology Careggi Hospital and University of Florence Florence Italy; ^4^ Department of Promozione della Salute, Materno‐Infantile, Medicina Interna e Specialistica di Eccellenza (PROMISE) University of Palermo Palermo Italy; ^5^ Department of Medicine University of Padova Padova Italy; ^6^ Rovigo Hospital, ULSS5 Polesana Rovigo Italy; ^7^ Department of Surgical Sciences, Faculty of Medicine University of Messina, G. Martino University Hospital Messina Italy; ^8^ Department of Systems Medicine University of Rome Tor Vergata Rome Italy

**Keywords:** endoscopic bariatric procedures, metabolic bariatric surgery, network meta‐analysis, obesity, obesity management medications

## Abstract

**Aims:**

We aimed at comparing different approved strategies (obesity management medications—OMM, endoscopic bariatric procedures—EBP, and metabolic bariatric surgery—MBS) with lifestyle intervention/placebo/no therapy (LSI/Pbo/NT) for the treatment of different BMI‐based classes of obesity (i.e., overweight—BMI: 25–29.9 kg/m^2^; class I—BMI: 30–34.9 kg/m^2^; class II—BMI: 35–39.9 kg/m^2^; class III—BMI >39.9 kg/m^2^).

**Materials and Methods:**

This systematic review (SR) and network meta‐analysis (NMA) included randomised clinical trials (RCTs) comparing OMM, EBP, and MBS versus either LSI/Pbo/NT or active comparators in individuals with overweight or obesity. A Medline and Embase search was performed up to 31st January 2025 for RCTs on EMA (European Medicines Agency)‐approved weight‐loss interventions in adults with overweight/obesity. The primary endpoint was total body weight loss (TBWL%), analysed at different time points: 26–52, 53–104, 105–156, and ≥156 weeks. Secondary endpoints included all‐cause mortality, quality of life, and serious adverse events (SAE). Weighted mean difference and 95% confidence intervals (WMD, 95% CI) for continuous variables and Mantel–Haenszel odds ratio (MH‐OR, 95% CI) for categorical variables were calculated using random effect models. The study was registered on the PROSPERO website (CRD42024625338).

**Results:**

In trials enroling subjects in class I of obesity, tirzepatide resulted in equal effectiveness to both OAGB and RYGB, and it was significantly superior to all the other comparisons. In trials on class II of obesity, tirzepatide was significantly superior to all the other comparisons and inferior to both OAGB and RYGB. Semaglutide was associated with a higher TBWL% than the other OMMs (with the notable exception of tirzepatide), and it was equally effective to EBP, GCP, and LAGB. In trials enroling patients with a mean BMI >40 kg/m^2^, the procedure with the highest estimated weight loss was BPD. Semaglutide was statistically less effective than SG and gastric bypass, but not inferior to GCP and LAGB. Both RYGB and OAGB were superior to SG.

**Conclusion:**

In patients affected by mild to moderate obesity, newer OMMs (i.e., tirzepatide and semaglutide) appear to be valid alternatives to EBP and MBS. They could be preliminarily chosen as a first‐line option based on similar efficacy and greater safety and tolerability. Higher degrees of obesity could be more effectively treated with MBS, the efficacy of which, with the notable exception of LAGB and GCP, appears superior to other treatments, especially in the long term.

## 1 INTRODUCTION

Obesity has reached epidemic proportions worldwide, representing a major challenge for healthcare systems and society at large. The huge burden of obesity‐associated complications includes metabolic syndrome, type 2 diabetes, cardiometabolic diseases, all major end‐stage organ failures, cancer, disabilities, and psychological‐mental comorbidities, with substantial use of limited healthcare resources.^1^, ^2^ Growing costs of obesity and associated diseases make the implementation of effective therapeutic strategies a largely unmet, but urgent, medical need. In the last three decades, the growing implementation of surgical and endoscopic bariatric procedures has improved treatment effectiveness in terms of weight loss, albeit in a relatively limited number of patients.^3^, ^4^ In more recent years, incretin‐mimetic anti‐obesity medications with unprecedented effectiveness and safety have provided an alternative treatment strategy, with an increasingly overlapping weight loss range compared to bariatric approaches.^5^, ^6^, ^7^, ^8^

Importantly, increasing availability of effective treatment strategies may allow tailoring obesity management not only on disease severity (currently based on BMI categories) and weight‐loss targets, but also on potential treatment or prevention of major comorbidities that may be achieved with medications or surgery.^8^ In this perspective, traditional step‐wise obesity management, postponing pharmacological and surgical treatment until failure to meet weight goals with medical‐nutritional approaches is established, may need to be urgently questioned.^9^ Identification of multimodal pharmacological and/or surgical treatment strategies to be potentially implemented along with nutritional and physical activity‐based interventions with optimised risk–benefit balance becomes therefore a key question for clinical research.

The Italian Obesity Society [Società Italiana dell'Obesità (SIO)] is developing a national guideline for the treatment of obesity, following the Grades of Recommendation, Assessment, Development and Evaluation (GRADE) methodology,^10^ based on a systematic review of available evidence on efficacy and safety of available obesity treatments. The present study reports on the results of a systematic review (SR) followed by a network meta‐analysis (NMA) on randomised clinical trials (RCTs) comparing obesity‐management medications (OMM), endoscopic bariatric procedures (EBS), and metabolic bariatric surgery (MBS) versus either lifestyle interventions (LSI), placebo or no treatment, or other active comparators, in individuals with overweight or obesity. In particular, this NMA is aimed at providing healthcare practitioners and professionals involved in obesity management with a comprehensive picture of the efficacy and safety of available EMA (European Medicines Agency)‐approved treatment options, with the highest quality of evidence as requested by GRADE methodology.

## 2 MATERIALS AND METHODS

**FIGURE 1:**
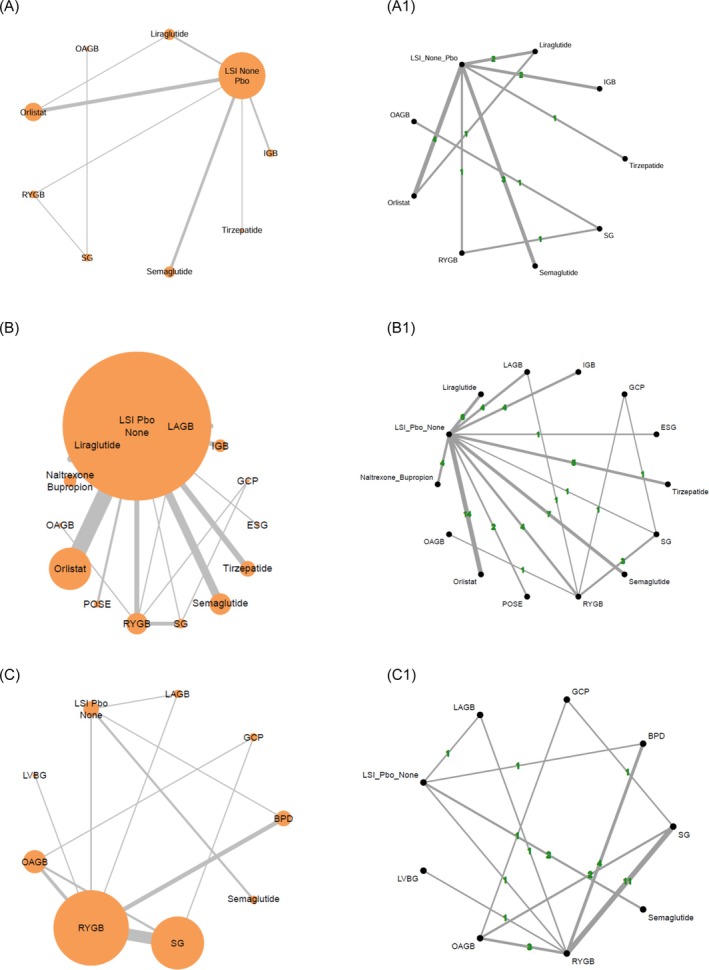
Comparisons between different anti‐obesity strategies on TBWL% at the endpoint (RCTs with BMI at entry: Panel A: 30–34.9; Panel B: 35–39.9; Panel C: >39.9 kg/m^2^). The two panels report the geometric network (A–C) and the number (A1–C1) of comparisons for each class of obesity. The node size represents the number of subjects included, and the edge (line) thickness indicates the number of comparisons assessing the relationship. NB, Naltrexone/Bupropione; POSE, Primary Obesity Surgery Endoluminal; IGB, Intra‐Gastric Balloon; ESG, Endoscopic Sleeve Gastroplasty; LAGB, Laparoscopic Adjustable Gastric Banding; GCP, Greater Curvature Plication Gastric; LVGB, Laparoscopic Vertical Banded Gastroplasty; SG, Sleeve Gastrectomy; OAGB, One‐anastomosis gastric bypass; RYGB, Roux‐en‐Y Gastric Bypass; SADI, Single Anastomosis Duodenal Switch; BPD, Bilio‐Pancreatic Diversion.

The meta‐analysis has been reported following the criteria of the Preferred Reporting Items for Systematic Reviews and Meta‐Analyses (PRISMA) statement^10^, ^11^ (Figure 1S and Table 1S).

### 2.1 Search strategy and selection criteria

The protocol of the present meta‐analysis and network meta‐analysis (NMA) was published on the PROSPERO website (https://www.crd.york.ac.uk/prospero/#recordDetails, registration number: CRD42024625338) and in a previous article. The present analysis included all randomised control trials (RCTs) enroling patients with BMI greater than or equal to 27 kg/m^2^, comparing EMA‐approved OMM, EPB, and MBS versus LSI/Pbo/NT (lifestyle interventions, placebo, or standard of care/no intervention) or comparing two different active treatments. To be included in the analyses, RCTs should have a minimum follow‐up (for MBS)/treatment (for OMM) of 52 weeks, except for EBP, for which a follow‐up/treatment period of 6 months was considered. A Medline, Embase, and Cochrane Central Register of Controlled Trials (CENTRAL) search was performed up to 31st January 2025. Detailed information on the search strategy and keywords used is reported in Table 2S of the Supplementary Materials. Animal studies were excluded, whereas no language or date restriction was imposed.

Duplicate records were removed with EndNote X9 (Clarivate Analytics, Philadelphia, PA, USA). Teams of paired reviewers independently used EndNote X9 to screen titles and abstracts, then full‐text manuscripts, and extracted data on studies fulfilling inclusion and exclusion criteria.

### 2.2 Interventions assessed

*OMM*: orlistat (360 mg), naltrexone plus bupropion (NB, 32/360 mg), liraglutide (3.0 mg), semaglutide (2.4 mg), and tirzepatide (10–15 mg) versus placebo/none or active comparators.

*MBS*: Sleeve Gastrectomy (SG), Roux en Y Gastric Bypass (RYGB), One Anastomosis Gastric Bypass (OAGB), Laparoscopic Adjustable Gastric Banding (LAGB), BilioPancreatic Diversion (BPD), Single Anastomosis Duodenal‐Ileal bypass (SADI), and GCP (Greater Curvature Plication) versus placebo/none or active comparators.

*EBP*: Intragastric Balloons (IB), Primary Obesity Surgery Endoluminal (POSE), and Endoscopic Sleeve Gastroplasty (ESG) versus placebo/none or active comparators.

### 2.3 Data extraction

Information on the baseline characteristics of the samples enroled (age, gender, proportion of patients with T2D, baseline BMI, total body weight loss (TBWL%), waist circumference, body composition, proportion of patients achieving at least 5%, 10%, 15%, 20%, and 25% body weight reduction, remission or improvement/resolution of obesity‐associated medical conditions (OAMC), serious adverse events (SAE), mortality, major adverse cardiovascular events (MACE), fasting plasma glucose (FPG), glycated haemoglobin (HbA1c), lipid profile, estimated glomerular filtration rate (eGFR), creatinine, albuminuria, mental health parameters, and quality of life—QoL) were independently extracted by two authors (B.R., M.M.), and conflicts were resolved by a third investigator (E.M.; Table 3S of Supplementary Materials). Whenever needed, secondary publications and clinicaltrials.gov registry were used to retrieve missing information in the hierarchical order reported above. For each trial, TBWL% was extracted at the last available time point between 26 and 52 weeks, 53 and 104 weeks, 105 and 156 weeks, and after 156 weeks. Two authors performed data extraction independently (B.R., A.B.), and conflicts were resolved by a third investigator (M.M.). Only Intention‐To‐Treat (ITT) analyses were performed.

The risk of bias was assessed using the Cochrane recommended tool to determine the risk of bias in RCTs.^12^ The risk of bias was described and evaluated in seven specific domains: random sequence generation, allocation concealment, blinding of participants and personnel, blinding of outcome assessment, incomplete outcome data, selective reporting, and other biases. The results of these domains were graded as ‘low’ risk of bias, ‘high’ risk of bias, or ‘uncertain’ risk of bias. Two researchers (A.B. and BR) independently assessed the risk of bias in individual studies, with discrepancies resolved by a third researcher (M.M.).

### 2.4 Data analysis

All the analyses have been performed by stratifying included RCTs based on mean baseline BMI: overweight (BMI 27–29.9), Class I (BMI 30–34.9), Class II (BMI 35–39.9), and Class III (BMI above 40 kg/m^2^), and, whenever available, using prespecified subgroup analyses of included RCTs (Figure 1).

The principal endpoint was TBWL% (as change‐from‐baseline parameter); secondary endpoints were waist circumference, body composition, proportion of patients achieving at least 5%, 10%, 15%, 20%, and 25% body weight reduction, remission or improvement/resolution of OAMC, SAE, mortality, MACE, FPG, HbA1c, lipid profile, eGFR, creatinine, albuminuria, mental health parameters, and QoL. The primary endpoint was analysed at different time points: 26–52 (up to 1 year), 53–104 (1–2 years), 105–156 (2–3 years), and ≥156 (≥3 years) weeks. Secondary endpoints (usually reported at the end of the study) were analysed separately in trials with a duration of 26–52 (up to 1 year), 53–104 (1–2 years), 105–156 (2–3 years), and ≥156 (≥3 years) weeks.

### 2.5 Statistical analyses

Mean and 95% confidence intervals (95% CI) for continuous variables and Mantel–Haenszel odds ratio [MH‐OR] for categorical variables were calculated using random effect models. When data were reported as least‐squares mean and standard error, standard deviation (SD) was obtained for each group using the following formula: SD = √(number of patients) * (CI upper limit—CI lower limit)/3.92 and SD = √(number of patients) * SE, respectively (http://handbook-5-1.cochrane.org/chapter_7/7_7_3_2_obtaining_standard_deviations_from_standard_errors_and.htm).

Several prespecified subgroup analyses were performed for the following baseline variables: different types of antiobesity strategies (i.e., surgical and endoscopic procedures, and OMM) and type 2 diabetes mellitus (T2DM; yes: RCT enroling at least 75% of patients with diabetes; no: RCT enroling no more than 25% of patients with T2DM). Traditional meta‐analyses were performed for all the placebo‐ and active‐controlled trial endpoints. Heterogeneity was assessed by using *I* ^2^ statistics. A random‐effects model was applied for all the analyses reported above. Funnel plots were used for endpoints with at least 10 RCTs to assess possible publication biases.

We performed several network meta‐analyses (NMA; frequentist framework)^13^ for all the above outcomes to verify differences across individual anti‐obesity strategies concerning their effects on primary and secondary endpoints. These analyses enable indirect comparisons when direct trials are unavailable, by utilising differences from standard comparators and then combining direct and indirect comparisons to obtain a final effects estimate. The reference category was LSI/Pbo/NT (considered unique). For each outcome, the pooled effect of one intervention versus another was determined by carrying out a random effects NMA. With regard to the primary outcome (TBWL% at the endpoint) across different classes of obesity, a league table was applied to display the mean differences (MDs) with the corresponding 95% confidence intervals (95% CIs).

#### 2.5.1 Assessment network geometry

The graphical representation of the geometry of all networks of interventions was depicted using diagrams that allowed for the representation of whether information comparing each pair of interventions came from direct evidence (i.e., studies comparing two interventions head‐to‐head against one another), indirect evidence (i.e., studies comparing two interventions through a common comparator, called reference category), or both (combination of direct and indirect evidence for estimating the relative effect of pairs of interventions across a network of interventions). All diagrams were composed of nodes (i.e., circles representing each intervention included in the NMA) and links (i.e., lines connecting two nodes). A link between two nodes indicates that there is direct evidence for the comparison. Node size and edge thickness, as well as colours, were used to represent different characteristics of the network, including the number of studies comparing two interventions, the number of participants in each comparison, and the risk of bias. Multi‐arm studies (i.e., primary studies with three or more arms comparing different interventions) were reported for the primary endpoint.

#### 2.5.2 Assessment of transitivity

When direct comparisons (i.e., no head‐to‐head comparisons) are not available between two different interventions (A and B), but each of those interventions has been compared against a common intervention (i.e., A and B have been directly compared to C), the indirect comparison is reliable and unbiased only if the study characteristics (modifiers) of the direct comparisons are not significantly different between the two direct comparisons (i.e., A vs. C, and B vs. C). The distribution of potential effect modifiers across the existing direct comparisons was compared to assess the assumption of transitivity. The following effect modifiers were taken into account: mean age, BMI, and proportion of women, and their effects explored using Network Metaregressions (NMR). We adopted exchangeable models (i.e., coefficient is different for each treatment comparison but all come from a shared distribution), in which the interactions are assumed to be from a common normal distribution with mean and variance to be estimated by the data.

#### 2.5.3 Assessment of heterogeneity

*τ* ^2^ and *τ* values were calculated for each comparison of NMA for the primary endpoint. *τ* ^2^ expresses the between‐study variance, providing a direct measure of heterogeneity at the network level. *τ* is the estimated standard deviation of heterogeneity across studies.

#### 2.5.4 Consistency assessment

The level of statistical agreement between direct and indirect evidence was assessed for the principal outcome to verify that differences between direct and indirect estimates (used to calculate the NMA estimates) were trivial. Inconsistency was tested within each comparison and with the node‐splitting model for all studies (Metainsight v.6.0.0: https://crsu-metainsight.le.ac.uk/MetaInsight/). *H* values were also calculated to test consistency between direct and indirect evidence; an *H* value of less than 3 indicates minimal inconsistency in treatment effects (MetaXL: www.epigear.com).

#### 2.5.5 Sensitivity analyses

Sensitivity analyses were run by focusing only on studies judged to have a low risk of bias and by excluding studies with a high risk of bias.

#### 2.5.6 Risk of bias assessment and evidence credibility

The Grading of Recommendations Assessment, Development and Evaluation (GRADE) system was adopted to assess the risk of bias (i.e., selection, performance, blinding, detection, attrition, reporting, and other biases) for all included RCTs, using the GRADEpro GDT software.

The GRADE system, as extended to NMA, was used to assess the credibility of the evidence. The CINeMA web tool was adopted to evaluate the results of the NMA. Within‐study risk of bias, reporting bias, indirectness, imprecision, heterogeneity, and inconsistency were judged qualitatively. The level of concerns for each treatment effect of NMA was judged as ‘no concerns’, ‘some concerns’, or ‘major concerns’ for each of the 6 domains.

#### 2.5.7 Software programs adopted

NMA was performed using three different software programs: Metainsight v. 6.0.0 (https://crsu-metainsight.le.ac.uk/MetaInsight/), MetaXL (www.epigear.com), and CINeMA (https://cinema.ispm.unibe.ch/#). All other analyses were performed using Review Manager (RevMan), Version 5.3 (Copenhagen: The Nordic Cochrane Centre, The Cochrane Collaboration, 2014).

GRADE methodology was used to assess the quality of the body of retrieved evidence for the principal endpoint, using the GRADEpro GDT software (GRADEpro Guideline Development Tool, McMaster University, 2015.^10^ Available from gradepro.org).

## 3 RESULTS

### 3.1 Retrieved trials

The trial flow summary is reported in Figure 1S of the Supplementary Materials. The search of CENTRAL, Medline, and Embase databases allowed the identification of 129 trials fulfilling all inclusion criteria: 52, 13, and 64 trials on MBS, EP, and OMMs were compared with either LSI/Pbo/NT or other active anti‐obesity strategies. Some trials reported multiple comparisons.^14^, ^15^, ^16^, ^17^, ^18^, ^19^ Therefore, the number of available comparisons was 140. The overall number of patients enroled was 60,044, 2217, and 5991 in trials with OMM, EP, and MBS, respectively (Table 5S of Supplementary Materials).

**FIGURE 2:**
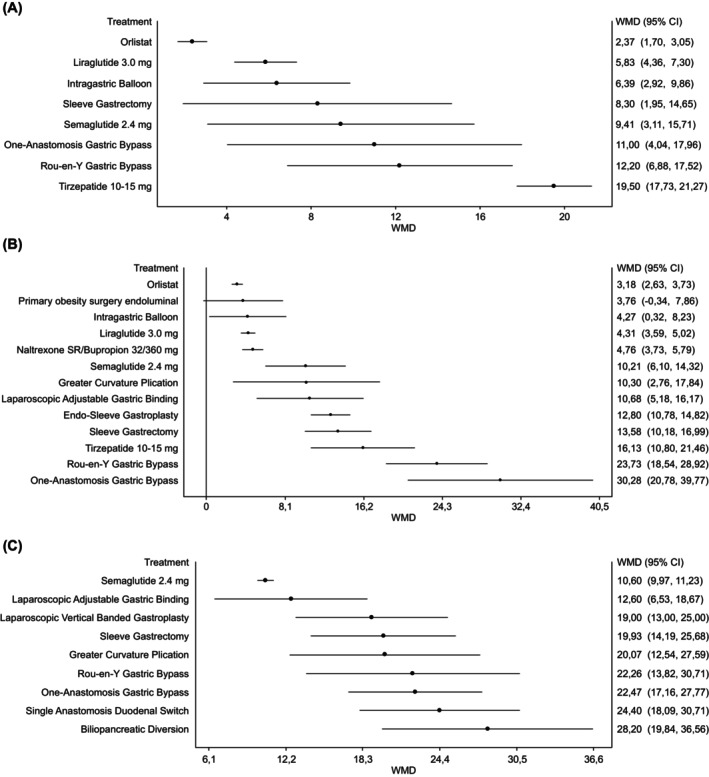
Effects of different anti‐obesity strategies on TBWL% at the endpoint (RCTs with BMI at entry: Panel A: 30–34.9; Panel B: 35–39.9; Panel C: >39.9 kg/m^2^). NB, Naltrexone/Bupropione; POSE, Primary Obesity Surgery Endoluminal; IGB, Intra‐Gastric Balloon; ESG, Endoscopic Sleeve Gastroplasty; LAGB, Laparoscopic Adjustable Gastric Banding; GCP, Greater Curvature Plication Gastric; LVGB, Laparoscopic Vertical Banded Gastroplasty; SG, Sleeve Gastrectomy; OAGB, One‐anastomosis gastric bypass; RYGB, Roux‐en‐Y Gastric Bypass; SADI, Single Anastomosis Duodenal Switch; BPD, Bilio‐Pancreatic Diversion.

The main characteristics of the included trials, divided by mean BMI at entry (class I, II, and III of obesity), are reported in Table 5S of Supplementary Materials. The quality of studies was heterogeneous (Figure 2S of Supplementary Materials). All trials on surgical and endoscopic procedures, except seven (11%),^20^, ^21^, ^22^, ^23^, ^24^, ^25^, ^26^ were open‐label. In many trials, the attrition rate and/or the description of allocation and blinding of assessors were inadequate (Figures 2S and 3S of Supplementary Materials). Trials on OMM were more frequently double‐blinded (66%), with fewer trials with inadequate attrition and/or description of allocation or blinding of assessors (29.3%).

### 3.2 Data derived from pre‐planned subgroup analyses across different BMI classes

#### 3.2.1 Weight loss

Only three trials reported separately the results on weight loss in different categories of BMI at study entry. One study with liraglutide^27^ provided data for patients with overweight (BMI 27–29.9 kg/m^2^) and with different degrees of obesity (class I, II, and III). All categories of patients reported a significantly higher placebo‐subtracted TBWL%, ranging from 3.7% to 5.2%. The other two studies^28^, ^29^ reported a significantly greater TBWL% at endpoint with semaglutide than with placebo in all BMI classes. The placebo‐subtracted effect of semaglutide was 12.40 [7.13, 17.67], 15.60 [12.65, 18.55], 17.00 [13.64, 20.36], and 13.90 [10.78, 17.02]% for overweight, class I, II, and III of obesity, respectively (all *p* < 0.001; test for subgroup differences: *p* = 0.40).^28^ Similar figures were obtained for the other study, with a TBWL% ranging from 9.6% to 11.3% (these data are reported in a subsequent publication^30^).

#### 3.2.2 Major cardiovascular events (MACE)

For patients with overweight (BMI 27–29.9 kg/m^2^), only one study with semaglutide reported data on incident MACE, showing that the interventional drug was associated with a significantly lower risk.^31^

For patients with BMI at study entry between 30 and 35 kg/m^2^, subgroup analyses were available for three trials with semaglutide,^31^, ^32^, ^33^ three with liraglutide,^14^, ^34^, ^35^ and one with tirzepatide.^36^ A statistically significant reduction of incident MACE was observed only for semaglutide (Figure 11S; Panel A).

For patients with BMI 35–39.9 kg/m^2^ and >39.9 kg/m^2^, only one study with semaglutide reported data on incident MACE, showing no between‐group differences.^31^

### 3.3 Data derived from separate analyses of trials based on mean BMI at enrolment

Comparisons across BMI categories have been performed mainly through separate analyses of trials based on mean BMI at enrolment.

#### 3.3.1 Weight loss

##### Trials with mean BMI at entry ranging from 27 to 29.9 kg/m^2^

Only one study^37^ comparing RYGB with LSI and performed in an Asian population with type 2 diabetes reported a mean BMI at entry <30 kg/m^2^. The TBWL% at the end of the trial was significantly superior in the intervention arm at any assessed time points (i.e., WMD: 15.50 [12.53, 18.47], 12.50 [9.53, 15.47], 12.50 [9.53, 15.47], and 11.20 [8.23, 14.17] %, all *p* < 0.001, at 52, 104, 156, and >156 weeks, respectively). The between‐group difference of BMI at the endpoint was −5.20 [−7.12, −3.28] kg/m^2^ (*p* < 0.001).

##### Trials with a mean BMI at entry ranging from 30 to 34.9 kg/m^2^

We retrieved 22 trials^14^, ^31^, ^32^, ^33^, ^34^, ^35^, ^38^, ^39^, ^40^, ^41^, ^42^, ^43^, ^44^, ^45^, ^46^, ^47^, ^48^, ^49^, ^50^, ^51^, ^52^, ^53^ (Table 6S of Supplementary Materials) with a mean baseline BMI between 30 and 34.9 kg/m^2^. Figure 2 (Panel A) and the league table of all pairwise comparisons (Table 7S) reported results for TBWL% at the endpoint. Tirzepatide resulted in equal effectiveness to OAGB and RYGB, and it was significantly superior to all the other comparisons. Semaglutide was superior to liraglutide, orlistat, and IGB, and not inferior to the other comparisons, except tirzepatide. Results on weight loss at different time points, summarised in Table 1 and Figure 4S, were similar to those at the endpoint; notably, results after 2 or more years were available only for semaglutide and RYGB.

**TABLE 1:** Synthesis of results (lifestyle intervention/placebo/no therapy‐subtracted effects, if not otherwise specified) for each critical outcome at the endpoint (if not otherwise specified) reported for each anti‐obesity intervention in trials with mean BMI at entry ranging from 30 to 34.9 kg/m^2^.

	Parameter	Orlistat	Liraglutide	Semaglutide	Tirzepatide	IGB	OAGB	RYGB	SG	LAGB
Body weight	TBWL (%)									
	No. of comparisons (all)	*n* = 6	*n* = 4	*n* = 3	*n* = 1	*n* = 4	*n* = 1	*n* = 4	*n* = 2	*n* = 1
	At 26–52 weeks	**2.4**	**5.8**	**9.0**	**19.5**	**6.4**	**14.3**	**16.7**	**11.9**	*NR*
	At 53–104 weeks	NA	NA	**11.6**	NA	NA	NA	**12.2**	NA	*NR*
	At 105–156 weeks	NA	NA	**8.7**	NA	NA	NA	**12.8**	NA	*NR*
	At 157–260 weeks	NA	NA	NA	NA	NA	NA	**11.4**	NA	*NR*
	At the endpoint	**2.4**	**5.8**	**9.4**	**19.5**	**6.4**	**11.0**	**12.2**	**8.3**	*NR*
	BMI (kg/m^2^)	**−1.1**	**−1.2**	**−3.5**	**−5.2**	**−1.7**	NR	**−5.0**	NR	**−7.3**
	Waist circumference (cm)	**−2.8**	**−3.7**	**−9.6**	**−12.1**	NR	NR	**−16.3**	NR	NR
Glucometabolic control	HbA1c (mmol/mol)	**−8.8**	NA	−3.3	0.0	NA	**−23.5**	**−14.5**	**−12.5**	NR
	FPG (WMD, mg/dL)	0.7	**−7.2**	−7.4	−0.8	−1.0	**−43.7**	**−30.5**	**−28.7**	NR
	Total cholesterol (WMD, mg/dL)	**−23.6**	NA	−4.8	−1.1	−3.0	**−76.1**	−37.0	−33.1	NR
	HDL‐cholesterol (WMD, mg/dL)	**1.4**	NA	−0.1	0.4	0.0	**9.6**	**11.1**	**10.7**	NR
	Triglycerides (WMD, mg/dL)	−**16.4**	0.0	**−19.4**	4.2	NA	**−98.3**	−71.9	−62.9	NR
	SBP (WMD, mmHg)	−2.9	NA	−1.3	NA	0.0	−1.5	**−3.5**	2.5	NR
	DBP (WMD, mmHg)	−2.1	NA	**−1.2**	NA	0.0	1.5	−0.5	3.5	NR
Obesity‐associated medical conditions	MACE (OR)^	NA	NE	**0.76**	7.1	NA	NA	NA	NA	NA
	Diabetes remission (OR)^	NA	NA	**0.28**	NA	NA	NA	NA	NA	NA
	Incident diabetes (OR)^	NA	NA	**0.25**	NA	NA	NA	NA	NA	NA
	Hospitalisation HF (OR)^	NA	NA	0.81	NA	NA	NA	NA	NA	NA
	OSAS remission^a^	NA	NA	NA	NA	NA	NA	NA	NA	NA
	Liver fibrosis reduction^b^	NA	NA	NA	NA	NA	NA	NA	NA	NA
	MASH remission^c^	NA	NA	NA	NA	NA	NA	NA	NA	NA
	Hyperten. remission (OR)	NA	NA	NR	NA	NA	NA	NA	NA	NA
	Dyslipid. remission (OR)	NA	NA	NR	NA	NA	NA	NA	NA	NA
Safety	SAE (OR)^	NE	0.89	0.86	1.00	0.15	NA	NA	NA	NA
	Surgical SAE (OR)^	–	–	–	–	2.8	**NA** ^d^	**28.2**	**NA** ^d^	NA
	All‐cause mortality (OR)	0.96	0.99	**0.81**	1.00	0.71	3.1	NA	NA	NA

*Note*: Bold character: *p* < 0.050. NMAs have been performed only for outcomes with at least 10 RCTs; for all the other outcomes (^), we performed traditional meta‐analyses (see Supplementary Materials, Figures 4S and 12S).

Abbreviations: IGB, IntraGastric Balloon; LAGB, Laparoscopic Adjustable Gastric Banding; NA, not available; NE, not estimable (zero cases in the interventional and placebo arms); OAGB, One‐Anastomosis Gastric By‐pass; OR, odds ratio; RYGB, Rou‐en Y Gastric By‐pass; SG, Sleeve GAstrectomy; WMD, weighted mean difference.

^a^ Defined as AHI <5 or AHI of 5–14.

^b^ Improvement (decrease) of at least one fibrosis stage without worsening of MASH.

^c^ MASH resolution without worsening of fibrosis.

Heterogeneity (*τ* ^2^ values) was assessed for all the available comparisons, showing some concerns for IGB, OAGB, liraglutide, and orlistat versus the reference category (Figure 5S of Supplementary Materials). Table 8S and Figure 6S of the Supplementary Materials report data on inconsistency for each comparison, detecting no major concerns. Visual analysis of the funnel plot for trials either versus placebo or standard of care (‘none’) did not suggest any relevant publication bias for TBWL% at the endpoint (Figure 7S of Supplementary Materials).

A reduction of BMI at endpoint greater than 5 kg/m^2^ (Figure 8S) and a reduction of waist circumference (Figure 9S) greater than 10 cm were observed only for tirzepatide and RYGB (Table 1).

##### Trials with a mean BMI at entry ranging from 35 to 39.9 kg/m^2^

Fifty‐seven trials^15^, ^18^, ^19^, ^24^, ^26^, ^53^, ^54^, ^55^, ^56^, ^57^, ^58^, ^59^, ^60^, ^61^, ^62^, ^63^, ^64^, ^65^, ^66^, ^67^, ^68^, ^69^, ^70^, ^71^, ^72^, ^73^, ^74^, ^75^, ^76^, ^77^, ^78^, ^79^, ^80^, ^81^, ^82^, ^83^, ^84^, ^85^, ^86^, ^87^, ^88^, ^89^, ^90^, ^91^, ^92^, ^93^, ^94^, ^95^, ^96^, ^97^, ^98^, ^99^, ^100^ (Table 9S of Supplementary Materials) with mean BMI at enrolment between 35 and 39.9 kg/m^2^ were available for analysis. Liraglutide was not superior to orlistat and NB, and equally effective as EBP (except for ESG). Semaglutide was associated with a higher TBWL% than the other OMMs (with the notable exception of tirzepatide) and was equally effective to EBP, GCP, and LAGB, but gastric bypass. Tirzepatide was significantly superior to all the other comparisons, except for GCP and ESG (not inferior), and it was associated with lower TBWL% than both OAGB and RYGB (Figure 2, panel B and Table 10S).

Results at different time points (Figure 10S) were consistent with those at endpoint, and effects on BMI and waist circumference (Figure 11S) were consistent with those on TBWL% (Table 2). Notably, data on longer‐term (>2 years) weight loss were available only for RYGB and LABG (Table 2). Heterogeneity (*τ* ^2^ values) was assessed for all the available comparisons, showing some concerns NB, liraglutide, orlistat, and POSE versus the reference category (Figure 12S of Supplementary Materials). Table 11S and Figure 13S of the Supplementary Materials report data on inconsistency for each comparison detecting no major concerns. Visual analysis of the funnel plot for trials either versus placebo or standard of care (‘none’) did not suggest any relevant publication bias for TBWL% at the endpoint (Figure 14S of Supplementary Materials).

**TABLE 2:** Synthesis of results (Lifestyle intervention/Placebo/No therapy‐subtracted effects, if not otherwise specified) for each critical outcome at the endpoint (if not otherwise specified) reported for each anti‐obesity intervention in trials with mean BMI at entry ranging from 35 to 39.9 kg/m^2^.

	Parameter	Orlist.	NB	Liragl.	Semagl.	Tirzep.	ESG	IGB	POSE	OAGB	RYGB	SG	LAGB	GCP
Body weight	TBWL (%)													
	No. of comparisons (all)	*n* = 16	*n* = 5	*n* = 6	*n* = 7	*n* = 5	*n* = 1	*n* = 4	*n* = 2	*n* = 1	*n* = 11	*n* = 5	*n* = 5	*n* = 2
	At 26–52 weeks	**3.8**	**4.8**	**5.0**	**11.8**	**15.0**	**12.8**	**4.3**	3.8	**25.0**	**20.1**	**11.8**	**8.8**	NA
	At 53–104 weeks	**3.2**	**4.8**	**5.1**	**11.7**	**14.4**	NA	NA	NA	NA	**20.6**	**20.6**	**10.2**	NA
	At 105–156 weeks^a^	**3.0**	**4.2**	NA	NA	NA	NA	NA	NA	NA	**24.1**	NA	**7.3**	NA
	>157 weeks^a^	**3.0**	NA	NA	NA	NA	NA	NA	NA	NA	**19.5**	NA	**6.8**	NA
	At the endpoint	**3.2**	4.8	**4.3**	**10.2**	**16.1**	**12.8**	**4.3**	3.8	**30.3**	**23.7**	**13.6**	**10.7**	**10.3**
	BMI (kg/m^2^)	**−1.0**	−0.9	**−1.6**	**−3.6**	**−5.9**	**−2.5**	**−2.5**	−1.6	NR	**−6.9**	**−4.2**	**−4.1**	−1.1
	Waist circumference (cm)	**−2.0**	−1.4	**−3.6**	**−7.8**	**−10.9**	NA	NA	NA	NA	**−17.5**	**−15.8**	−5.4	NA
Glucometabolic control	HbA1c (mmol/mol)	**−2.6**	**−5.5**	**−1.8**	−**3.7**	**−16.9**	−8.0	0.0	NA	**−7.0**	**−8.0**	−9.0	**−8.9**	5.5
	Diabetes only	**−4.1**	NA	−4.4	**−15.1**	**−16.9**	NA	NA	NA	NA	**−10.1**	−9.6	**−9.7**	−1.7
	FPG (WMD, mg/dL)	−2.0	−1.3	−8.6	−8.0	**−36.0**	**−11.3**	**−5.0**	0.4	NA	**−18.8**	**−17.8**	−6.4	NA
	Diabetes only	−4.1	**−12.0**	−29.5	**−37.8**	**−36.0**	NA	NA	NA	NA	**−18.8**	**−17.8**	−6.4	NA
	Total cholesterol (mg/dL)	−12.6	NA	**−7.7**	**−7.4**	−4.2	7.7	−6.0	NA	NA	−10.0	−9.0	−6.3	NA
	HDL‐cholesterol (mg/dL)	−0.2	**3.5**	**2.6**	0.2	**6.6**	**5.9**	0.0	NA	NA	**9.5**	4.6	2.2	NA
	Triglycerides (mg/dL)	**−13.8**	−12.8	**−11.4**	**−16.8**	**−29.1**	−13.3	−9.0	NA	NA	**−29.9**	**−15.1**	−17.0	NA
	SBP (WMD, mmHg)	**−1.5**	**1.5**	**−3.2**	**−3.5**	**−6.3**	**−7.1**	**−5.0**	NA	NA	**−5.9**	−1.9	−1.1	NA
	DBP (WMD, mmHg)	**−1.2**	0.4	−0.8	**−2.1**	NA	−4.0	−1.0	NA	NA	−2.4	−1.2	−0.1	NA
Obesity‐associated medical conditions	MACE (OR)^a^	NE	0.89	0.66	0.91	0.79	NA	NA	NA	NA	NA	NE	0.66	NA
	Diabetes remission (OR)	NA	**2.33**	**6.76**	**12.26**	**27.98**	NA	NA	NA	9.05	**18.27**	**13.82**	**7.80**	**6.87**
	Incident diabetes (OR)^a^	**0.61**	NA	**0.27**	0.14	NA	NA	NA	NA	NA	NA	NA	0.25	NA
	Hospitalisation HF (OR)^a^	NA	NA	NE	**0.23**	**0.45**	NA	NA	NA	NA	NA	NA	NA	NA
	OSAS remission^b^ (OR)^a^	NA	NA	NA	NA	**4.19**	NA	NA	NA	NA	NA	NA	NA	NA
	Liver fibrosis^a^ (OR)^c^	NA	NA	NA	0.36	**2.48**	NA	NA	NA	NA	NA	NA	NA	NA
	MASH remission^a^ (OR)	NA	NA	NA	1.96	**11.83**	NA	NA	NA	NA	NA	NA	NA	NA
	Hypert. remission (OR)^a^	NA	NA	**0.01**	**0.05**	NA	NA	NA	NA	NA	NA	NA	0.94	NA
	Dyslip. remission (OR)^a^	NA	NA	1.20	0.66	NA	NA	NA	NA	1.89	**3.54**	1.85	1.20	0.76
Safety	SAE (OR)	1.13	**1.21**	1.19	0.86	0.89	**4.89**	1.44	3.12	**12.88**	**3.90**	**3.46**	**3.16**	3.81
	Surgical SAE (OR)^a^	–	–	–	–	–	**42.18**	**22.34**	12.18	NA^d^	**10.77**	NE^d^	13.58	NA^d^
	All‐cause mortality (OR)	1.30	0.98	0.67	0.86	0.58	1.43	0.63	0.40	0.51	0.47	0.55	0.64	0.43

*Note*: Bold character: *p* < 0.050. NMAs have been performed only for outcomes with at least 10 RCTs. For all the other outcomes, we performed traditional meta‐analyses.

Abbreviations: ESG, EndoSleeve Gastroplasty; GCP, Great Curvature Plication; IGB, IntraGastric Balloon; LAGB, Laparoscopic Adjustable Gastric Banding; Liragl, Liraglutide; NA, not available; NB, Naltrexone/Bupropione; NE, not estimable (zero cases in the interventional and placebo arms); OAGB, One‐Anastomosis Gastric By‐pass; OR, odds ratio; Orlist, Orlistat; POSE, Primary Obesity Surgery Endoluminal; RYGB, Rou‐en Y Gastric By‐pass; Semagl, Semaglutide; SG, Sleeve GAstrectomy; Tirzep, Tirzepatide; WMD, weighted mean difference.

^a^ Data obtained from traditional meta‐analyses versus LSI/Pbo/NT (Lifestyle interventions, Placebo, or No therapy/Standard of care) or comparing different active treatments (see Figures 13S and 20S).

^b^ Defined as AHI <5 or AHI of 5–14.

^c^ Liver fibrosis reduction: improvement (decrease) of at least one fibrosis stage without worsening of MASH; MASH resolution: steatohepatitis resolution without worsening of fibrosis.

^d^ Information on OAGB, GCP safety derived from head‐to‐head comparisons with RYGB, resulting in a non‐significant increased risk of surgical SAE (Figure 18S).

##### Trials with mean BMI at entry >39.9 kg/m^2^

Trials enroling patients with a mean BMI >40 kg/m^2^ (*N* = 47,^21^, ^23^, ^25^, ^101^, ^102^, ^103^, ^104^, ^105^, ^106^, ^107^, ^108^, ^109^, ^110^, ^111^, ^112^, ^113^, ^114^, ^115^, ^116^, ^117^, ^118^, ^119^, ^120^, ^121^, ^122^, ^123^, ^124^, ^125^, ^126^, ^127^, ^128^, ^129^, ^130^, ^131^, ^132^, ^133^, ^134^, ^135^, ^136^, ^137^, ^138^, ^139^, ^140^, ^141^, ^142^, ^143^, ^144^, ^145^ Table 12S of Supplementary Materials) were all performed on surgical procedures, with the only exception of two trials with semaglutide.^101^, ^103^ The procedure with the highest estimated weight loss was BPD (for which no trial on patients with mean BMI <40 kg/m^2^ was available). All the other surgical procedures produced a weight loss greater than 15%, with the only exception of LABG. Semaglutide was statistically less effective than SG and gastric bypass, but not inferior, from a statistical point of view, to LVBG, GCP, and LAGB. Among different types of MBS, BPD was associated with a higher TBWL% than all the other interventions. RYGB and OAGB (equally effective with each other) were superior to SG. LAGB and GCP were associated on average with a lower TBWL% (<20%; Figure 2, Panel C and Table 13S).

Results on %TBWL at different time points (Figure 15S) were similar to those at endpoint. However, the efficacy of LAGB appeared to decrease over time, whereas this phenomenon was not observed with other surgical procedures (Table 3). Results on BMI and (when available) on waist circumference (Figure 16S) were consistent with those on TBWL (Table 3). No heterogeneity (*τ* ^2^ values) was detected for any of the available comparisons (Figure 17S of Supplementary Materials). Table 14S and Figure 18S of Supplementary Materials report data on inconsistency for each comparison detecting possible concerns only for BPD versus LSI/Pbo/NT. No funnel plot has been performed due to the scarce number (*n* = 4) of trials either versus placebo or standard of care (‘none’).

**TABLE 3:** Synthesis of results (Lifestyle intervention/Placebo/No therapy‐subtracted effects, if not otherwise specified) for each critical outcome at the endpoint (if not otherwise specified) reported for each anti‐obesity intervention in trials with mean BMI at entry >39.9 kg/m^2^.

	Parameter	Semagl.	LVGB	SG	GCP	OAGB	BPD	LAGB	RYGB	SADI	IGB
Body weight	TBWL (%)										
	No. of comparisons (all)	*n* = 2	*n* = 10	*n* = 22	*n* = 3	*n* = 10	*n* = 6	*n* = 8	*n* = 32	*n* = 1	*n* = 2
	At 26–52 weeks	**10.4**	**19.0**	**19.5**	**22.7**	**25.1**	**27.0**	**20.8**	**28.8**	**24.4**	NA
	At 53–104 weeks	**10.5**	**17.9**	**18.4**	**14.4**	**20.2**	**25.2**	**21.4**	**24.6**	NA	NA
	At 105–156 weeks	NA	**18.2**	**18.1**	NA	**21.0**	**26.4**	**12.7**	**20.4**	NA	NA
	At 157–260 weeks	NA	**19.0**	**20.3**	NA	**22.4**	**28.2**	**12.6**	**22.3**	NA	NA
	At 261–520 weeks	NA	NA	NA	NA	**22.4**	**27.1**	**12.6**	**22.1**	NA	NA
	At the endpoint	**10.6**	**19.0**	**19.9**	**20.1**	**22.5**	**28.2**	**12.6**	**22.3**	**24.4**	NA
	BMI (kg/m^2^)	**−4.6**	**−9.6**	**−10.0**	**−9.2**	**−12.3**	**−11.9**	**−8.1**	**−10.3**	**−12.2**	**−3.3**
	Waist circumference (cm)	**−8.0**	NA	−4.3	NA	NA	**−17.8**	−3.6	**−9.8**	NA	NA
Glucometabolic control	HbA1c (mmol/mol)	**−5.0**	NA	−2.6	NA	**−7.0**	**−9.9**	NA	**−7.1**	NA	NA
	Diabetes only	NA	NA	**−4.9**	NA	**−7.0**	**−10.0**	NA	**−7.0**	NA	NA
	FPG (WMD, mg/dL)	**−13.5**	**−36.4**	**−16.0**	NA	**−26.5**	**−39.4**	**−7.3**	**−30.0**	NA	NA
	Diabetes only	NA	NA	−12.2	NA	**−26.5**	**−38.2**	NA	**−29.1**	NA	NA
	Total cholesterol (WMD, mg/dL)	NA	10.0	9.1	NA	−0.4	**−62.1**	−7.0	**−18.1**	NA	NA
	HDL‐cholesterol (WMD, mg/dL)	NA	NA	**12.3**	NA	NA	**6.7**	**4.0**	**14.5**	NA	NA
	Triglycerides (WMD, mg/dL)	NA	**−34.0**	**−43.6**	NA	**−54.1**	**−54.0**	**−57.0**	**−70.6**	NA	NA
	SBP (WMD, mmHg)	**−4.7**	3.0	−4.3	NA	NA	**−6.0**	**−6.0**	**−8.8**	NA	NA
	DBP (WMD, mmHg)	−1.0	0.8	0.0	NA	NA	**−3.5**	−1.0	**−5.6**	NA	NA
Obesity‐associated medical conditions	MACE (OR)	NE	NA	NA	NA	NA	NA	NA	NA	NA	NA
	Diabetes remission (OR)	NA	NA	NA	NA	NA	NA	NA	NA	NA	NA
	Incident diabetes (OR)^a^	0.24	NA	NA	NA	NA	NA	NA	NA	NA	NA
	Hospitalisation HF (OR)	NA	NA	NA	NA	NA	NA	NA	NA	NA	NA
	Hyperten. remission (OR)	NA	NA	NA	NA	NA	NA	NA	NA	NA	NA
	OSAS remission (OR)^b^	NA	NA	NA	NA	NA	NA	NA	NA	NA	NA
	Dyslipid. remission (OR)	NA	NA	NA	NA	NA	NA	NA	NA	NA	NA
Safety	SAE (OR)	1.16	NE	0.46	0.24	0.29	**6.31**	0.55	1.80	**19.9**	1.04
	Surgical SAE (OR)	–	**10.2**	5.2	14.2	15.9	**17.1**	2.17	**8.8**	**21.0**	1.04
	All‐cause mortality (OR)	0.50	0.32	0.19	0.10	0.21	0.15	0.79	0.23	0.32	NE

*Note*: Bold character: *p* < 0.050. NMAs have been performed only for outcomes with at least 10 RCTs. For all the other outcomes, we performed traditional meta‐analyses.

Abbreviations: NA, not available; NE, not estimable (zero cases in the interventional and placebo arms); OR, odds ratio; WMD, weighted mean difference.

^a^ Data obtained from traditional meta‐analyses versus LSI/Pbo/NT or comparing different active treatments (see Figures 21S and 27S).

^b^ Defined as AHI <5 or AHI of 5–14.

##### Sensitivity analyses

Several NMRs have been performed to explore the putative interaction of several covariates on the relative treatment effects on TBWL% at endpoint for all obesity classes. Mean age, BMI, and proportion of women at baseline have been tested (Figures 19S–21S of Supplementary Materials), finding no interactions for any of the above‐mentioned covariates across classes of obesity. Trials' characteristics did not differ across different classes of obesity (mean baseline age 48, 48, and 44 years and proportion of women 68, 69, and 68% in class I, II, and III of obesity, respectively). After excluding low‐quality trials, on average MBS reported worse results in terms of efficacy (TBWL%), as reported in Figure 22S of Supplementary Materials.

#### 3.3.2 Metabolic parameters and blood pressure

*HbA1c e FPG*. No specific data were available from trials with a mean BMI at enrolment below 30 kg/m^2^. The number of trials reporting data on glucose metabolism enroling patients with a mean BMI between 30 and 35 was limited (12 and 11 for HbA1c and fasting glucose, respectively), with no available information for several treatments, including EBP, liraglutide, and NB; in addition, the majority of available trials enroled selectively patients with diabetes,^38^, ^41^, ^43^, ^47^, ^48^, ^49^, ^146^, ^147^ with limited data on subjects without diabetes.^31^, ^33^, ^40^, ^148^ Most therapies were associated with a significant reduction of both HbA1c and glucose, whereas the effects of semaglutide and tirzepatide did not reach statistical significance (Figures 23S and 24S, panel B). A greater amount of data was available from trials enroling patients with a mean BMI between 35 and 40 kg/m^2^ (35 and 36 RCTs for HbA1c and FPG, respectively), showing significant improvements for all treatments except SG, GCP, and IGB (Figure 25S). Finally, 13^25^, ^101^, ^102^, ^108^, ^117^, ^119^, ^125^, ^131^, ^132^, ^134^, ^144^, ^145^, ^149^ and 16^25^, ^64^, ^102^, ^108^, ^112^, ^119^, ^123^, ^126^, ^130^, ^131^, ^132^, ^137^, ^144^, ^145^, ^150^ trials enroling patients with a mean BMI >39.9 kg/m^2^ reported data on HbA1c and FPG, respectively; all trials were performed on surgical procedures, with the exception of 2 with semaglutide.^101^, ^103^ All tested treatments reduced HbA1c in patients with diabetes, and most treatments also produced significant reductions of HbA1c when including also subjects without diabetes (Figure 26S). Results on FPG were consistent with those on HbA1c (Figure 26S).

*Lipid profile*. No data on lipid profile were available for BMI <30 kg/m^2^. Of the trials with mean BMI at enrolment between 30 and 34.9 kg/m^2^, 12,^31^, ^32^, ^40^, ^41^, ^42^, ^43^, ^47^, ^48^, ^49^, ^147^, ^151^, ^152^ 12^31^, ^32^, ^40^, ^41^, ^42^, ^43^, ^47^, ^48^, ^49^, ^147^, ^151^, ^152^ and 11^31^, ^32^, ^34^, ^40^, ^41^, ^42^, ^43^, ^45^, ^48^, ^49^, ^152^ reported data on total cholesterol, HDL cholesterol, and triglyceride at endpoint, respectively. MBS and orlistat were associated with a significant reduction of total cholesterol and an increase of HDL cholesterol, whereas orlistat, semaglutide, and OAGB significantly reduced triglyceride levels (Figure 27S). Information on lipid profile was available for 30 trials enroling patients with a mean BMI between 35 and 39.9, showing a significant reduction of total cholesterol with orlistat, liraglutide, and semaglutide, a significant increase of HDL cholesterol with liraglutide, NB, ESG, tirzepatide, and RYGB, and a significant reduction of triglyceride with RYGB, SG, tirzepatide, orlistat, and semaglutide (Figure 28S). In trials enroling patients with a mean BMI >40 kg/m^2^ (*N* = 10 trials^25^, ^64^, ^102^, ^107^, ^112^, ^119^, ^123^, ^126^, ^130^, ^145^), with 12 comparisons, only BPD and RYGB were associated with a significant reduction of total cholesterol, whereas a significant increase in HDL cholesterol was observed for LAGB, BPD, SG, and RYGB, and triglycerides were significantly reduced by all treatments reporting this endpoint (i.e., BPD, LAGB, OAGB, RYGB, SG, and LVGB; Figure 29S).

*Blood pressure*. No trial enroling patients with a mean BMI below 30 kg/m^2^ and reporting the effects of treatment on blood pressure was available. Of the trials enroling patients with a mean BMI between 30 and 34.9 kg/m^2^, 12^31^, ^33^, ^34^, ^36^, ^41^, ^43^, ^45^, ^47^, ^147^, ^151^, ^152^, ^153^ and 12^15^, ^31^, ^32^, ^33^, ^34^, ^36^, ^42^, ^43^, ^45^, ^47^, ^147^, ^152^ trials reported data on systolic (SBP) and diastolic blood pressure (DBP), respectively, showing significant effects for RYGB and semaglutide (data not shown). In trials with mean BMI at enrolment 35–39.9 kg/m^2^ (*N* = 37), all treatments were associated with a reduction of systolic blood pressure, with the notable exceptions of LAGB and SG, which did not show significant effects, and of NB, showing higher blood pressure values at the end of the trial; diastolic blood pressure was significantly reduced only by ESG, semaglutide, and orlistat (Figure 30S). In trials enroling patients with mean BMI >39.9 kg/m^2^ (*N* = 8 trials^25^, ^64^, ^101^, ^102^, ^103^, ^145^, ^149^, ^154^ with 10 comparisons), BPD and RYGB effectively reduced both systolic and diastolic blood pressure, whereas LAGB and semaglutide were associated with lower values of systolic, but not diastolic, blood pressure (Figure 31S).

#### 3.3.3 Obesity‐associated medical conditions

*MACE*. No trial enroling patients with a mean BMI below 30 kg/m^2^ was available for this endpoint. In the 30–34.9 kg/m^2^ BMI category, the SELECT trial^31^ reported a significant reduction of events with semaglutide, compared to placebo. Twenty‐one trials enroling patients with a mean BMI between 35 and 39.9 kg/m^2^ performed with liraglutide, semaglutide, tirzepatide, NB, and orlistat, which reported information on adjudicated MACE, failed to show any significant effect of any treatment on this endpoint. Only two RCTs with a mean BMI at enrolment >39.9 kg/m^2^ ^103^, ^129^ provided information on this endpoint, with no events reported (Figures 32S–34S).

*Hospitalisations for heart failure* (*HHF*). Only one trial enroling patients with mean BMI 30–34.9 kg/m^2^ reported information on this endpoint,^31^ showing a non‐significant reduction of HHF for semaglutide (Figure 11S, panel D). In 4 trials with mean BMI at enrolment between 35 and 39.9 kg/m^2^, both semaglutide (*N* = 3 studies) and tirzepatide (*N* = 1 study) were associated with a significant reduction of HHF (Figure 18S, panel I). No information on HHF was available for trials with mean BMI at enrolment below 30 or over 40 kg/m^2^ (Figures 32S–34S).

*Liver fibrosis and steato‐hepatitis*. Only two trials (one with semaglutide and one with tirzepatide) were conducted in patients with MASLD showing a superiority of tirzepatide, but not semaglutide, over placebo, for MASH remission and reduction of at least one stage of fibrosis in comparison with placebo (Figure 18S, panel L and M). Both trials enroled patients with a mean BMI between 35 and 35.9 kg/m^2^.

*OSAS*. Two trials with tirzepatide, enroling patients with a mean BMI between 35 and 35.9 kg/m^2^, reported information on OSAS, with significant beneficial effects of treatment (Figures 32S–34S).

*Diabetes incidence*. No information is available for trials enroling patients with mean BMI <30 kg/m^2^. Two trials with semaglutide with mean BMI at enrolment between 30 and 34.9 kg/m^2^ reported a significant reduction of the incidence of diabetes with the active treatment.^19^, ^155^ In trials enroling patients with a mean BMI between 35 and 39.9 kg/m^2^ (*N* = 4^19^, ^68^, ^83^, ^91^), a lower risk of incident diabetes was observed with liraglutide and orlistat, but not semaglutide. Only 2 studies with mean BMI at enrolment >39.9 kg/m^2^, one with semaglutide^101^ and one comparing RYGB and OAGB [131] reported data on incident diabetes, with no significant between‐group differences (Figures 32S–34S).

*Reversion to normoglycaemia*. Only one trial^33^ performed on patients with mean BMI 30–34 kg/m^2^ reported information on reversion to normoglycaemia (MH‐OR: 0.76, *p* = 0.003). In the 18 trials on patients with mean BMI 35–39.9 kg/m^2^ that reported information on diabetes remission, a formal NMA showed a remission rate with tirzepatide, RYGB, SG, semaglutide, LAGB, GCP, liraglutide, and NB, significantly higher than LSI/Pbo/NT. Eight trials with mean BMI at enrolment >39.9 kg/m^2^ reported information on reversion to normoglycaemia, failing to detect significant differences across treatments (Figures 32S–34S,^21^, ^25^, ^102^, ^108^, ^119^, ^129^, ^131^, ^145^).

#### 3.3.4 Serious adverse events (SAE)

*Total SAE*. No information on SAE was available from trials performed in patients with a mean BMI at enrolment <30 kg/m^2^. No significant increase in the risk of SAE was observed for any therapy in trials enroling patients with a mean BMI between 30 and 34.9 kg/m^2^ and comparing an active treatment with LSI/Pbo/NT (Figure 35S). In a NMA of trials with a mean BMI at enrolment between 35 and 39.9 kg/m^2^, MBS with the exception of SG and GCP was associated with the highest risk; ESG was also associated with an increased risk of SAE, unlike other types of EBP, whereas among OMMs only NB was associated with an increased risk of SAE (Figure 36S). In trials enroling patients with a mean BMI >40 kg/m^2^, BPD and SADI were the only treatments associated with an increase in the risk of overall SAE versus LSI/Pbo/NT (Figure 37S).

*Surgical SAE*. Data on surgical SAE are available for trials on MBS and EBP enroling patients with mean BMI 30–34.9, 35–39.9, and >40 kg/m^2^, but not for BMI <30 kg/m^2^. In trials with mean BMI 30–34.9 kg/m^2^, RYGB was associated with a statistically significant 28‐fold increased risk of surgical SAE versus lifestyle, with an estimated incidence of 14.2%; the corresponding figures for OAGB and SG were 6.7% and 3.3%, respectively, and a direct comparison between the two latter procedures did not detect significant differences in surgical SAE (Figure 35S).^37^, ^47^, ^48^, ^49^, ^153^ When analysing trials with mean BMI at enrolment between 35 and 39.9 kg/m^2^, ESG, IGB, but not POSE, were associated with an increased risk of periprocedural SAE; among MBS, LAGB, and RYGB, but not SG, were associated with an increased risk of surgical SAE, with SG showing a significantly lower risk of surgical SAE than other MBS in direct comparisons (Figure 36S). In trials with mean BMI at enrolment >40 kg/m^2^, SADI, BPD, LVGB, and RYGB were all associated with an increased risk of surgical SAE vs. LS/Pbo/No therapy (Figure 37S).

#### 3.3.5 All‐cause mortality

No data on all‐cause mortality is available for trials enroling patients with a mean BMI <30 kg/m^2^.

In a NMA of trials with mean BMI at enrolment 30–34.9 kg/m^2^, only semaglutide was associated with a significant reduction of all‐cause mortality versus LSI/Pbo/NT (Figure 35S). No significant effect on all‐cause mortality was detectable in trials with mean BMI at enrolment 35–39.9 kg/m^2^, or >40 kg/m^2^ (Figures 38S and 37S).

#### 3.3.6 Quality of life (QoL)

No data on quality of life is available for trials enroling patients with a mean BMI <30 kg/m^2^. In higher BMI categories, only a minority of trials reported quality of life results, using a variety of instruments, and therefore preventing a formal meta‐analysis. The most effective treatments (OMM or MBS) on weight loss were usually associated with improvements of QoL versus LSI/Pbo/NT in all BMI categories, whereas most direct comparisons between active treatments failed to detect significant differences (Figure 39S).

### 3.4 Risk of bias and confidence of evidence

The quality of evidence for trials with mean BMI at enrolment 30–34.9, 35–39.9, and >39.9 kg/m^2^ was moderate for both the primary endpoint and secondary endpoints with at least 10 studies (Table 13S). Figures 2S and 3S report review authors' judgements about each risk of bias item for each included study. On average, the included RCTs on OMMs are at low risk of bias, whereas those on EBP and MBS reported biases in several domains (i.e., selection and performance bias).

The certainty of the evidence evaluated by CINeMA for the primary endpoint (i.e., endpoint TBWL%) for all comparisons is presented in Figures 40S–42S of the Supplementary Materials. For class I of obesity, the confidence of evidence was high for all comparisons between OMMs and the reference category, and low or moderate for EBP and MBS. For class II of obesity, a high confidence of evidence was reported for tirzepatide, semaglutide, RYGB, and OAGB, whereas for all the other treatments the certainty of the evidence ranged from low to moderate. For class III of obesity, the confidence of evidence was moderate for all included treatments (all MBS), with the notable exception for semaglutide (‘high’).

## 4 DISCUSSION

The large majority of trials performed for assessing the efficacy of weight‐reducing treatments in subjects with obesity have relatively wide inclusion criteria, allowing for the enrolment of heterogeneous populations of individuals for body mass index. In fact, most studies on obesity management medications (OMM) include individuals with BMI greater than 27 kg/m^2^, with no upper limit, whereas many studies on surgical procedures were performed in individuals with BMI greater than 30 or 35 kg/m^2^. Even in larger scale trials, subgroup analyses for different classes of BMI are infrequently reported. As a consequence, a combined analysis of subgroups of BMI does not allow for drawing any conclusions on possible differential effects of treatments on different BMI classes, due to the paucity of data, which are limited to liraglutide and semaglutide.

An alternative approach for exploring the efficacy of treatments in subjects with different baseline BMI is that of analysing separately studies categorised for mean BMI at enrolment. This approach allows for the inclusion of many trials, although its reliability also has limitations. In fact, many trials enrol patients of different BMI classes, irrespective of mean BMI at study entry, producing a background noise which could blunt differences in efficacy dependent on baseline BMI.

Interestingly, even when categorising trials based on mean baseline BMI, the paucity of data for overweight (non‐obese) subjects persists. The only available data are those of a small subgroup in a trial with semaglutide and an Asian study performed on a population in which BMI cut‐offs for the diagnosis of obesity are different.^33^, ^156^ Although OMM are commonly indicated for individuals with BMI above 27 kg/m^2^ and comorbidities,^33^ evidence on their impact in individuals with BMI between 27 and 30 kg/m^2^ is scarce, not only for body weight reduction but also for concurrent metabolic abnormalities.

On the other hand, the number of trials with mean BMI at entry between 30 and 34.9 kg/m^2^ is substantial. In this category, where data on medications are more abundant than those on surgery, the efficacy of the most recent OMM, such as semaglutide and tirzepatide, is not inferior to surgical procedures. However, long‐term data are available only for semaglutide^157^ and RYGB.^152^ In trials with mean BMI at enrolment between 35 and 39.9 kg/m^2^, pharmacological and bariatric procedures were overall not different in terms of weight loss, with RYGB‐induced weight loss being most pronounced; on the other hand, bariatric procedures were associated with a considerably higher risk of SAE. Studies enroling patients with a mean BMI over 40 kg/m^2^ were mostly performed with surgical procedures, with BPD showing a greater efficacy and a higher incidence of adverse events than other treatments.

Overall, available data indicate that recent incretin‐mimetic OMMs could have a similar efficacy, at least in the short term, as surgical procedures in patients with BMI between 30 and 34.9 kg/m^2^. The impact of OMMs and MBS appears to be overall comparable also in patients with BMI between 35 and 39.9 kg/m^2^, with a notable exception for RYGB whose efficacy was highest among all treatments. However, these results should be considered with caution, because OMMs were mainly studied in patients with Class I obesity, whereas surgery was mostly studied in higher BMI categories, limiting the reliability of direct comparisons within the same BMI class. When considering higher SAE in surgical procedures and risk–benefit evaluation, the analyses suggest that preliminary preference could be given to pharmacological treatment in obesity class I and II, with the final decision based on individual patient characteristics and goals.

Beside their effects on body weight, treatments for obesity are primarily aimed at improving metabolic health and at reducing cardiovascular risk and comorbidities^158^, ^159^, ^160^, ^161^ Pre‐treatment BMI could theoretically moderate the efficacy of treatments on metabolic parameters and concurrent conditions. The analysis of the efficacy of different treatments on the reduction of diabetes risk and the increase of reversion to normoglycaemia in different BMI classes is problematic due to the relative paucity of data and the heterogeneity of populations enroled in different trials for diabetes prevalence and/or diabetes risk.

Weight loss is commonly associated with a reduction of triglyceride and an increase of HDL cholesterol^162^; this phenomenon is also observed in clinical trials, irrespective of baseline BMI. Orlistat appears to have a specific, beneficial effect on triglyceride and total cholesterol, as previously described.^163^ Similarly, the reduction of blood pressure is consistent with weight loss, with the notable exception of NB, which is associated with an increase of systolic blood pressure, as previously reported.^80^

At present, the only trial on treatments for obesity with major cardiovascular events (MACE) as the principal endpoint is the SELECT study, with semaglutide.^31^ For this study, a subgroup analysis was disclosed, failing to detect any difference in effects on MACE across BMI categories. The separate analysis of groups of trials with different classes of mean BMI at enrolment adds little information because of the small size of samples and the limited number of reported MACE. Among other cardiovascular outcomes, hospitalisations for heart failure appear to be reduced both by semaglutide and tirzepatide in the BMI class between 35 and 40 kg/m^2^, whereas data from trials with BMI at entry below 35 or over 40 kg/m^2^ are insufficient to draw any conclusion. Interestingly, in a pooled analysis of patient‐level data, semaglutide appeared to be effective in reducing hospitalisations for heart failure only in patients with BMI at enrolment greater than 35 kg/m^2^,^164^ suggesting that the efficacy of this molecule in improving symptoms of heart failure increases as a function of BMI.

Data on the effects of treatments on non‐cardiovascular complications of obesity, such as MASLD and OSAS, is still limited, and they do not allow for reliable analyses for different classes of BMI. An interim analysis of the ESSENCE trial with semaglutide, which was published after the literature search and therefore was not included in the present systematic review, reported beneficial effects on MASLD irrespective of baseline BMI; however, the samples in the lower classes of BMI were very small.^165^

The ultimate goals of treatment of obesity should be the reduction of all‐cause mortality and the improvement of quality of life. Unfortunately, available data on those two endpoints are too scarce to verify possible differences in the efficacy of treatment of obesity across different BMI categories. For all‐cause mortality, a significant improvement can be detected only for semaglutide in the BMI class between 30 and 35 kg/m^2^, but the result is largely driven by a single trial^31^ and data in different BMI classes are sparse. Quality of life is often overlooked in trials on obesity, and the heterogeneity of instruments for its assessment prevents any reliable analysis combining the results of different trials in the same class of BMI.

Some limitations of the present systematic review should be considered when interpreting the results. The main limitation is represented by the use of mean BMI at enrolment of trials, meaning that some RCTs considered in an obesity category can likely also include patients with BMI different from that category; the resulting analyses can only approximately give information on each individual class of obesity, differently from those obtained by prespecified subgroup analyses. This approach has an intrinsic further limitation, due to the validity of BMI itself, which is being criticised as a single tool for obesity diagnosis and classification.^166^, ^167^ However, BMI remains the key parameter for obesity classification in existing RCTs, and a key diagnostic tool in clinical practice, and it may represent here a useful tool to separate large patient categories with different overall clinical needs and optimal treatment options.

The quality of trials is not homogeneous, possibly introducing some biases. The open‐label design, which is inevitable in the case of comparisons between surgical and non‐surgical treatments, could produce a bias because of a possible placebo effect of surgery. Moreover, the reference category used for NMA is heterogeneous, including placebo, lifestyle interventions, and no therapy. This is due to the fact that most RCTs on OMMs are placebo‐controlled, whereas EBP and MBS are often compared to lifestyle interventions or no therapy. For these reasons, we decided to avoid any formal statistical comparison (i.e., performing Surface Under the Cumulative Ranking curve—SUCRA—to rank treatments) across different strategies. The certainty of the evidence evaluated by CINeMA for the primary endpoint (i.e., endpoint TBWL%) for all comparisons was generally high for all comparisons between OMMs and the reference category; on the contrary, the confidence of evidence was rated ‘low’ or ‘moderate’ for the majority of EBP and MBS comparisons. This imbalance in the quality of evidence across different anti‐obesity strategies could limit the reliability of the present NMA.

Another relevant limitation of NMAs included in the present systematic review is represented by inconsistency: NMA aims at combining trial evidence to estimate the relative differences between several interventions within a connected network. In this case, this is obtained by making the consistency assumption that the relative treatment effect between two anti‐obesity strategies ‘x’ and ‘y’ is the difference between the effect of treatments ‘x’ and ‘y’ relative to LSI/Pbo/NT. By combining trial evidence in a NMA, we assume that trial populations are fairly homogeneous, so as to be combined; this assumption, however, is problematic due to relevant differences in case mix across different trials. Although we did not observe relevant inconsistency for any of the principal analyses performed (*H* values <3), the results obtained should be interpreted with caution.

Further limitations include the lack of data on long‐term adherence to treatments, the analysis of outcomes different from the principal endpoint of individual trials (e.g., lipid levels or diabetes incidence in trials primarily aimed at assessing weight loss), and the paucity of data on some relevant endpoints (e.g., OSAS, knee osteoarthritis, etc.), and some minor differences in the outcome definitions (e.g., reversion to normoglycaemia was defined as HbA1c <6.0% and <5.7% in STEP 10 and SURPASS‐1 trial, respectively).

## 5 CONCLUSION

In patients affected by mild to moderate obesity, newer OMMs (i.e., tirzepatide and semaglutide) appear to be valid alternatives to EBP and MBS and could be preliminarily chosen as a first‐line option based on similar efficacy (at least in the short term) and greater safety and tolerability. Higher degrees of obesity could be more effectively treated with MBS, the efficacy of which, with the notable exception of LAGB and GCP, appears superior to other treatments, especially in the long term. Some types of MBS, such as BPD and SADI, although very effective, should be used with caution because of safety issues, whereas RYGB and LSG combine good efficacy with greater safety.

These results are of interest to clinicians involved in the management of obesity. For the first time, performing a thorough evaluation and synthesis of RCTs and adopting GRADE methodology, different anti‐obesity approaches have been meta‐analysed in different categories of patients (overweight, and obesity class I, II, and III), providing a clearer picture of their effectiveness. A systematic disclosure of results in different classes of BMI would enhance our knowledge of the profile of action of different treatments, allowing for a more rational choice of therapy in individual patients.

## FUNDING INFORMATION

This research was performed as a part of the institutional activity of the unit, with no specific funding. All expenses, including the salaries of the investigators, were covered by public research funds assigned to the unit. The manuscript was drafted and revised by the authors following ICJME standards for authorship. The corresponding author had full access to all the data in the study and had final responsibility for the decision to submit it for publication. The funders (scientific societies: IFSO and SICOB) had no role in data collection, analysis, interpretation, or writing of the manuscript and the decision to submit it.

## CONFLICT OF INTEREST STATEMENT

Matteo Monami has received speaking fees from Astra Zeneca, Bristol Myers Squibb, Boehringer‐Ingelheim, Eli‐Lilly, Merck, Novo Nordisk, Sanofi, and Novartis and research grants from Bristol Myers Squibb. Edoardo Mannucci has received consultancy fees from Merck and Novartis, speaking fees from Astra Zeneca, Bristol Myers Squibb, Boehringer‐Ingelheim, Eli‐Lilly, Merck, Novo Nordisk, Sanofi, and Novartis, and research grants from Merck, Novartis, and Takeda. Maurizio De Luca reports grants from Johnson and Johnson, grants from Medtronic, and consultancy fees from Novo Nordisk. Luca Busetto received payment of honoraria from EliLilly, Novo Nordisk, Boehringer Ingheleim, Pfizer and Regeneron as a member of advisory boards, and payment of honoraria for lectures from Rhytms Pharmaceuticals. Paolo Sbraccia received payment of honoraria from Lilly, Novo Nordisk, Boehringer Ingheleim, Pfizer, Amryt (Chiesi) as a member of advisory boards and payment of honoraria for lectures from Lilly, Novo Nordisk, Amryt (Chiesi). Rocco Barazzoni received payment of honoraria from Boehringer Ingheleim and EliLilly. Giovanni Antonio Silverii received payment of honoraria from EliLilly. Amanda Belluzzi, Giuseppe Navarra, Benedetta Ragghianti, and Silvio Buscemi do not have any conflicts of interest to disclose. All the authors approved the final version of this manuscript. Dr. Matteo Monami is the person who takes full responsibility for the work as a whole, including the study design, access to data, and the decision to submit and publish the manuscript.

## ETHICS STATEMENT

Not applicable.

## Supporting information


**Data S1.** Supplementary Information.

## Data Availability

All references are linked to the dataset in Supplementary Information. Data sharing not applicable to this article as no datasets were generated or analysed during the current study.

## Endnote

^d^

OAGB, SG, and RYGB showed a proportion of patients experiencing at least one surgical SAE of 6.5%, 3.3%, and 17.2%, respectively.
