# Supplementary material for: Efficacy and safety of European Medicines Agency (EMA)‐approved pharmacological, endoscopic, and surgical treatments in different classes of obesity: A network meta‐analysis of randomised controlled trials for the development of the SIO (Società Italiana Obesità) Italian guidelines for the diagnosis and treatment of overweight and obesity
Source: Diabetes Obes Metab. 2025 Oct 20;28(1):358–78. doi: 10.1111/dom.70204 (PMC12673438; doi:10.1111/dom.70204)
Supplement: Supplementary file 1 — Data S1. Supplementary Information. [file DOM-28-358-s001.docx]

**Supplementary appendix**

**Supplement to:**

**Efficacy and Safety of EMA (European Medicines Agency)-Approved Pharmacological, Endoscopic, and Surgical Treatments in different classes of obesity: a Network Meta-Analysis of Randomized Controlled Trials.**

*Rocco Barazzoni^1,a^, Matteo Monami^2,a^, Silvio Buscemi^3^, Luca Busetto^4^, Maurizio De Luca^5^, Giuseppe Navarra^6^, Benedetta Ragghianti^2^, Giovanni Antonio Silverii^2^, Amanda Belluzzi^5^, Edoardo Mannucci^2^, Paolo Sbraccia^7^.*

**Table of contents**

**Figures**

[Figure 1S – Trial flow summary 8](#_Toc209627771)

[Figure 2S – Risk of bias graph: review authors' judgements about each risk of bias item presented as percentages across all included studies. 9](#_Toc209627772)

[Figure 3S – Risk of bias summary: review authors' judgements about each risk of bias item for each included study. 10](#_Toc209627773)

[Figure 4S – Effects of individual anti-obesity strategy on TBWL% at 26-52 (Panel A: network plot; Panel B: forest plot) and 52-103 weeks in placebo (no therapy; Panel A) and active- (Panel C) controlled trials in studies with mean BMI at entry ranging from 30 to 34,9 kg/m](#_Toc209627775)^[2](#_Toc209627775)^[. The node size represents the quantity of entities or participants, while the edge (line) thickness indicates the strength or frequency of the connection between them, such as the number of studies or data points assessing the relationship 11](#_Toc209627775)

[Figure 5S – Heterogeneity (variation in treatment effects across studies) for each comparison included in the NMA for endpoint TBWL% in class I of obesity (i.e., studies with a mean BMI at entry ranging from 30 to 34.9 kg/m](#_Toc209627776)^[2](#_Toc209627776)^[). 13](#_Toc209627776)

[Figure 6S – Inconsistency test with node-splitting model for comparisons reporting direct and indirect evidence and a mean BMI at entry ranging from 30 to 34.9 kg/m2. 14](#_Toc209627778)

[Figure 7S – Funnel plot for TBWL% at the endpoint for trials either versus placebo or standard of care with a mean BMI at entry ranging from 30 to 34.9 kg/m](#_Toc209627779)^[2](#_Toc209627779)^[. 15](#_Toc209627779)

[Figure 8S – Effects of different treatments on endpoint BMI (kg/m](#_Toc209627780)^[2](#_Toc209627780)^[) in placebo- (Panel A) and active-controlled trials (Panel B) in trials with mean BMI at entry ranging from 30 to 34,9 kg/m](#_Toc209627780)^[2](#_Toc209627780)^[. 16](#_Toc209627780)

[Figure 9S – Effects of different treatments on endpoint waist circumference (cm) in placebo (no therapy)-controlled trials with mean BMI at entry ranging from 30 to 34,9 kg/m](#_Toc209627781)^[2](#_Toc209627781)^[. 18](#_Toc209627781)

[Figure 10S – Effects of individual anti-obesity strategy on TBWL% at 26-52 (Panel A: network plot; Panel B: forest plot), 52-103 weeks in placebo (Panel C: network plot; Panel D: forest plot), 104-156 (Panel E: versus LSI/Pbo/No therapy, and Panel F: head-to-head comparisons), >156 weeks (Panel G: versus LSI/Pbo/No therapy, and Panel H: head-to-head comparisons) in studies with mean BMI at entry ranging from 35 to 39.9 kg/m](#_Toc209627782)^[2](#_Toc209627782)^[. The node size  represents the quantity of entities or participants, while the edge (line) thickness indicates the strength or frequency of the connection between them, such as the number of studies or data points assessing the relationship 19](#_Toc209627782)

[Figure 11S – Effects of individual anti-obesity strategy on BMI and waist circumference at the endpoint (Panel A and B: network plot; Panel C and D) in studies with mean BMI at entry ranging from 35 to 39.9 kg/m](#_Toc209627783)^[2](#_Toc209627783)^[. The node size  represents the quantity of entities or participants, while the edge (line) thickness indicates the strength or frequency of the connection between them, such as the number of studies or data points assessing the relationship 22](#_Toc209627783)

[Figure 12S – Heterogeneity (variation in treatment effects across studies) for each comparison included in the NMA for endpoint TBWL% in class II of obesity (i.e., studies with a mean BMI at entry ranging from 35 to 39.9 kg/m](#_Toc209627784)^[2](#_Toc209627784)^[). 24](#_Toc209627784)

[Figure 13S – Inconsistency test with node-splitting model for comparisons reporting direct and indirect evidence and a mean BMI at entry ranging from 35 to 39.9 kg/m](#_Toc209627785)^[2](#_Toc209627785)^[. 25](#_Toc209627785)

[Figure 14S – Funnel plot for TBWL% at the endpoint for trials either versus placebo or standard of care with a mean BMI at entry ranging from 35 to 39.9 kg/m](#_Toc209627786)^[2](#_Toc209627786)^[. 26](#_Toc209627786)

[Figure 15S – Effects of individual anti-obesity strategy on TBWL% at 26-52 (Panel A: network plot; Panel B: forest plot), 52-103 weeks in placebo (Panel C: network plot; Panel D: forest plot), 104-156 (Panel E: network plot; Panel F: forest plot), 157-260 weeks (Panel G: network plot; Panel H: forest plot), and 261-520 weeks (Panel I: network plot; Panel L: forest plot) in studies with mean BMI at entry> 39.9 kg/m](#_Toc209627787)^[2](#_Toc209627787)^[. The node size  represents the quantity of entities or participants, while the edge (line) thickness indicates the strength or frequency of the connection between them, such as the number of studies or data points assessing the relationship 27](#_Toc209627787)

[Figure 16S – Effects of individual anti-obesity strategy on BMI (Panel A: network plot; Panel B: forest plot) and waist circumference (Panel C: network plot; Panel D: forest plot) at the endpoint in studies with mean BMI at entry > 39.9 kg/m](#_Toc209627788)^[2](#_Toc209627788)^[. The node size represents the quantity of entities or participants, while the edge (line) thickness indicates the strength or frequency of the connection between them, such as the number of studies or data points assessing the relationship 30](#_Toc209627788)

[Figure 17S – Heterogeneity (variation in treatment effects across studies) for each comparison included in the NMA for endpoint TBWL% in class III of obesity (i.e., studies with a mean BMI at entry >39.9 kg/m](#_Toc209627789)^[2](#_Toc209627789)^[). 32](#_Toc209627789)

[Figure 18S – Inconsistency test with node-splitting model for comparisons reporting direct and indirect evidence and a mean BMI at entry ranging >39.9 kg/m](#_Toc209627790)^[2](#_Toc209627790)^[. 33](#_Toc209627790)

[Figure 19S – Network meta-regression plots for class I obesity: all relative treatment effects vs reference treatment with confidence regions and study contributions to the treatment-by-covariate interaction parameters. A: mean age (covariate value: 48 years); B: mean BMI (covariate value: 33.2 kg/m](#_Toc209627791)^[2](#_Toc209627791)^[); C: proportion of women (covariate value 68%). 34](#_Toc209627791)

[Figure 20S – Network meta-regression plots for class II obesity: all relative treatment effects vs reference treatment with confidence regions and study contributions to the treatment-by-covariate interaction parameters. A: mean age (covariate value: 48 years); B: mean BMI (covariate value: 36.8 kg/m](#_Toc209627792)^[2](#_Toc209627792)^[); C: proportion of women (covariate value 68%). 35](#_Toc209627792)

[Figure 21S – Network meta-regression plots for class III obesity: all relative treatment effects vs reference treatment with confidence regions and study contributions to the treatment-by-covariate interaction parameters. A: mean age (covariate value: 44 years); B: mean BMI (covariate value: 45.3 kg/m](#_Toc209627793)^[2](#_Toc209627793)^[); C: proportion of women (covariate value 68 %). 36](#_Toc209627793)

[Figure 22S – Forest plot for TBWL% at the endpoint (Panel A: 30 to 34.9 kg/m](#_Toc209627794)^[2](#_Toc209627794)^[; Panel B: 35 to 39.9 kg/m](#_Toc209627794)^[2](#_Toc209627794)^[; Panel C: >39.9 kg/m](#_Toc209627794)^[2](#_Toc209627794)^[) after excluding low-quality trials (rob: risk of bias). 37](#_Toc209627794)

[Figure 23S – Effects of different treatments on endpoint HbA1c (mmol/mol) in Pbo/LSI/no therapy-controlled trials with mean BMI at entry ranging from 30 to 34,9 kg/m](#_Toc209627795)^[2](#_Toc209627795)^ [(Panel A: network plot; Panel B: forest plot). The node size represents the quantity of entities or participants, while the edge (line) thickness indicates the strength or frequency of the connection between them, such as the number of studies or data points assessing the relationship 37](#_Toc209627795)

[Figure 24S – Effects of different treatments on endpoint FPG (mg/dl) in Pbo/LSI/no therapy-controlled trials with mean BMI at entry ranging from 30 to 34.9 kg/m](#_Toc209627797)^[2](#_Toc209627797)^ [(Panel A: network plot; Panel B: forest plot). The node size represents the quantity of entities or participants, while the edge (line) thickness indicates the strength or frequency of the connection between them, such as the number of studies or data points assessing the relationship 39](#_Toc209627797)

[Figure 25S – Effects of individual anti-obesity strategy on HbA1](#_Toc209627799)_[c](#_Toc209627799)_ [and FPG at the endpoint (Panel A and C: network plots for HbA1c and FPG, respectively; Panel B and D: forest plots for HbA1c and FPG, respectively) in studies with mean BMI at entry ranging from 35 to 39.9 kg/m](#_Toc209627799)^[2](#_Toc209627799)^[. The node size  represents the quantity of entities or participants, while the edge (line) thickness indicates the strength or frequency of the connection between them, such as the number of studies or data points assessing the relationship 40](#_Toc209627799)

[Figure 26S – Effects of individual anti-obesity strategy on HbA1](#_Toc209627800)_[c](#_Toc209627800)_ [(and FPG at the endpoint (Panel A and C: network plots for HbA1c and FPG, respectively; Panel B and D: forest plots for HbA1c and FPG, respectively; Panel B1 and D1: forest plots for HbA1c and FPG in RCTs enrolling patients with diabetes, respectively) in studies with mean BMI at entry > 39.9 kg/m](#_Toc209627800)^[2](#_Toc209627800)^[.](#_Toc209627800)[The node size  represents the quantity of entities or participants, while the edge (line) thickness indicates the strength or frequency of the connection between them, such as the number of studies or data points assessing the relationship 42](#_Toc209627800)

[Figure 27S – Effects of different treatments on endpoint total (Panel A; mg/dl), HDL-cholesterol (Panel B; mg/dl) , and triglycerides (Panel C; mg/dl) in Pbo/LSI/no therapy-controlled trials with mean BMI at entry ranging from 30 to 34,9 kg/m](#_Toc209627801)^[2](#_Toc209627801)^[. The node size represents the quantity of entities or participants, while the edge (line) thickness indicates the strength or frequency of the connection between them, such as the number of studies or data points assessing the relationship 44](#_Toc209627801)

[Figure 28S – Effects of individual anti-obesity strategy on total cholesterol (Panel A: network plot and B: forest plot), HDL-cholesterol (Panel C: network plot and D: forest plot), and triglycerides (Panel E: network plot and F: forest plot) at the endpoint in studies with mean BMI at entry ranging from 35 to 39.9 kg/m](#_Toc209627802)^[2](#_Toc209627802)^[. The node size  represents the quantity of entities or participants, while the edge (line) thickness indicates the strength or frequency of the connection between them, such as the number of studies or data points assessing the relationship 46](#_Toc209627802)

[Figure 29S – Effects of individual anti-obesity strategy on total cholesterol (Panel A: network plot and B: forest plot), HDL-cholesterol (Panel C: network plot and D: forest plot), and triglycerides (Panel E: network plot and F: forest plot) at the endpoint in studies with mean BMI at entry >39.9 kg/m](#_Toc209627803)^[2](#_Toc209627803)^[. The node size  represents the quantity of entities or participants, while the edge (line) thickness indicates the strength or frequency of the connection between them, such as the number of studies or data points assessing the relationship 48](#_Toc209627803)

[Figure 30S – Effects of individual anti-obesity strategy on systolic (Panel A: network plot and Panel B: forest plot) and diastolic blood pressure systolic (Panel C: network plot and Panel D: forest plot) in studies with mean BMI at entry ranging from 35 to 39.9 kg/m](#_Toc209627804)^[2](#_Toc209627804)^[. The node size  represents the quantity of entities or participants, while the edge (line) thickness indicates the strength or frequency of the connection between them, such as the number of studies or data points assessing the relationship 50](#_Toc209627804)

[Figure 31S – Effects of individual anti-obesity strategy on systolic (Panel A: network plot and Panel B: forest plot) and diastolic blood pressure systolic (Panel C: network plot and Panel D: forest plot) in studies with mean BMI at entry >39.9 kg/m](#_Toc209627805)^[2](#_Toc209627805)^[. The node size  represents the quantity of entities or participants, while the edge (line) thickness indicates the strength or frequency of the connection between them, such as the number of studies or data points assessing the relationship 52](#_Toc209627805)

[Figure 32S – Effects of different treatments on OAMC remission (Panel A: MACE; Panel B: diabetes remission; Panel C: incident diabetes; Panel D: Hospitalization for heart failure; E: all-cause mortality) in Pbo/LSI/no therapy-controlled trials with mean BMI at entry ranging from 30 to 34.9 kg/m2. 53](#_Toc209627806)

[Figure 33S – Effects of different treatments on OAMC remission (Panel A: MACE; Panel B and C: diabetes remission; Panel D and E: hypertension remission; Panel F and G: dyslipidemia remission; Panel H: incident diabetes; Panel I: Hospitalization for heart failure; Panel L: Liver fibrosis (reduction of at least one stage); Panel M: MASH remission with no worsening of fibrosis; Panel N: OSAS remission: Apnea-Ipopnea Index< 5 events/hour) in Pbo/LSI/no therapy-controlled trials with mean BMI at entry ranging from 35 to 39.9 kg/m](#_Toc209627807)^[2](#_Toc209627807)^[. The node size  represents the quantity of entities or participants, while the edge (line) thickness indicates the strength or frequency of the connection between them, such as the number of studies or data points assessing the relationship 55](#_Toc209627807)

[Figure 34S – Effects of different treatments on OAMC remission (Panel A and B; diabetes remission; Panel C and D: incident diabetes; Panel E: hypertension remission; Panel F: dyslipidemia remission; Panel G: osteoarthritis remission; Panel H: OSAS remission: Apnea-Ipopnea Index< 5 events/hour) in Pbo/LSI/no therapy-controlled trials with mean BMI at entry >39.9 kg/m](#_Toc209627808)^[2](#_Toc209627808)^[. 59](#_Toc209627808)

[Figure 35S – Effects of different anti-obesity strategies on surgical (Panel A: versus LSI/Pbo/No therapy; Panel B: head-to-head comparisons) and overall SAE (Panel C: versus LSI/Pbo/No therapy) at the endpoint in RCTs with a mean BMI at entry 30-34.9 Kg/m2. SG: Sleeve Gastrectomy; OAGB: One-anastomosis gastric bypass; RYGB: Roux-en-Y Gastric By-Pass. 63](#_Toc209627809)

[Figure 36S – Effects of different treatments on surgical SAE (Panel A: versus LSI/Pbo/No therapy; Panel B: head-to-head comparisons) and overall SAE (Panel C: network plot; Panel D: forest plot) in trials with mean BMI at entry ranging from 35 to 39.9 kg/m](#_Toc209627810)^[2](#_Toc209627810)^[. The node size  represents the quantity of entities or participants, while the edge (line) thickness indicates the strength or frequency of the connection between them, such as the number of studies or data points assessing the relationship 65](#_Toc209627810)

[Figure 37S – Effects of different treatments on overall SAE (Panel A: versus LSI/Pbo/No therapy; Panel B: head-to-head comparisons), surgical SAE (Panel C: network plot; Panel D: forest plot), and all-cause mortality (Panel E and F), in trials with mean BMI at entry > 39.9 kg/m](#_Toc209627811)^[2](#_Toc209627811)^[. The node size  represents the quantity of entities or participants, while the edge (line) thickness indicates the strength or frequency of the connection between them, such as the number of studies or data points assessing the relationship 68](#_Toc209627811)

[Figure 38S – Effects of different treatments on all-cause mortality (Panel A: network plot; Panel B: forest plot) in trials with mean BMI at entry ranging from 35 to 39.9 kg/m](#_Toc209627812)^[2](#_Toc209627812)^[. The node size  represents the quantity of entities or participants, while the edge (line) thickness indicates the strength or frequency of the connection between them, such as the number of studies or data points assessing the relationship 70](#_Toc209627812)

[Figure 39S – Effects of different treatments on quality of life at the endpoint (Panel A: IWQOL Lite; Panel B: SF-36 General Helath; Panel C: SF-36 Physical Functioning) in trials with mean BMI at entry ranging from 35 to 39.9 kg/m](#_Toc209627813)^[2](#_Toc209627813)^[. 71](#_Toc209627813)

[Figure 40 – Overall NMA risk of bias (within-study and reporting bias, indirectness, imprecision, heterogeneity, and incoherence) for each comparison, including studies with a mean BMI at entry ranging from 30 to 34.9 kg/m2 (Panel A), 35 to 39.9 kg/m2 (Panel B), and >39.9 kg/m2 (Panel C) versus the reference category (i.e., LSI/Placebo/None). 73](#_Toc209627814)

**[Tables](#_Toc209627815)** [74](#_Toc209627815)

[Table 1S – PRISMA checklist. 74](#_Toc209627816)

[Table 2S – Detailed information on search strategy 78](#_Toc209627817)

[Table 3S. Information collected for each trial 82](#_Toc209627818)

[Table 4S. Excluded trials and reasons for the exclusion. 83](#_Toc209627819)

[Table 5S. Principal baseline characteristics of the included studies 84](#_Toc209627820)

[Table 7S – Pairwise comparison table for TBWL% at the endpoint for class I of obesity. 91](#_Toc209627821)

[Table 8S – Assessment of inconsistency for all comparisons for TBWL% at the endpoint in class I of obesity. 92](#_Toc209627822)

[Diff.: difference; CI: Confidence intervals; L/P/None; Lifestyle interventions/Placebo/No interventions. IGB: Intragastric Balloon; RYGB: Rou-en-Y Gastric By-pass; OAGB: One-Anastomosis Gastric By-pass; SG: Sleeve Gastrectomy. 92](#_Toc209627823)

[Table 10S – Pairwise comparison table for TBWL% at the endpoint for class II of obesity. 95](#_Toc209627824)

[Table 11S – Assessment of inconsistency for all comparisons for TBWL% at the endpoint in class II of obesity. 96](#_Toc209627825)

[Diff.: difference; CI: Confidence intervals; L/P/None; Lifestyle interventions/Placebo/No interventions. IGB: Intragastric Balloon; RYGB: Rou-en-Y Gastric By-pass; OAGB: One-Anastomosis Gastric By-pass; SG: Sleeve Gastrectomy. 96](#_Toc209627826)

[Table 13S – Pairwise comparison table for TBWL% at the endpoint for class III of obesity. 100](#_Toc209627827)

[Table 14S – Assessment of inconsistency for all comparisons for TBWL% at the endpoint in class III of obesity. 101](#_Toc209627828)

[Table 12S – GRADE evaluation of retrieved evidence for the primary endpoint (i.e., TBWL% at the endpoint) for studies versus LSI/Pbo/No therapy. 103](#_Toc209627829)

[Table 13S – GRADE evaluation of retrieved evidence for the secondary endpoints with at least 10 studies versus LSI/Pbo/No therapy. 104](#_Toc209627830)

**[References](#_Toc209627831)** [106](#_Toc209627831)

# Figure 1S – Trial flow summary


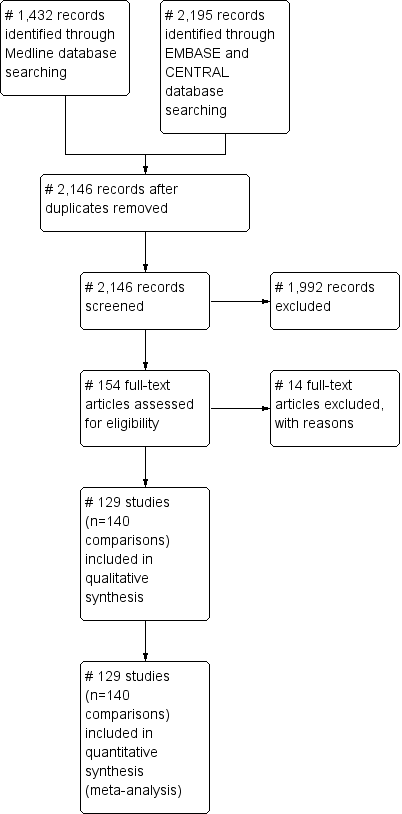


# Figure 2S – Risk of bias graph: review authors' judgements about each risk of bias item presented as percentages across all included studies.

**
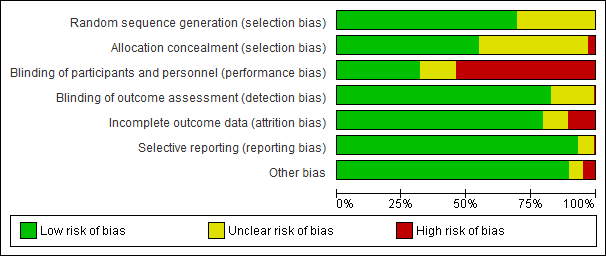
**

#
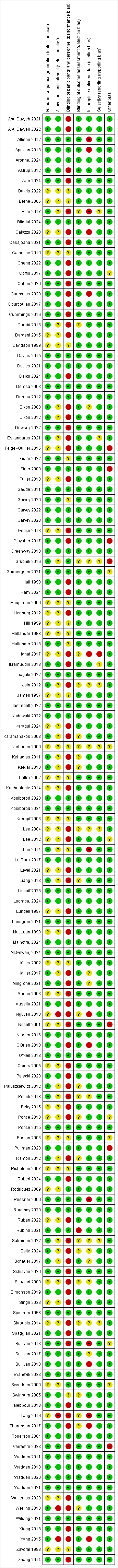

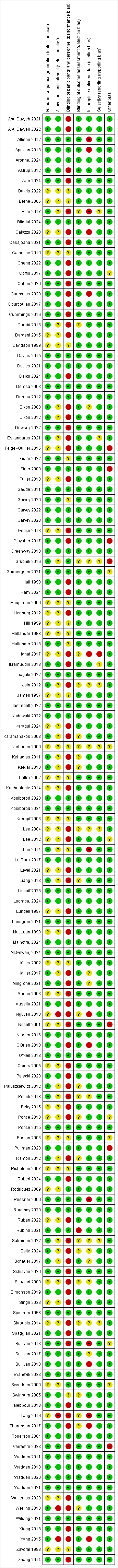
Figure 3S – Risk of bias summary: review authors' judgements about each risk of bias item for each included study.

**
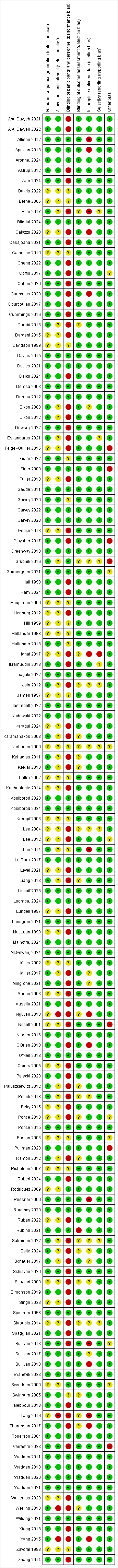
**

## Trials with mean BMI at entry ranging from 30 to 34.9 kg/m^2^

# Figure 4S – Effects of individual anti-obesity strategy on TBWL% at 26-52 (Panel A: network plot; Panel B: forest plot) and 52-103 weeks in placebo (no therapy; Panel A) and active- (Panel C) controlled trials in studies with mean BMI at entry ranging from 30 to 34,9 kg/m^2^. The node size represents the quantity of entities or participants, while the edge (line) thickness indicates the strength or frequency of the connection between them, such as the number of studies or data points assessing the relationship

**A**

**
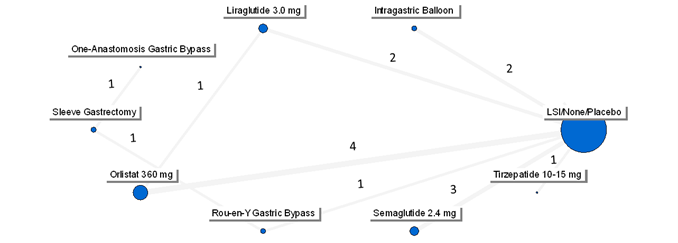
**

**B**

**
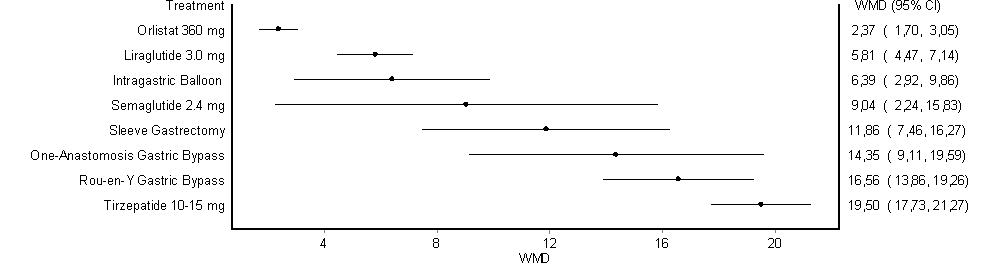
**

**B**

**
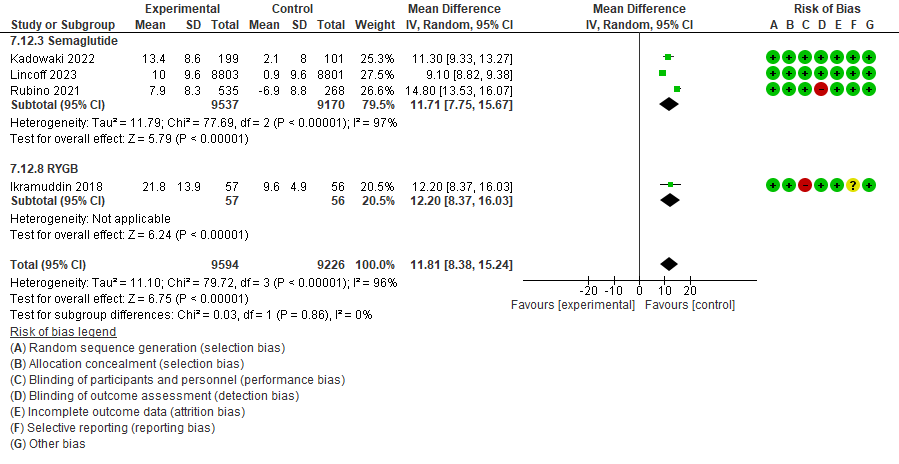
**

**B**

Gastric by-pass versus SG

**
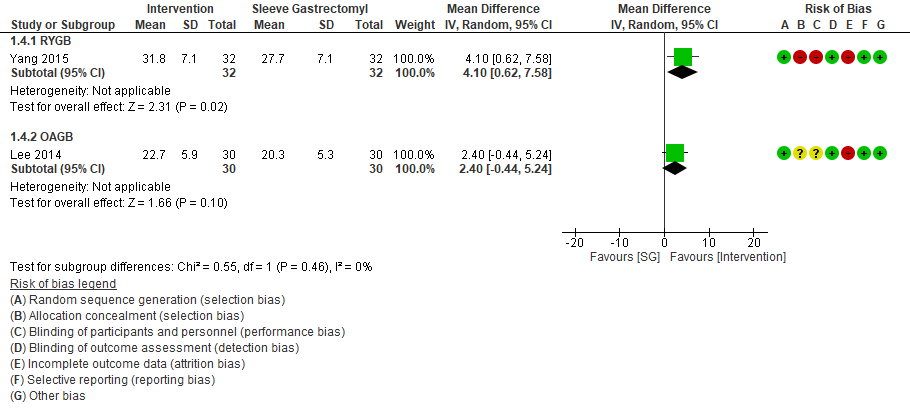
**

# Figure 5S – Heterogeneity (variation in treatment effects across studies) for each comparison included in the NMA for endpoint TBWL% in class I of obesity (i.e., studies with a mean BMI at entry ranging from 30 to 34.9 kg/m^2^).

# **
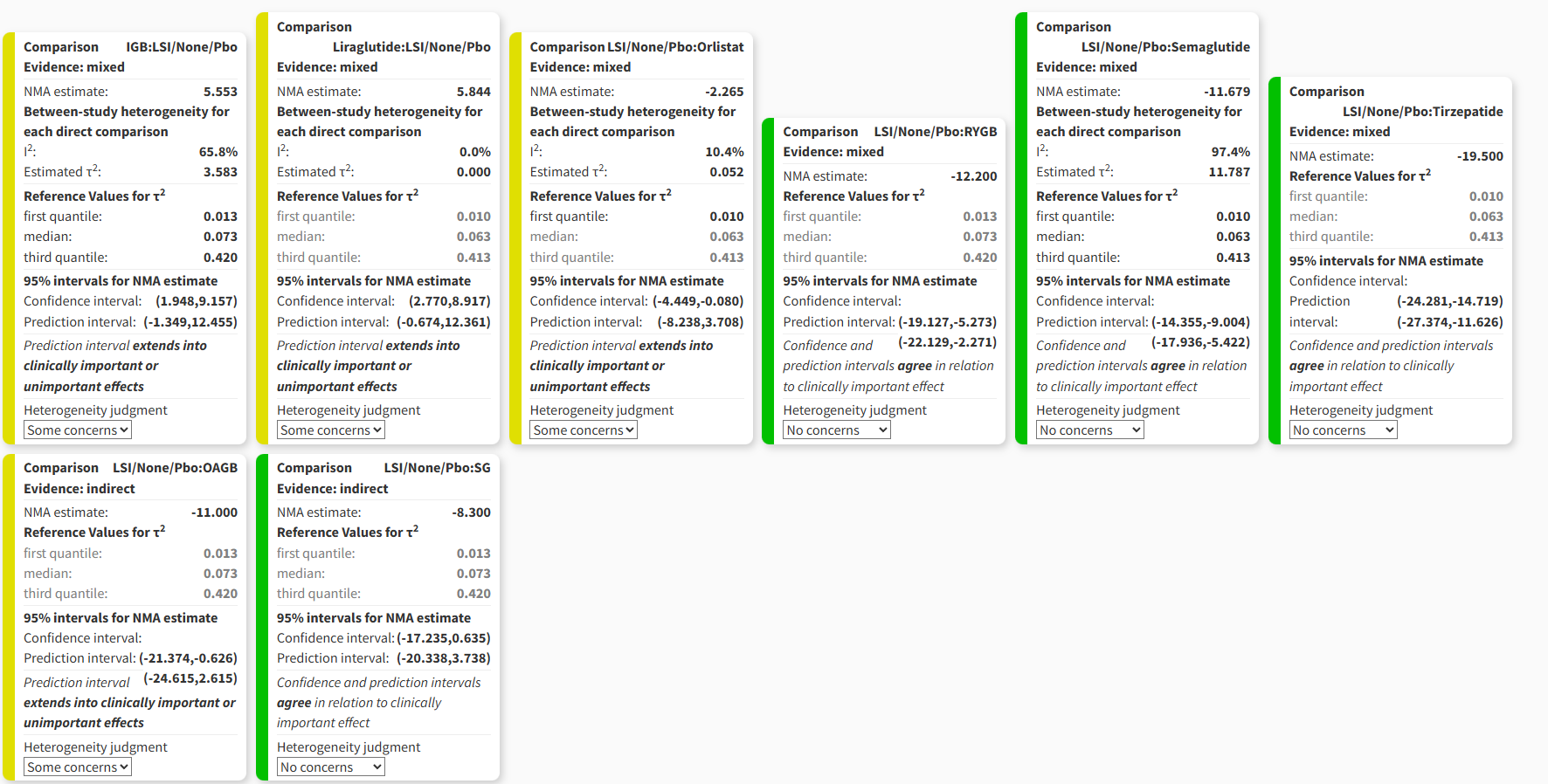
**

# Figure 6S – Inconsistency test with node-splitting model for comparisons reporting direct and indirect evidence and a mean BMI at entry ranging from 30 to 34.9 kg/m2.

**
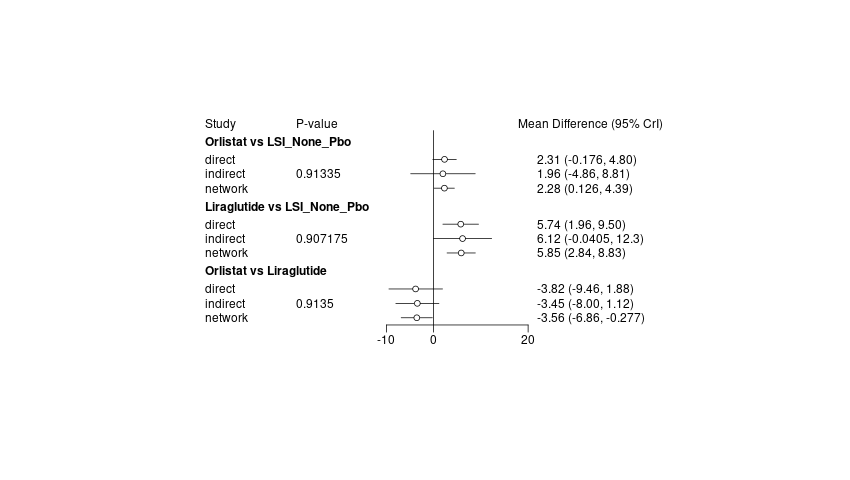
**

# Figure 7S – Funnel plot for TBWL% at the endpoint for trials either versus placebo or standard of care with a mean BMI at entry ranging from 30 to 34.9 kg/m^2^.


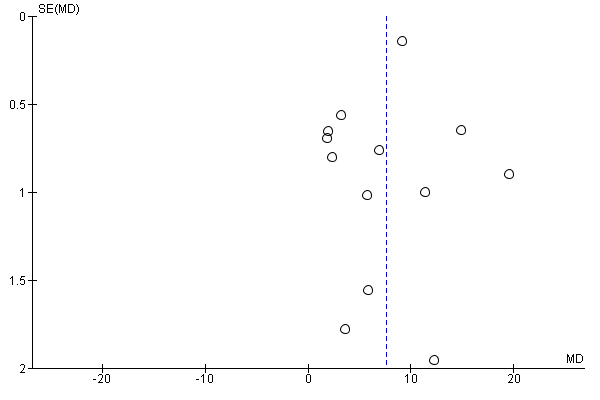


# Figure 8S – Effects of different treatments on endpoint BMI (kg/m^2^) in placebo- (Panel A) and active-controlled trials (Panel B) in trials with mean BMI at entry ranging from 30 to 34,9 kg/m^2^.

**A. Placebo (no therapy)-controlled trials**

**
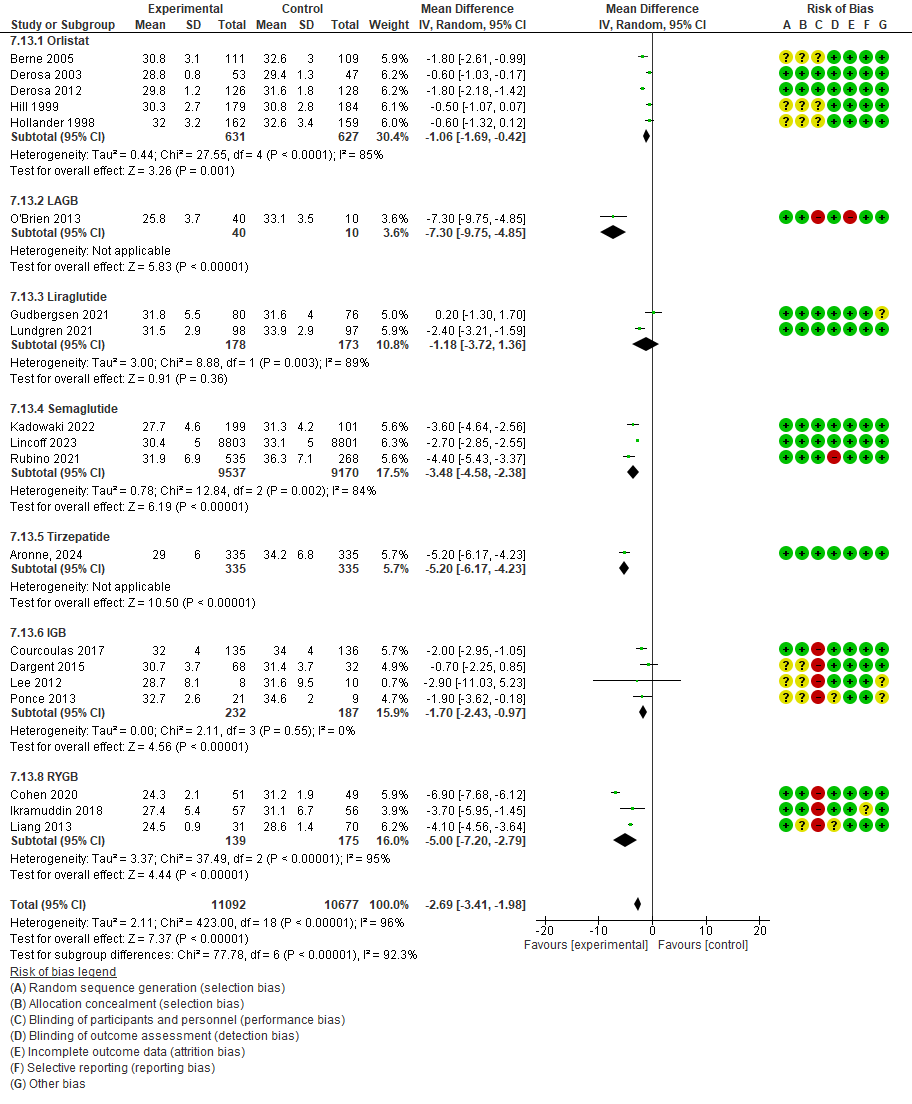
**

**B. Active-controlled trials**

**
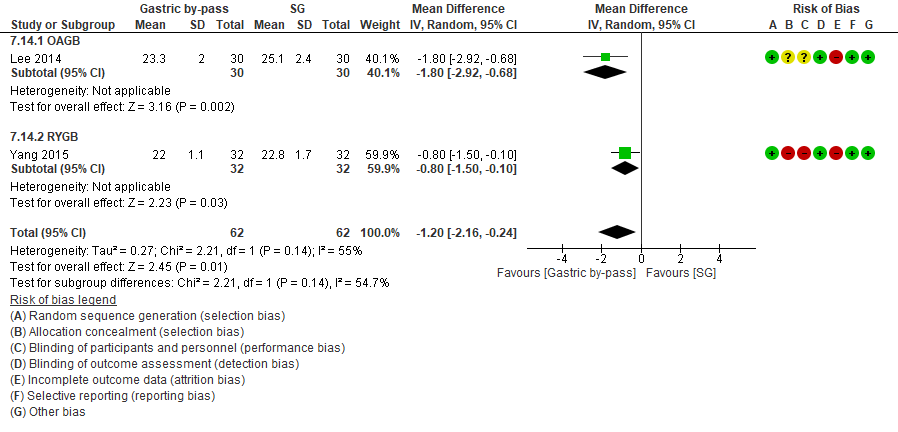
**

# Figure 9S – Effects of different treatments on endpoint waist circumference (cm) in placebo (no therapy)-controlled trials with mean BMI at entry ranging from 30 to 34,9 kg/m^2^.


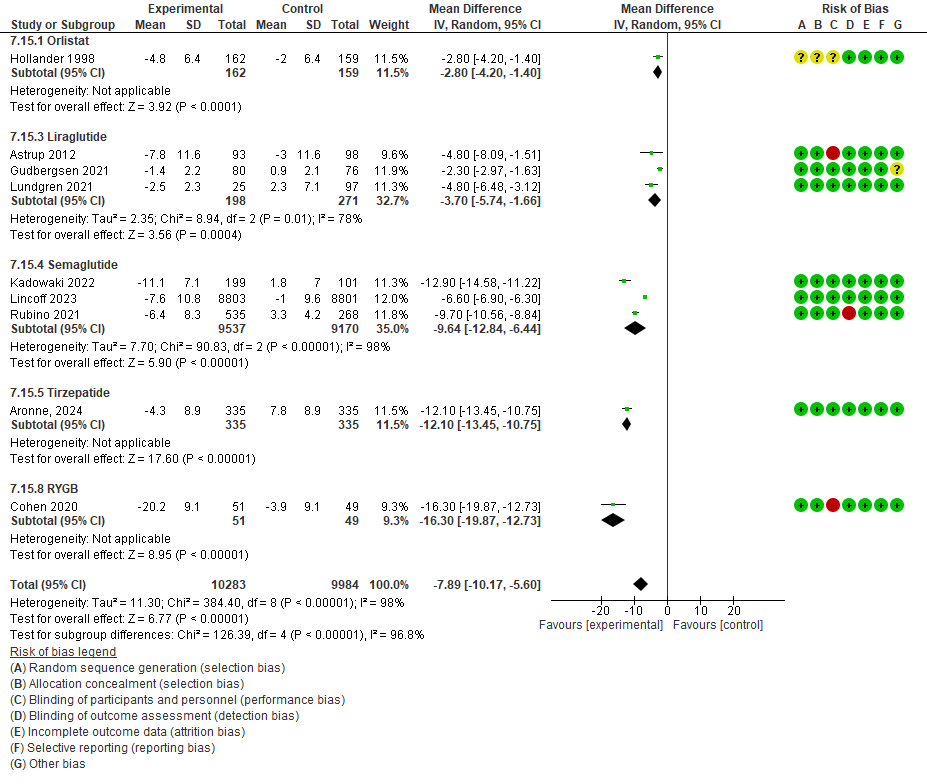


# 0

# 1

# Figure 12S – Heterogeneity (variation in treatment effects across studies) for each comparison included in the NMA for endpoint TBWL% in class II of obesity (i.e., studies with a mean BMI at entry ranging from 35 to 39.9 kg/m^2^).

**
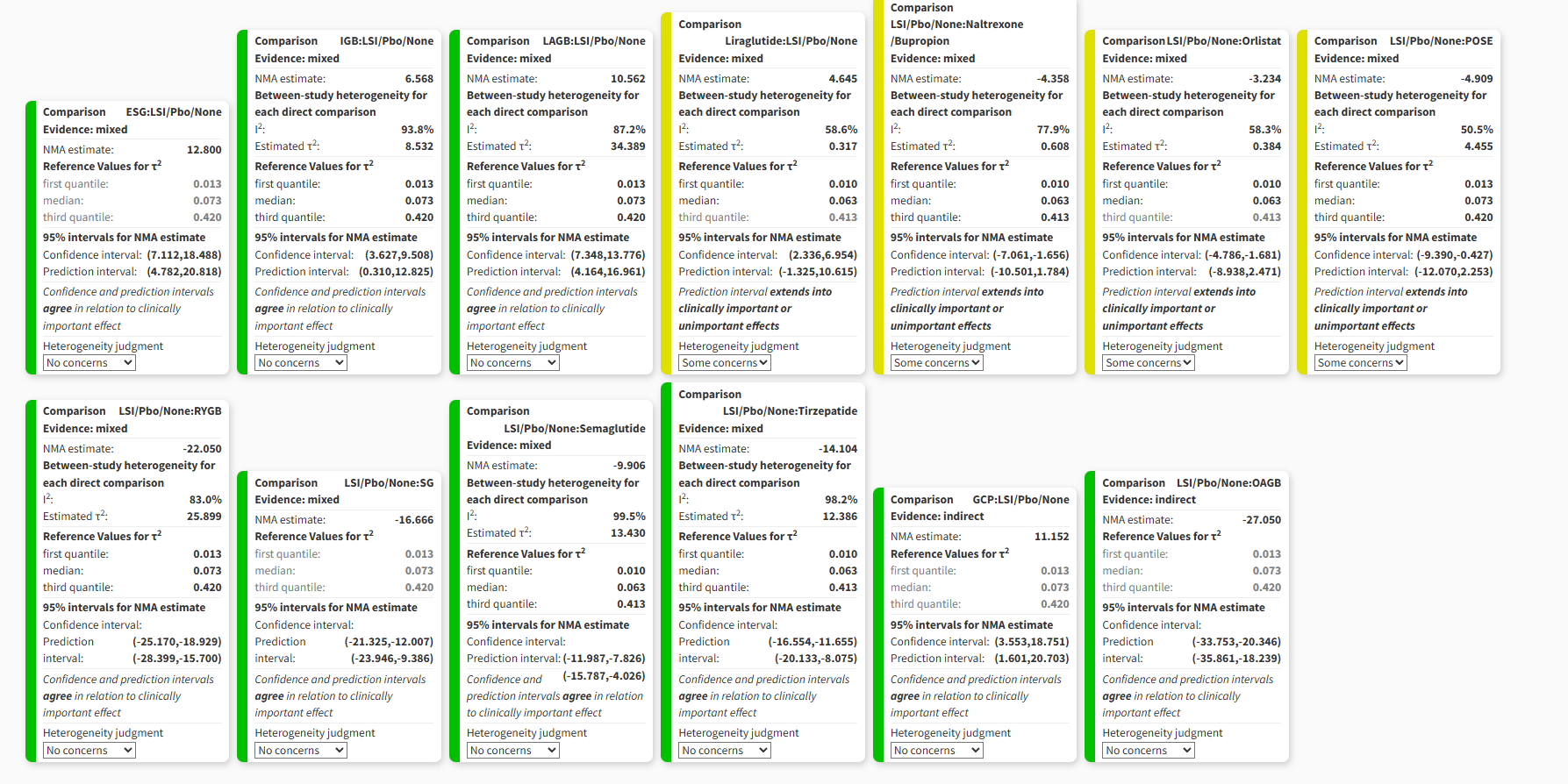
**

# Figure 13S – Inconsistency test with node-splitting model for comparisons reporting direct and indirect evidence and a mean BMI at entry ranging from 35 to 39.9 kg/m^2^.

**
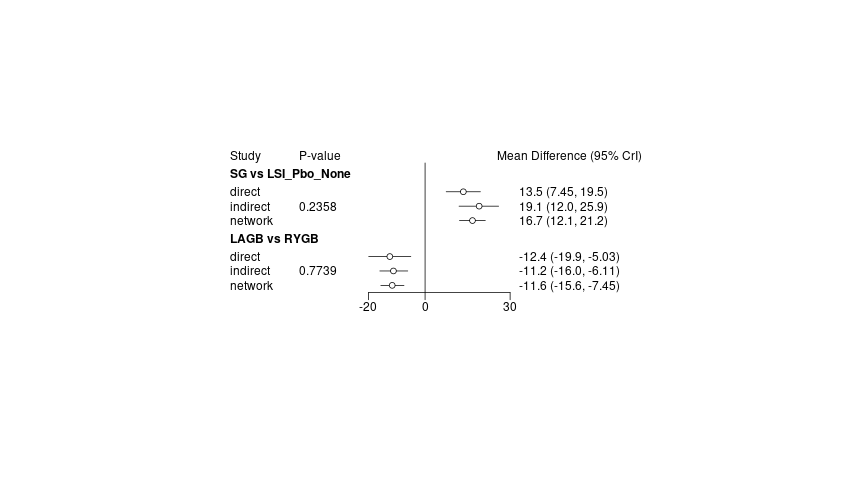
**

# Figure 14S – Funnel plot for TBWL% at the endpoint for trials either versus placebo or standard of care with a mean BMI at entry ranging from 35 to 39.9 kg/m^2^.


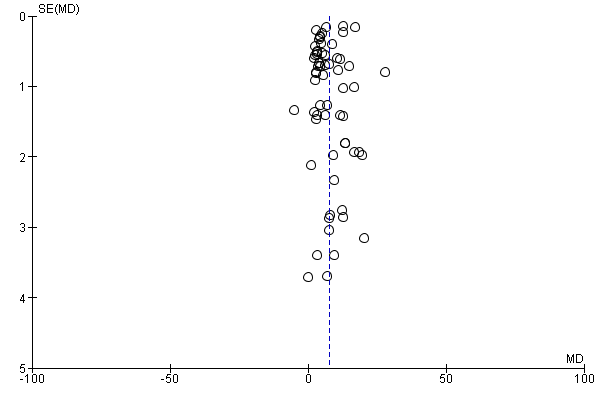


# 15S

# 16

# Figure 17S – Heterogeneity (variation in treatment effects across studies) for each comparison included in the NMA for endpoint TBWL% in class III of obesity (i.e., studies with a mean BMI at entry >39.9 kg/m^2^).

**
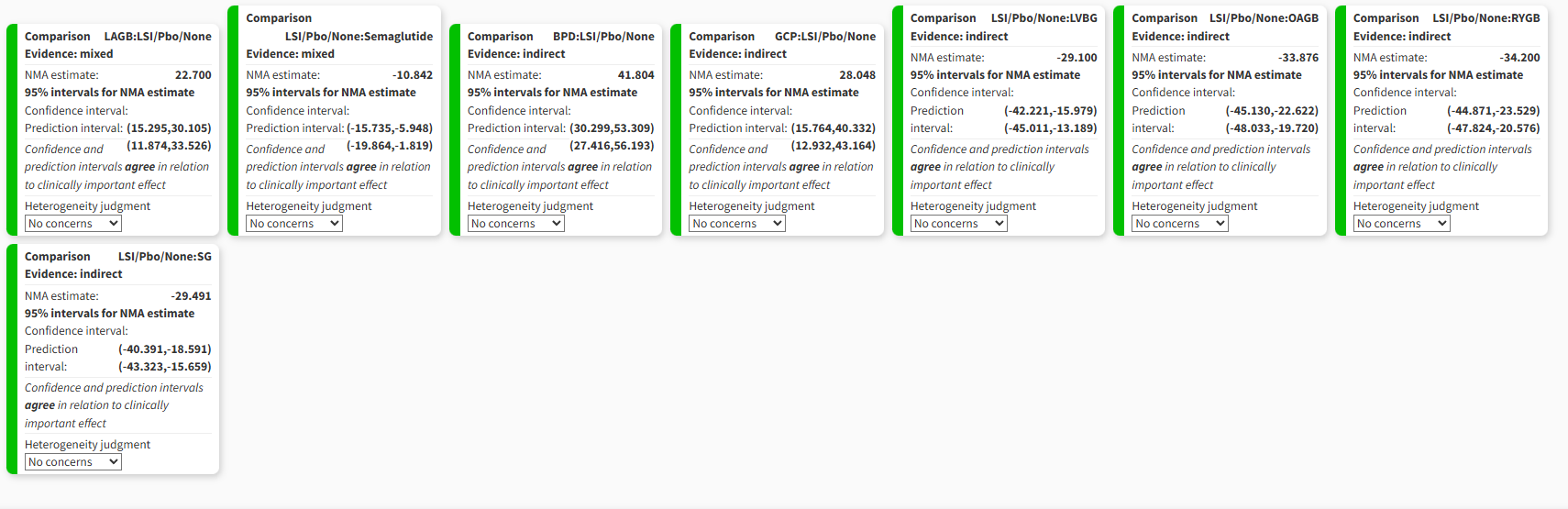
**

# Figure 18S – Inconsistency test with node-splitting model for comparisons reporting direct and indirect evidence and a mean BMI at entry ranging >39.9 kg/m^2^.

**
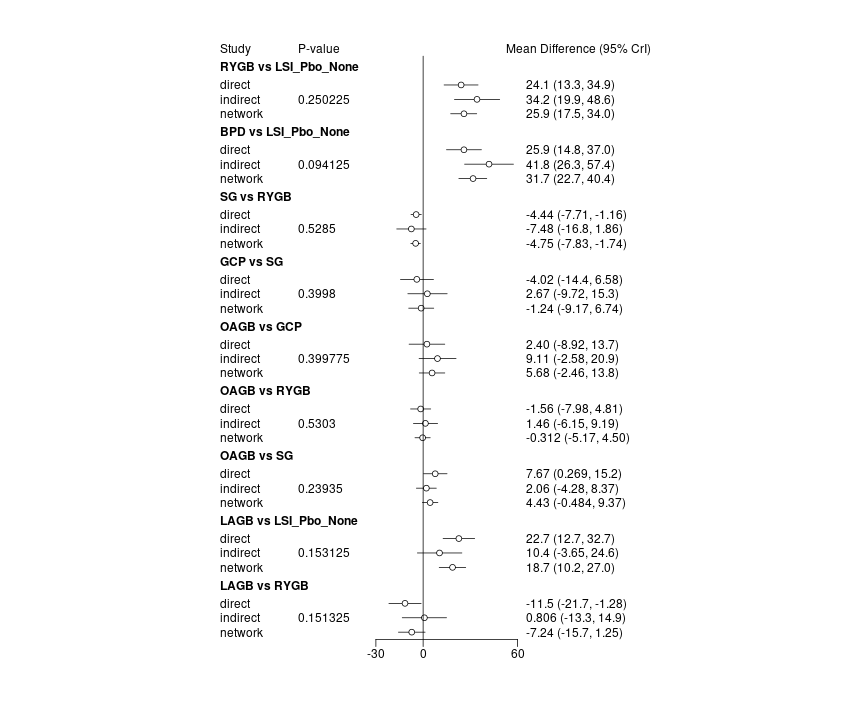
**

# Figure 19S – Network meta-regression plots for class I obesity: all relative treatment effects vs reference treatment with confidence regions and study contributions to the treatment-by-covariate interaction parameters. A: mean age (covariate value: 48 years); B: mean BMI (covariate value: 33.2 kg/m^2^); C: proportion of women (covariate value 68%).

**A: Age**


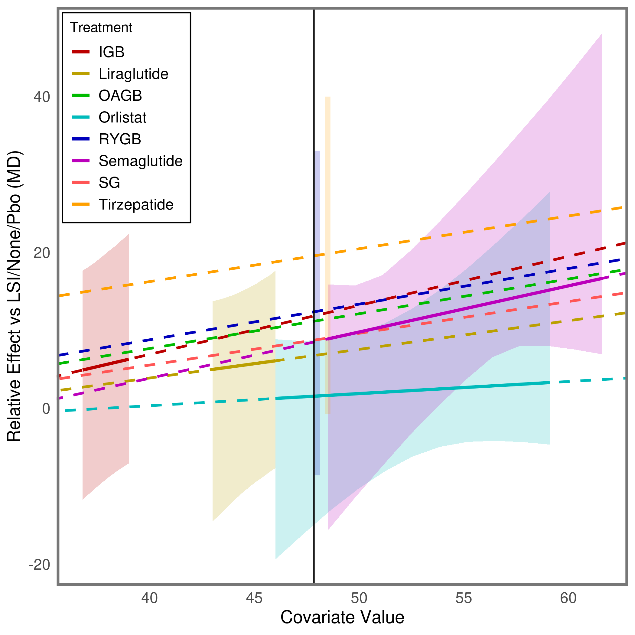


**B: BMI**

**
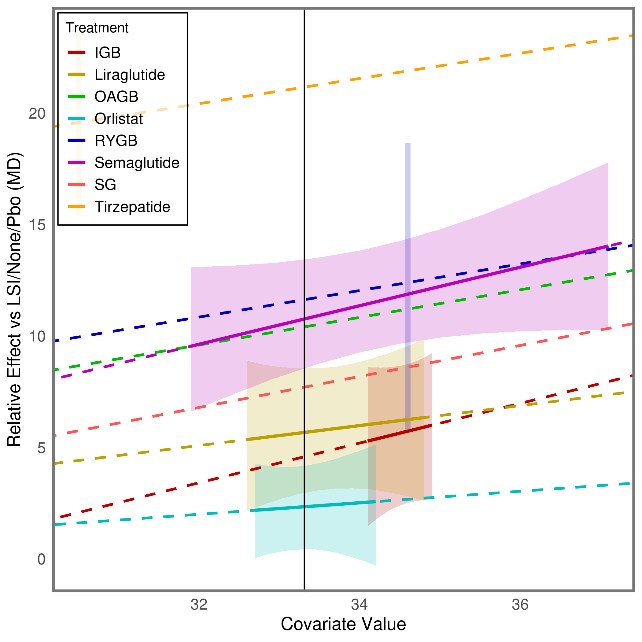
**

**C: Proportion of women**

**
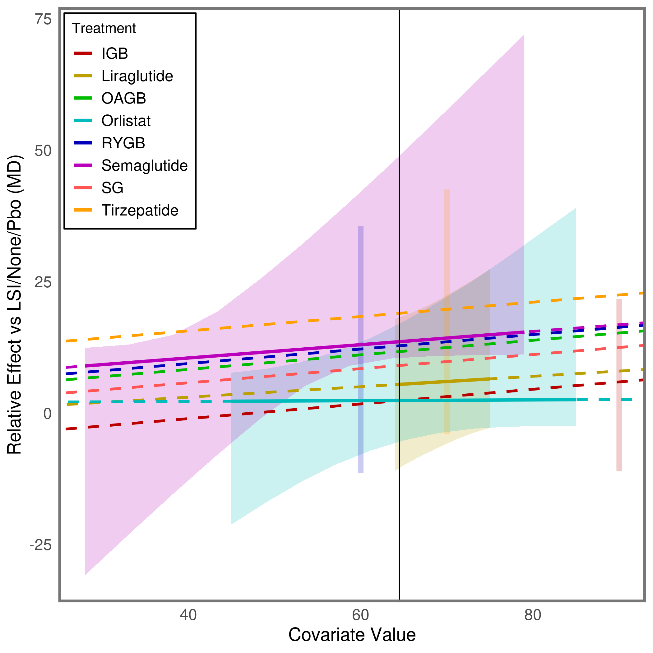
**

# Figure 20S – Network meta-regression plots for class II obesity: all relative treatment effects vs reference treatment with confidence regions and study contributions to the treatment-by-covariate interaction parameters. A: mean age (covariate value: 48 years); B: mean BMI (covariate value: 36.8 kg/m^2^); C: proportion of women (covariate value 68%).

**A: Age**

**
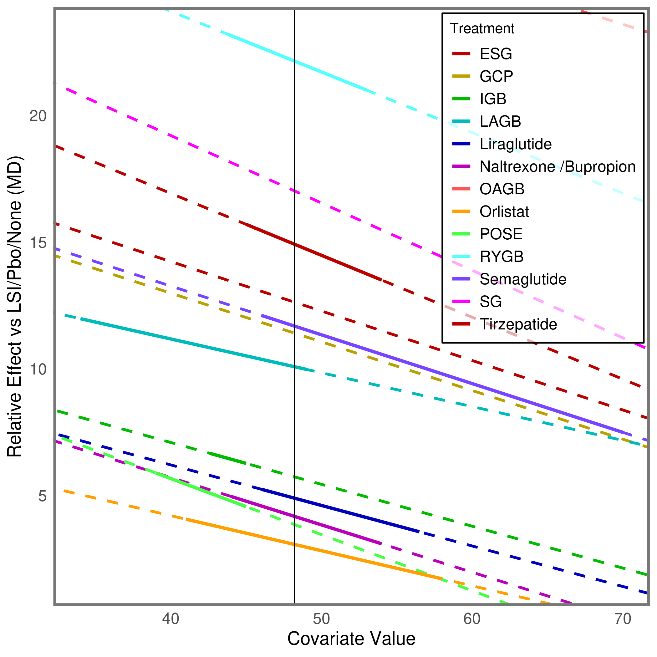
**

**B: BMI**

**
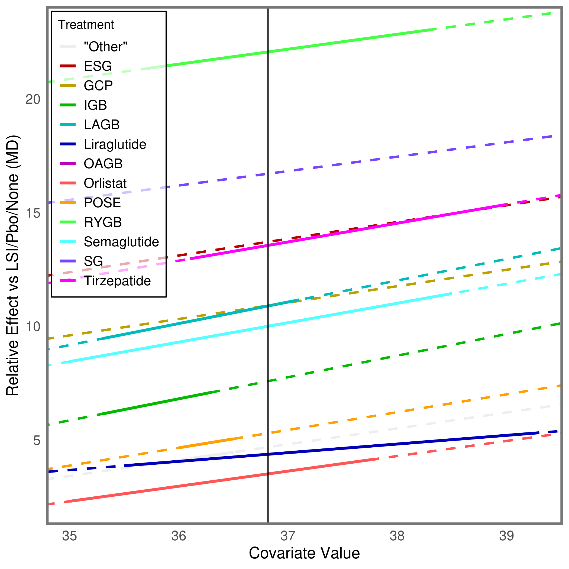
**

**C: Proportion of women**


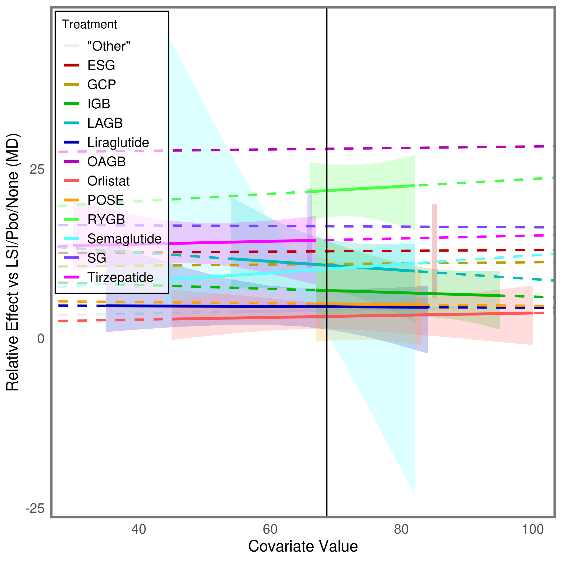


# Figure 21S – Network meta-regression plots for class III obesity: all relative treatment effects vs reference treatment with confidence regions and study contributions to the treatment-by-covariate interaction parameters. A: mean age (covariate value: 44 years); B: mean BMI (covariate value: 45.3 kg/m^2^); C: proportion of women (covariate value 68 %).

**A: Age**

**
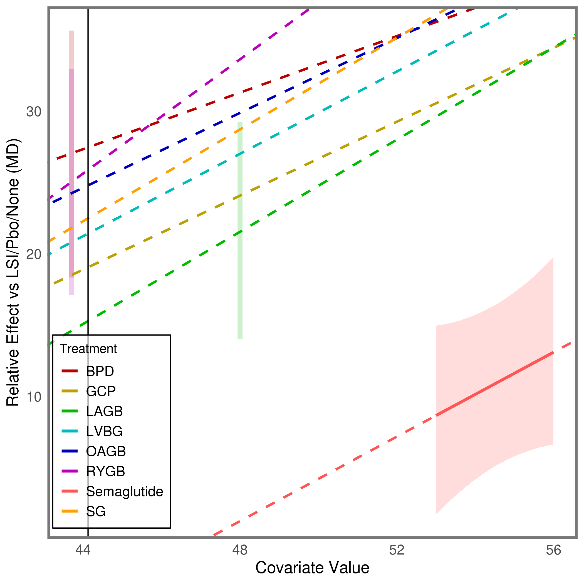
**

**B: BMI**

**
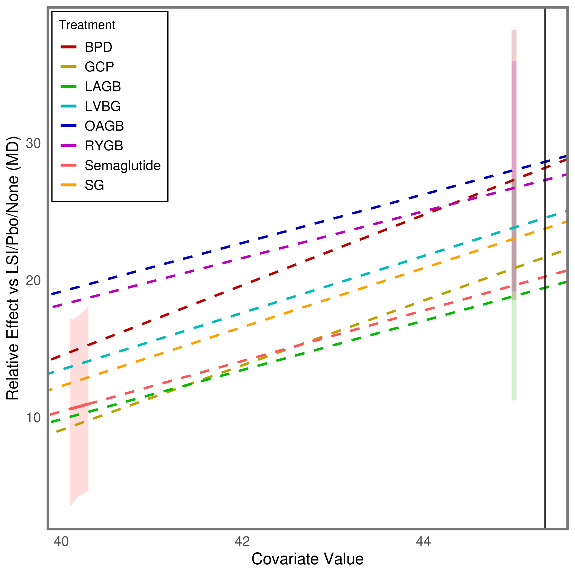
**

**C: Proportion of women**


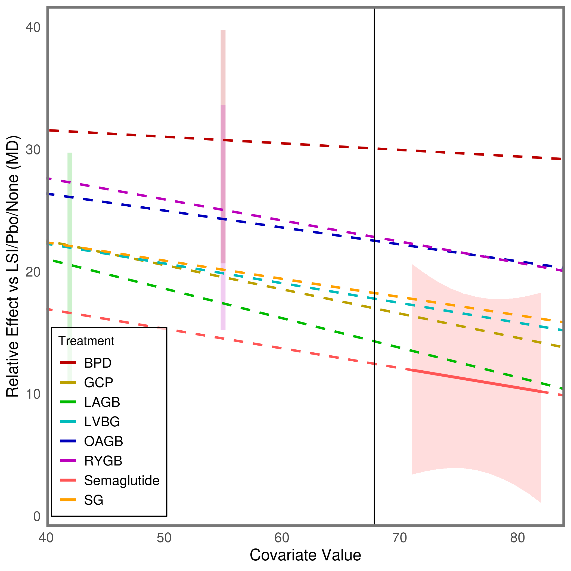


# Figure 22S – Forest plot for TBWL% at the endpoint (Panel A: 30 to 34.9 kg/m^2^; Panel B: 35 to 39.9 kg/m^2^; Panel C: >39.9 kg/m^2^) after excluding low-quality trials (rob: risk of bias).

A


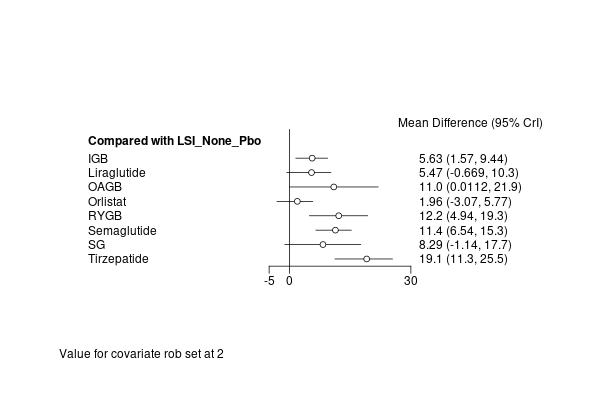


**B**

*
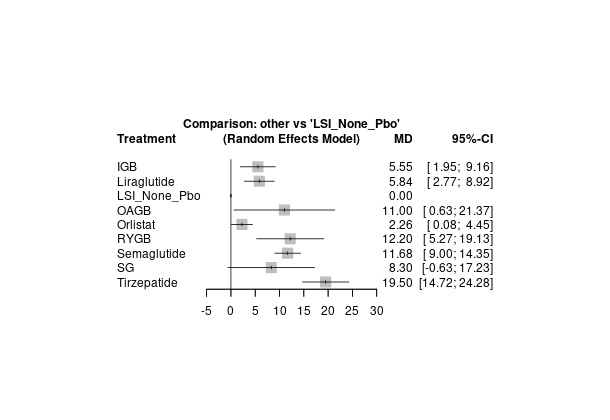
*

**C**

#
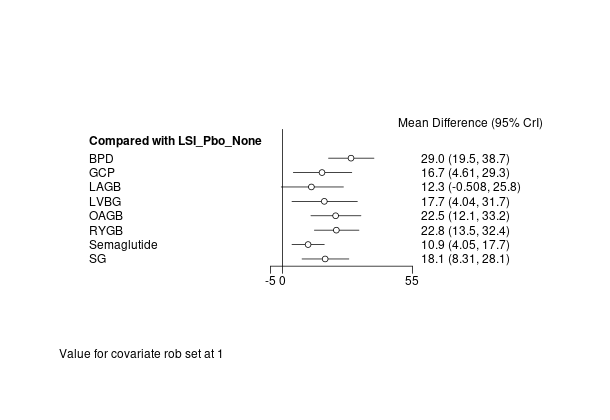
Figure 23S – Effects of different treatments on endpoint HbA1c (mmol/mol) in Pbo/LSI/no therapy-controlled trials with mean BMI at entry ranging from 30 to 34,9 kg/m^2^ (Panel A: network plot; Panel B: forest plot). The node size represents the quantity of entities or participants, while the edge (line) thickness indicates the strength or frequency of the connection between them, such as the number of studies or data points assessing the relationship

**A**


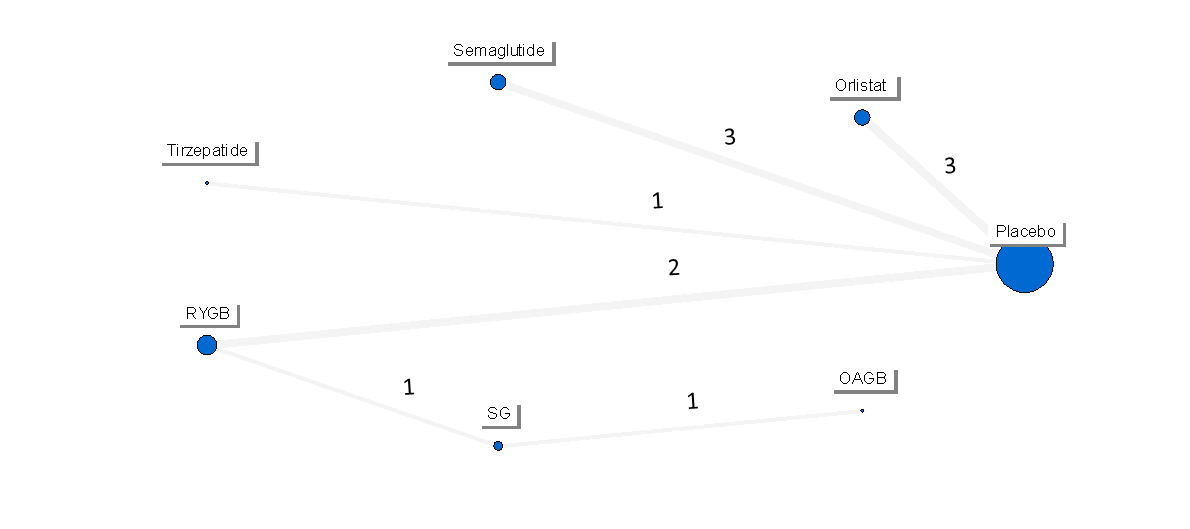


B

# Figure 24S – Effects of different treatments on endpoint FPG (mg/dl) in Pbo/LSI/no therapy-controlled trials with mean BMI at entry ranging from 30 to 34.9 kg/m^2^ (Panel A: network plot; Panel B: forest plot). The node size represents the quantity of entities or participants, while the edge (line) thickness indicates the strength or frequency of the connection between them, such as the number of studies or data points assessing the relationship

**A**

B

# Figure 25S – Effects of individual anti-obesity strategy on HbA1_c_ and FPG at the endpoint (Panel A and C: network plots for HbA1c and FPG, respectively; Panel B and D: forest plots for HbA1c and FPG, respectively) in studies with mean BMI at entry ranging from 35 to 39.9 kg/m^2^. The node size  represents the quantity of entities or participants, while the edge (line) thickness indicates the strength or frequency of the connection between them, such as the number of studies or data points assessing the relationship

**A**


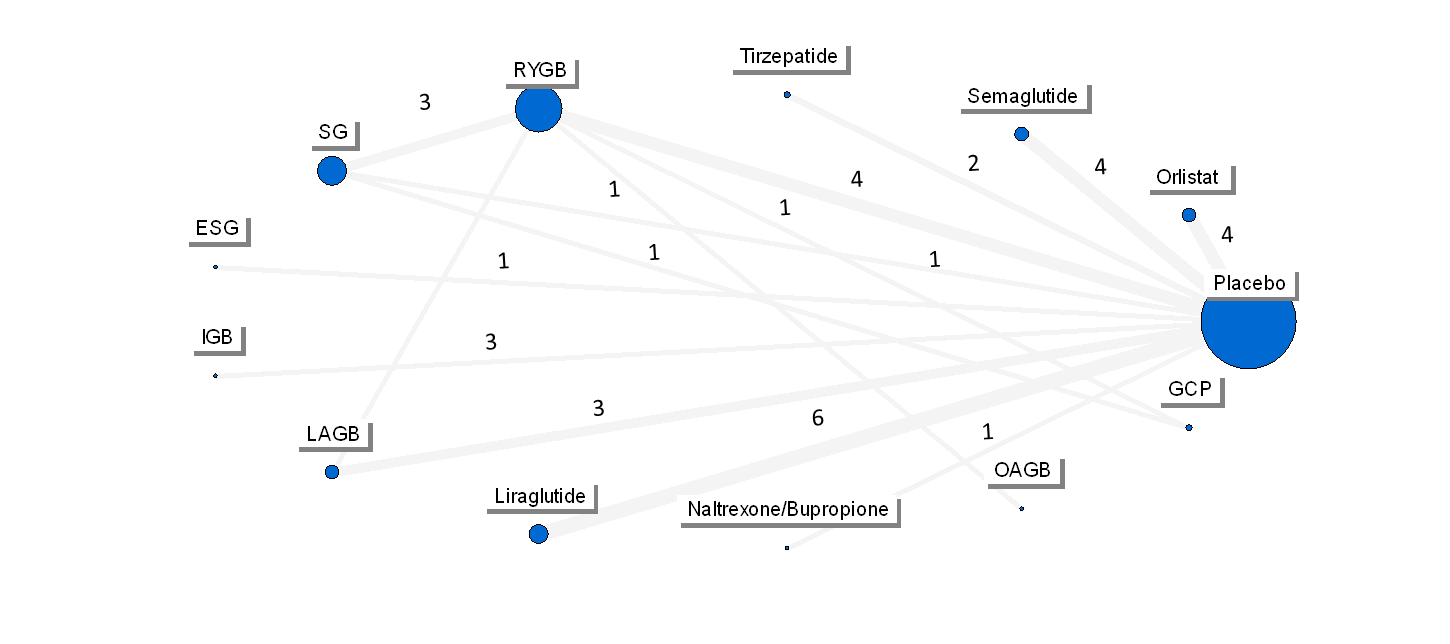


**B: overall**

**B1: in RCTs on patients with diabetes**

**C**

**
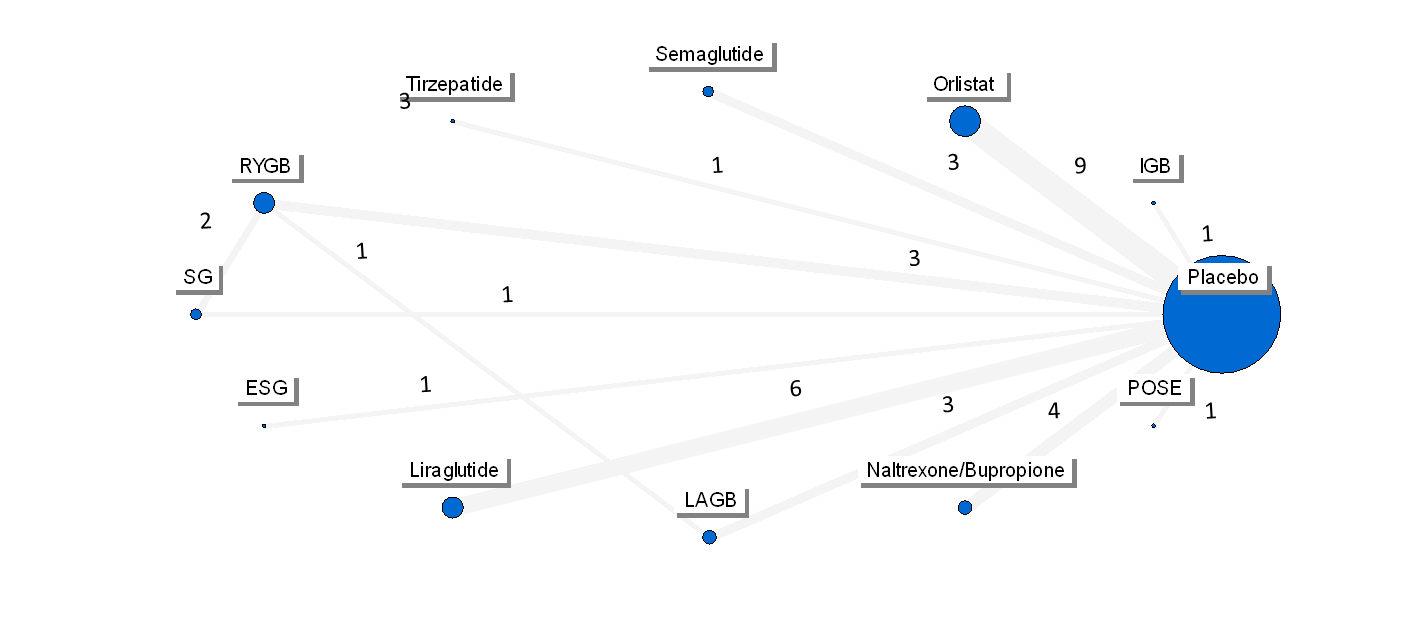
**

**D: overall**

**D1: in RCTs on patients with diabetes**

# Figure 26S – Effects of individual anti-obesity strategy on HbA1_c_ (and FPG at the endpoint (Panel A and C: network plots for HbA1c and FPG, respectively; Panel B and D: forest plots for HbA1c and FPG, respectively; Panel B1 and D1: forest plots for HbA1c and FPG in RCTs enrolling patients with diabetes, respectively) in studies with mean BMI at entry > 39.9 kg/m^2^.The node size  represents the quantity of entities or participants, while the edge (line) thickness indicates the strength or frequency of the connection between them, such as the number of studies or data points assessing the relationship

**A**

**B**

**B1**

**C**

**D**

**D1**

# Figure 27S – Effects of different treatments on endpoint total (Panel A; mg/dl), HDL-cholesterol (Panel B; mg/dl) , and triglycerides (Panel C; mg/dl) in Pbo/LSI/no therapy-controlled trials with mean BMI at entry ranging from 30 to 34,9 kg/m^2^. The node size represents the quantity of entities or participants, while the edge (line) thickness indicates the strength or frequency of the connection between them, such as the number of studies or data points assessing the relationship

**A**


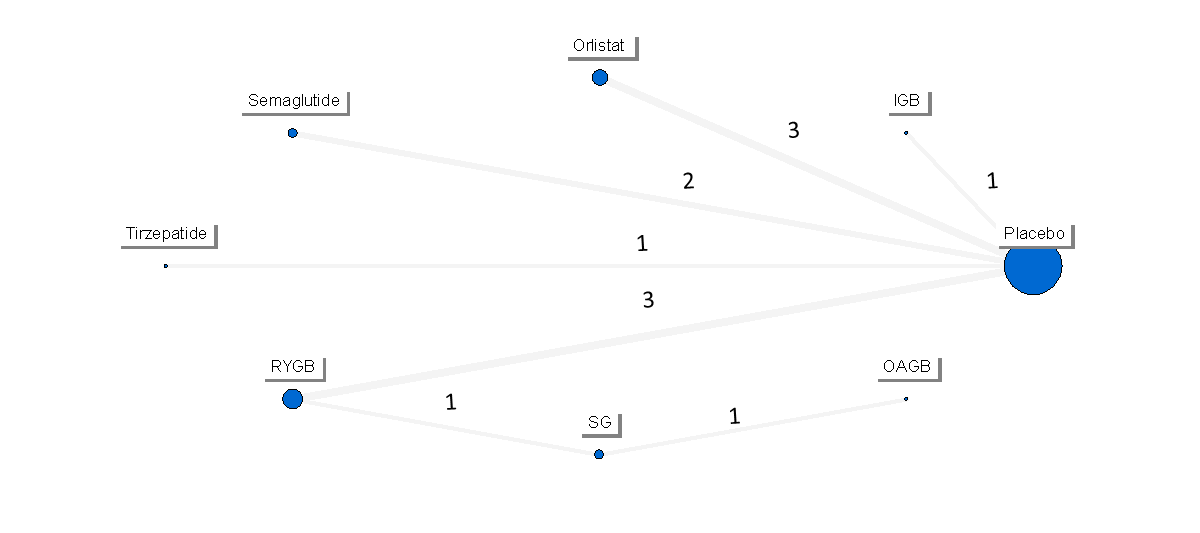

**B**


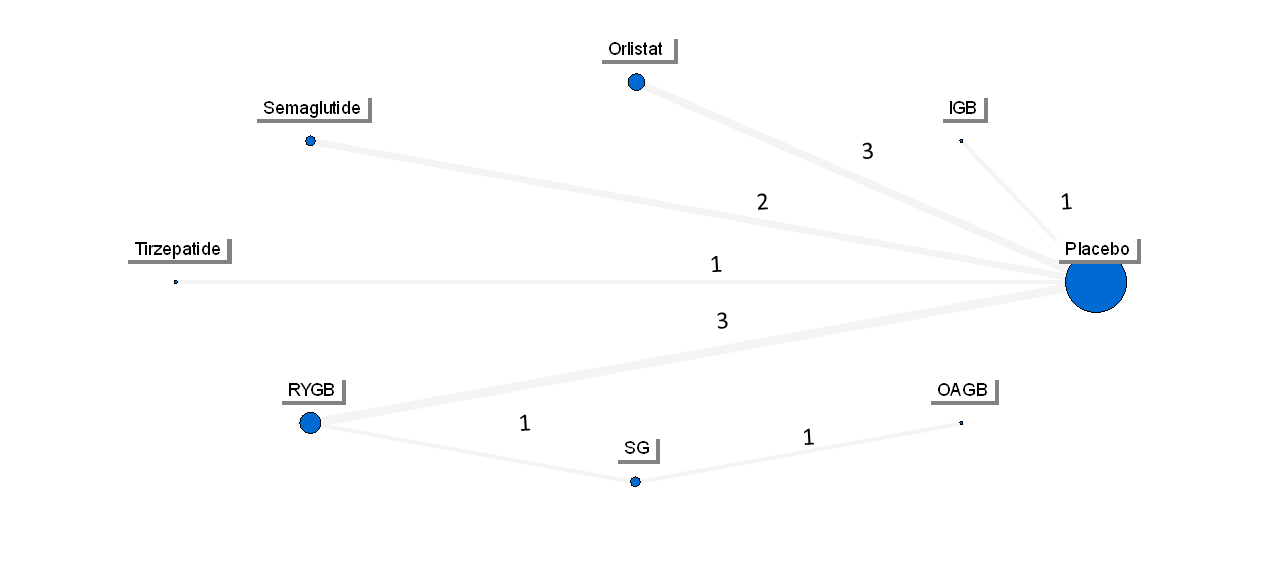

**C**


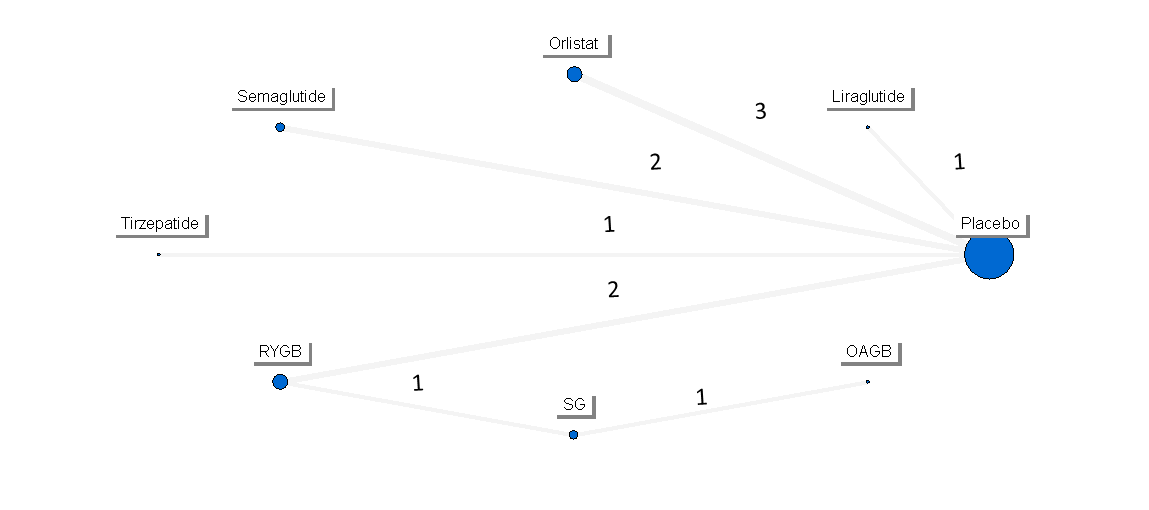

# Figure 28S – Effects of individual anti-obesity strategy on total cholesterol (Panel A: network plot and B: forest plot), HDL-cholesterol (Panel C: network plot and D: forest plot), and triglycerides (Panel E: network plot and F: forest plot) at the endpoint in studies with mean BMI at entry ranging from 35 to 39.9 kg/m^2^. The node size  represents the quantity of entities or participants, while the edge (line) thickness indicates the strength or frequency of the connection between them, such as the number of studies or data points assessing the relationship

**A**

**
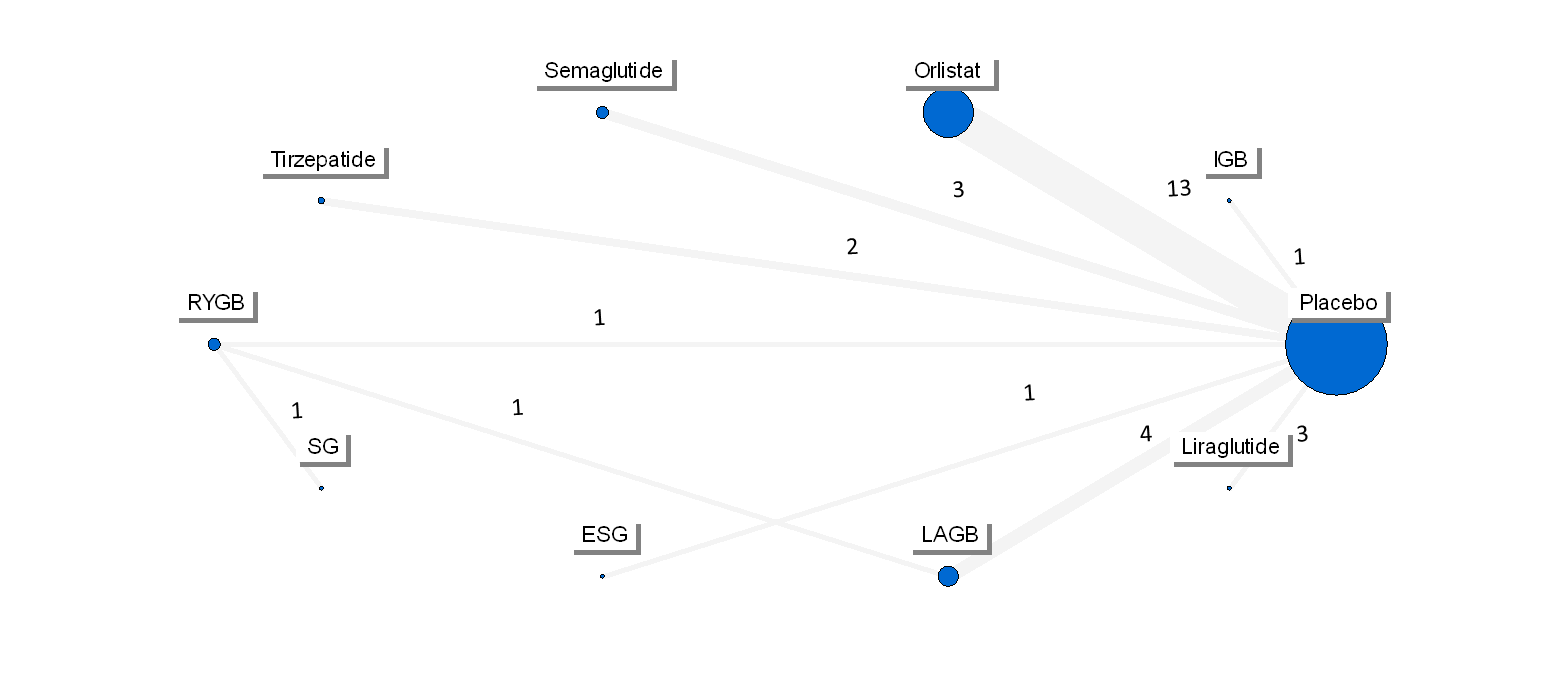
**

**B**

**C**

**
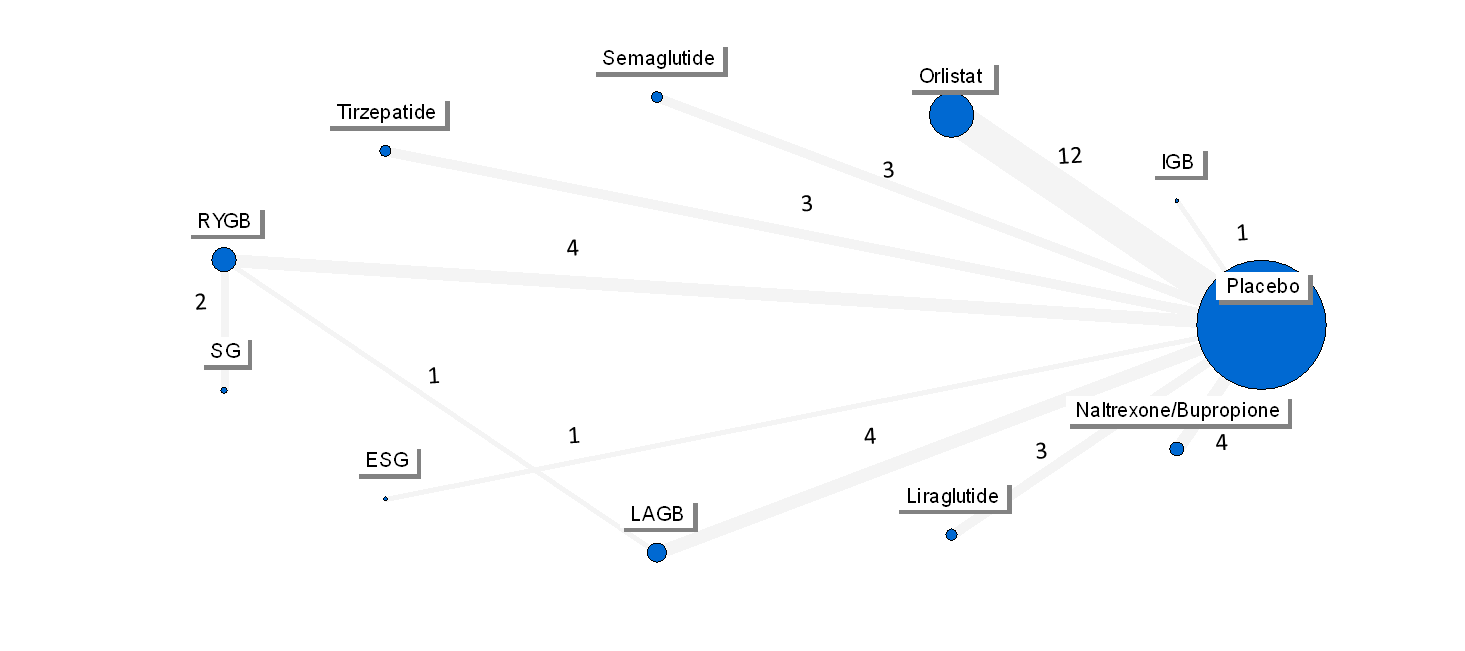
**

**D**

**E**

**
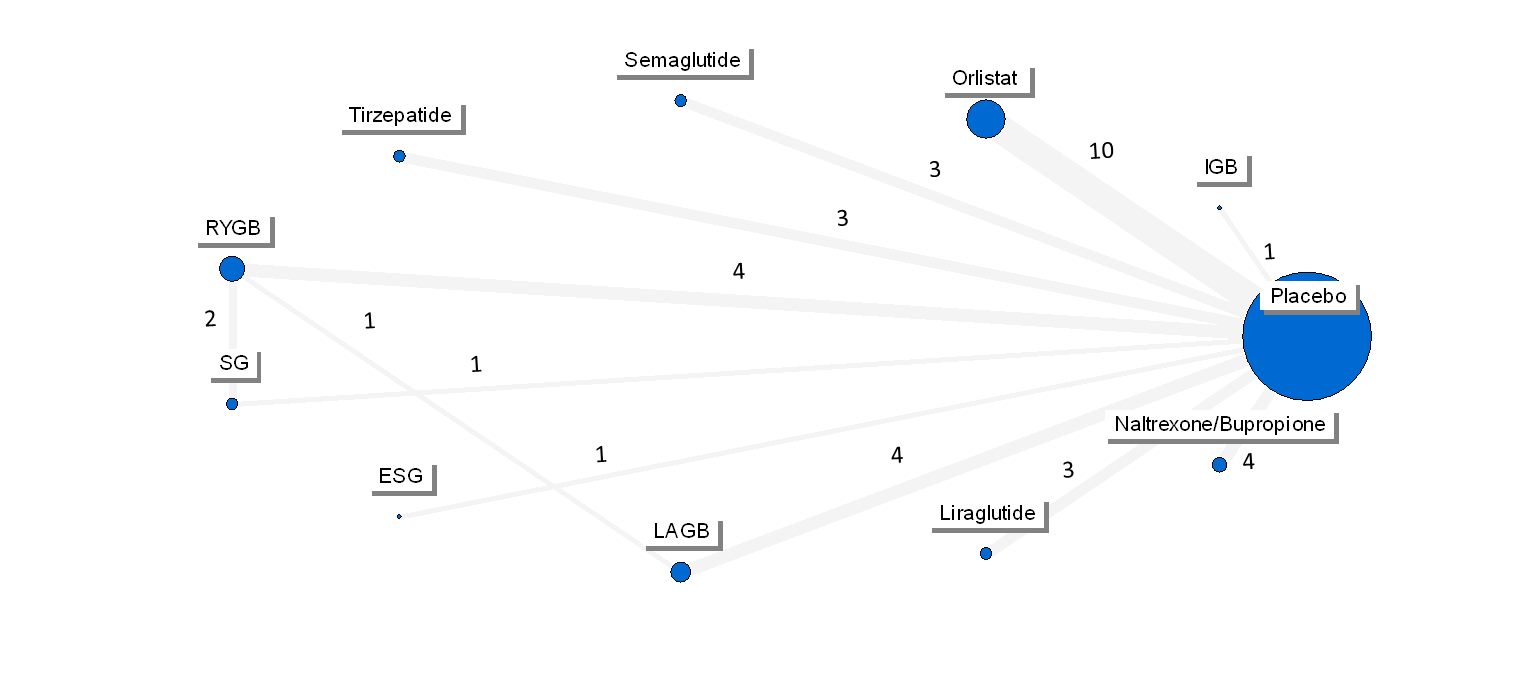
**

**F**

# Figure 29S – Effects of individual anti-obesity strategy on total cholesterol (Panel A: network plot and B: forest plot), HDL-cholesterol (Panel C: network plot and D: forest plot), and triglycerides (Panel E: network plot and F: forest plot) at the endpoint in studies with mean BMI at entry >39.9 kg/m^2^. The node size  represents the quantity of entities or participants, while the edge (line) thickness indicates the strength or frequency of the connection between them, such as the number of studies or data points assessing the relationship

**A**

**B**

**C**

**D**

**E**

**F**

# 30

# Figure 31S – Effects of individual anti-obesity strategy on systolic (Panel A: network plot and Panel B: forest plot) and diastolic blood pressure systolic (Panel C: network plot and Panel D: forest plot) in studies with mean BMI at entry >39.9 kg/m^2^. The node size  represents the quantity of entities or participants, while the edge (line) thickness indicates the strength or frequency of the connection between them, such as the number of studies or data points assessing the relationship

**A**

**B**

**C**

**D**

# Figure 32S – Effects of different treatments on OAMC remission (Panel A: MACE; Panel B: diabetes remission; Panel C: incident diabetes; Panel D: Hospitalization for heart failure; E: all-cause mortality) in Pbo/LSI/no therapy-controlled trials with mean BMI at entry ranging from 30 to 34.9 kg/m2.

**A**


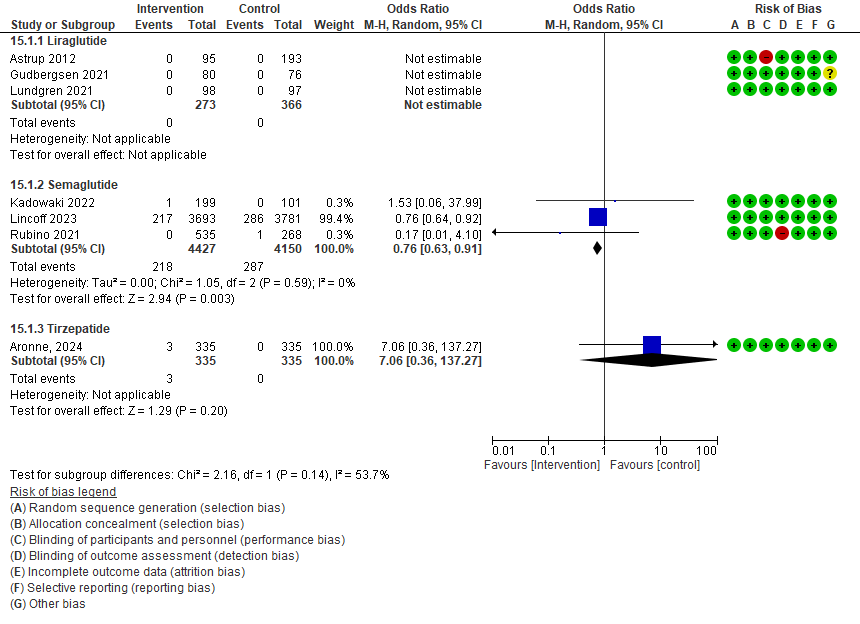


**B**

**
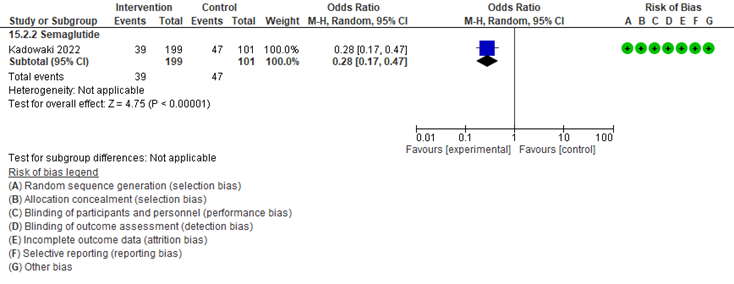
**

**C**

**
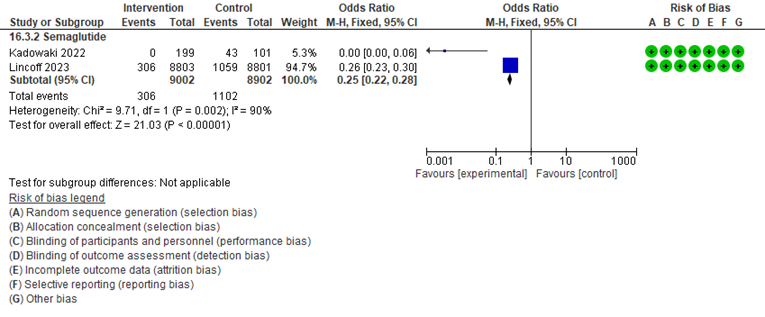
**

**D**


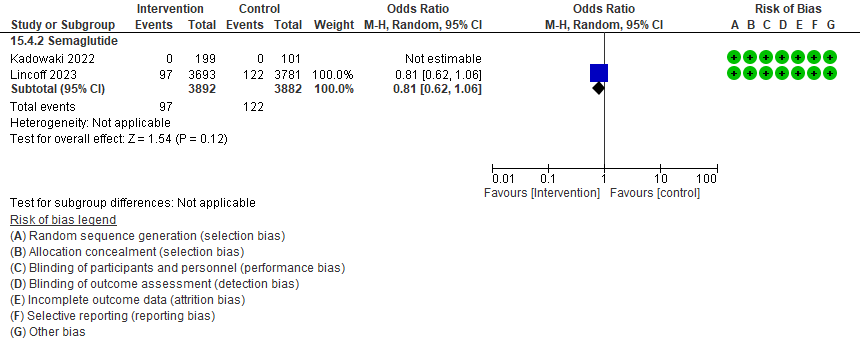


**E**

# Figure 33S – Effects of different treatments on OAMC remission (Panel A: MACE; Panel B and C: diabetes remission; Panel D and E: hypertension remission; Panel F and G: dyslipidemia remission; Panel H: incident diabetes; Panel I: Hospitalization for heart failure; Panel L: Liver fibrosis (reduction of at least one stage); Panel M: MASH remission with no worsening of fibrosis; Panel N: OSAS remission: Apnea-Ipopnea Index< 5 events/hour) in Pbo/LSI/no therapy-controlled trials with mean BMI at entry ranging from 35 to 39.9 kg/m^2^. The node size  represents the quantity of entities or participants, while the edge (line) thickness indicates the strength or frequency of the connection between them, such as the number of studies or data points assessing the relationship

**A**

**
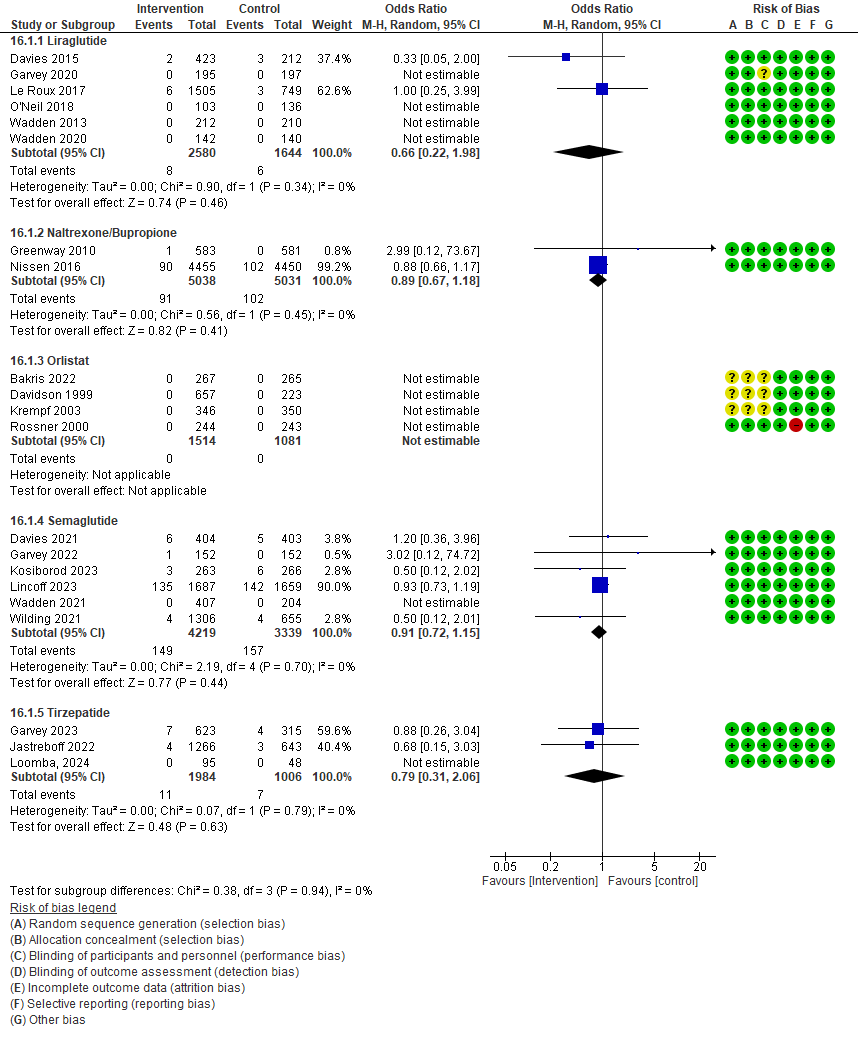
**

**B**

**
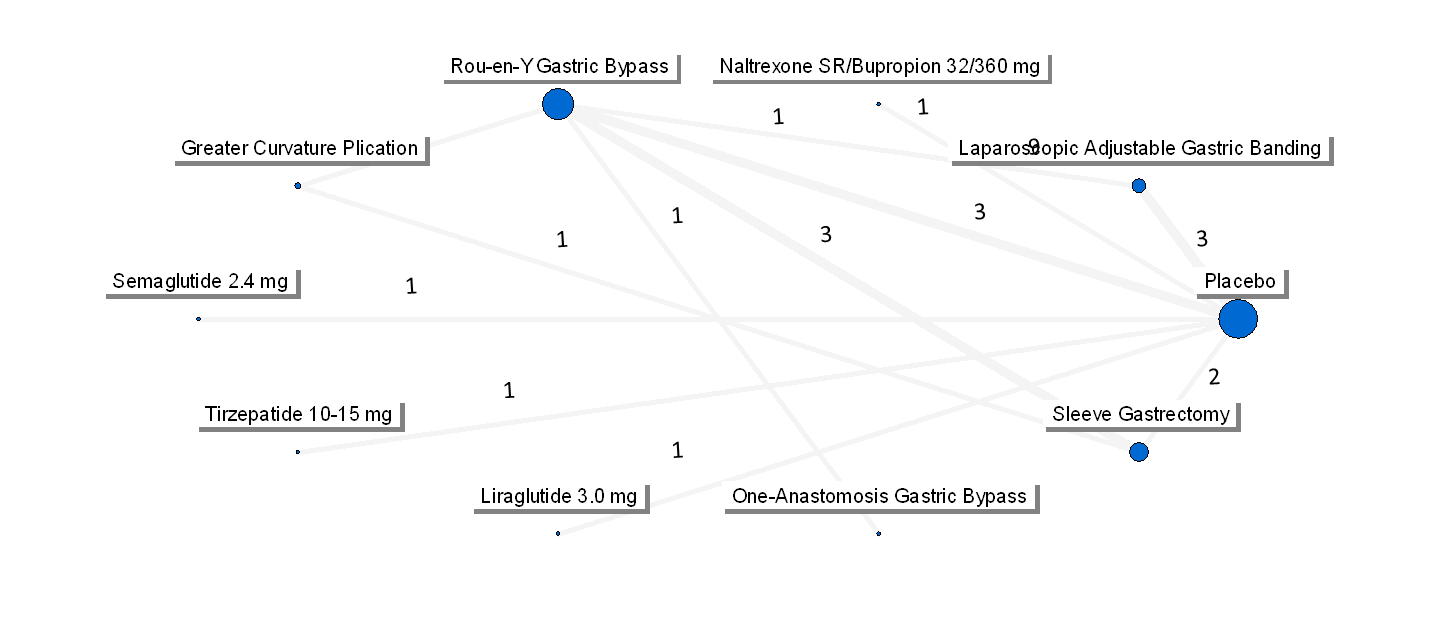
**

**C**

**D**

**
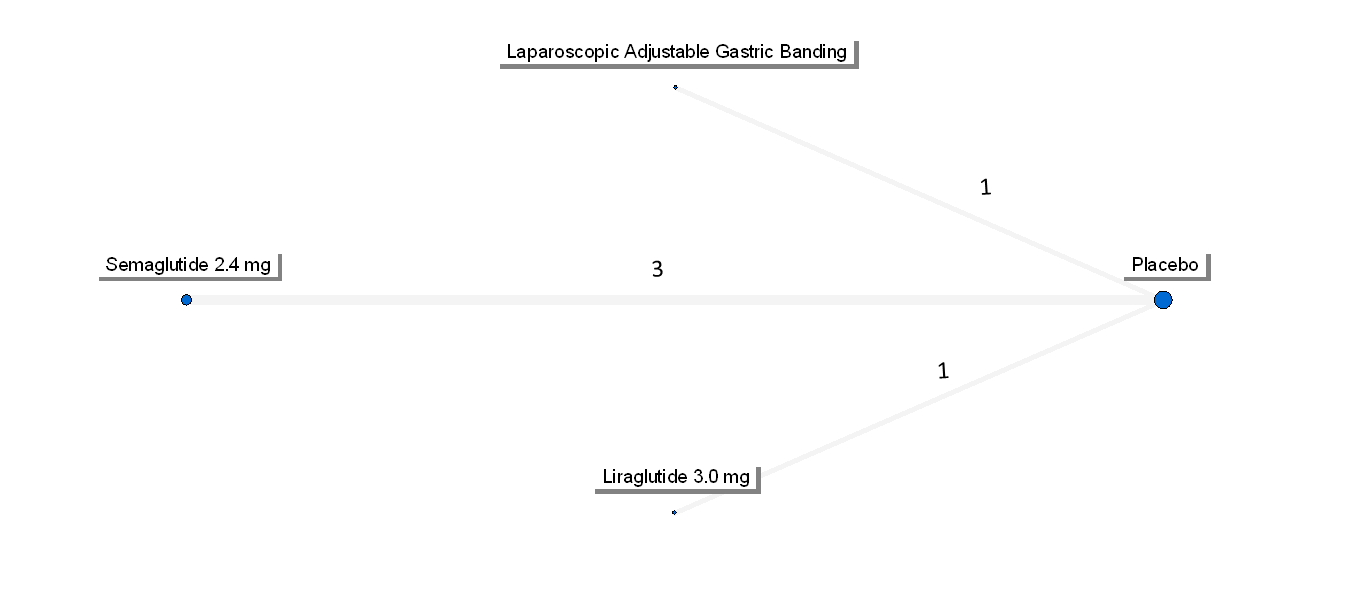
**

**E**

**F**

**
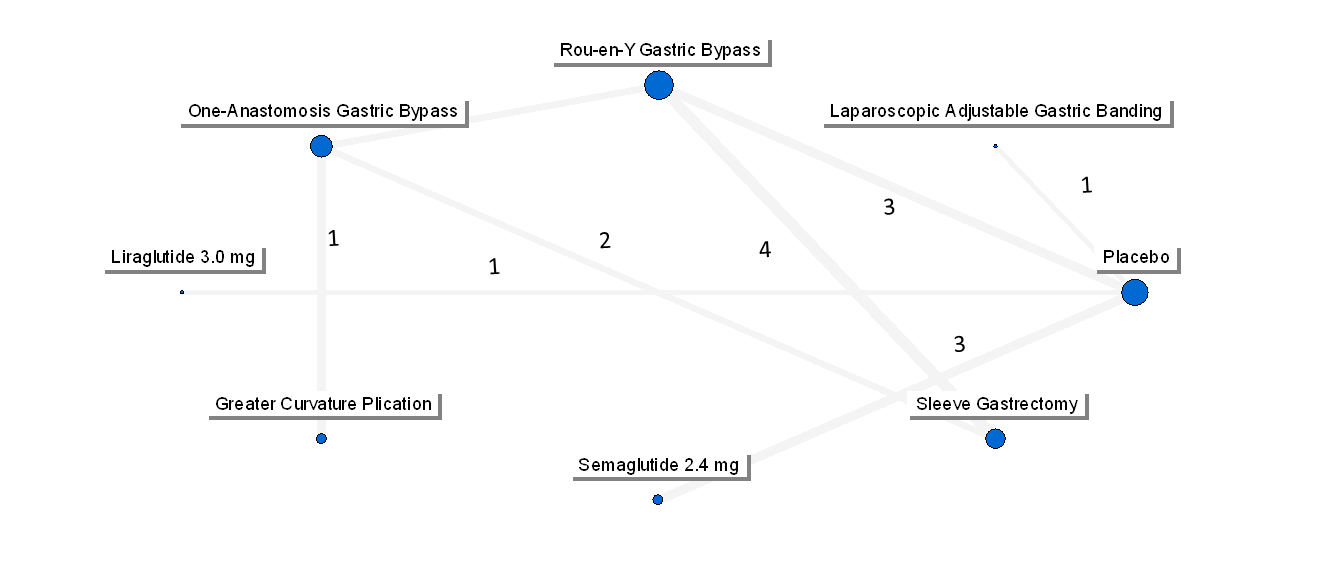
**

**G**

**H**


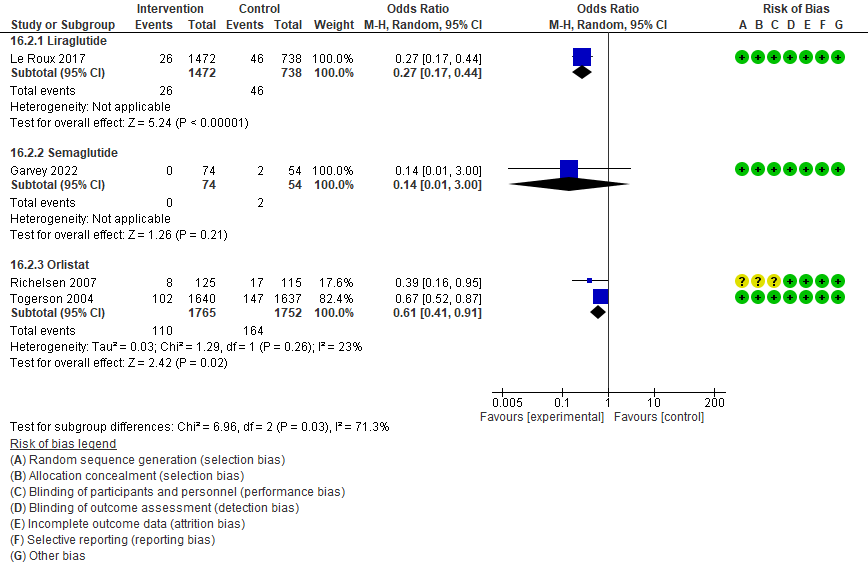


I


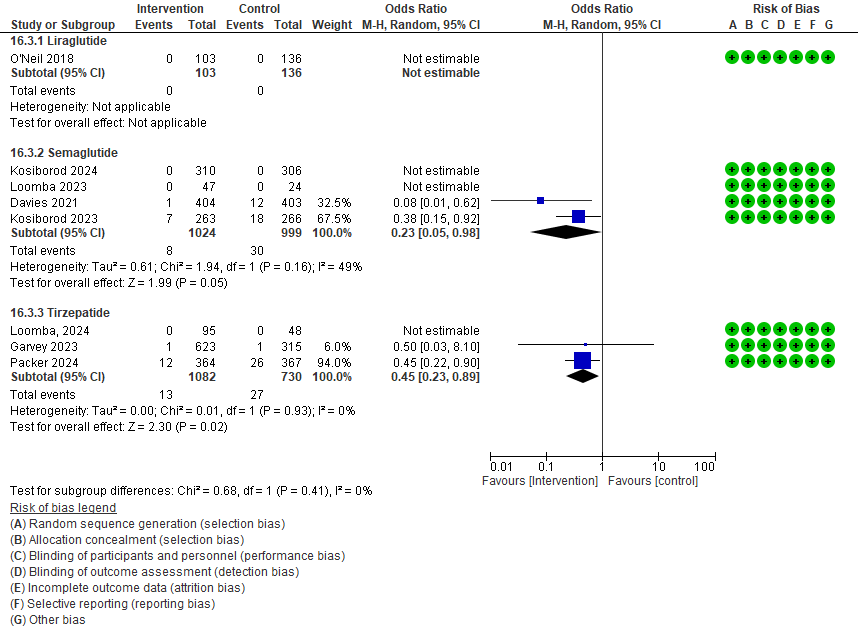


**L**

**
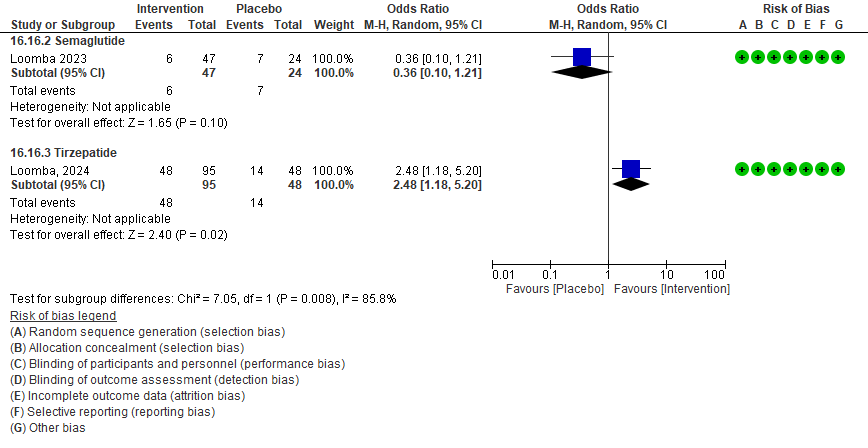
**

**M**


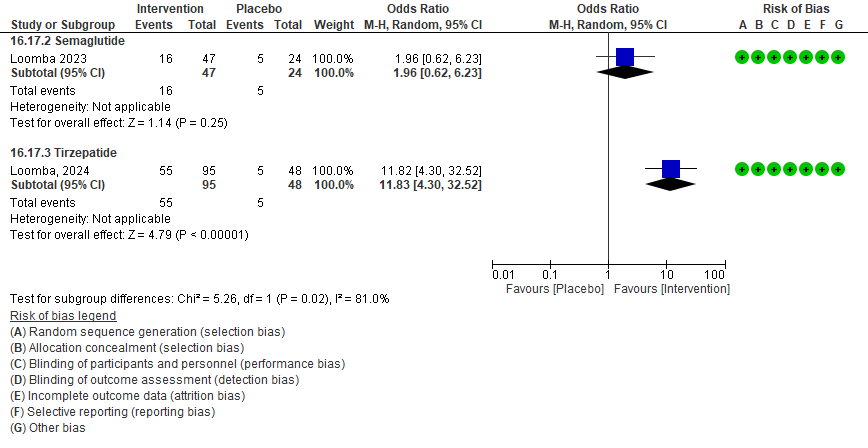


**N**

**
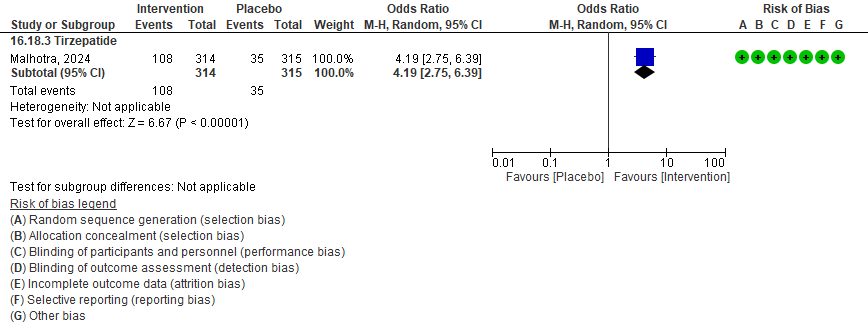
**

# Figure 34S – Effects of different treatments on OAMC remission (Panel A and B; diabetes remission; Panel C and D: incident diabetes; Panel E: hypertension remission; Panel F: dyslipidemia remission; Panel G: osteoarthritis remission; Panel H: OSAS remission: Apnea-Ipopnea Index< 5 events/hour) in Pbo/LSI/no therapy-controlled trials with mean BMI at entry >39.9 kg/m^2^.

**A:** versus LSI/Pbo/No therapy

**
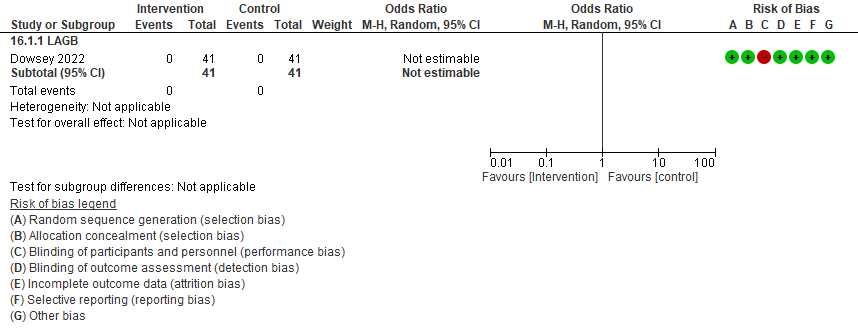
**

**B:** head-to-head comparisons

**OAGB**

**
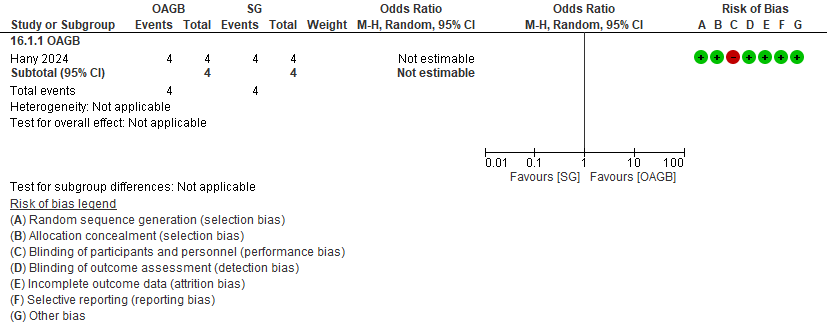
**

**RYGB**

**
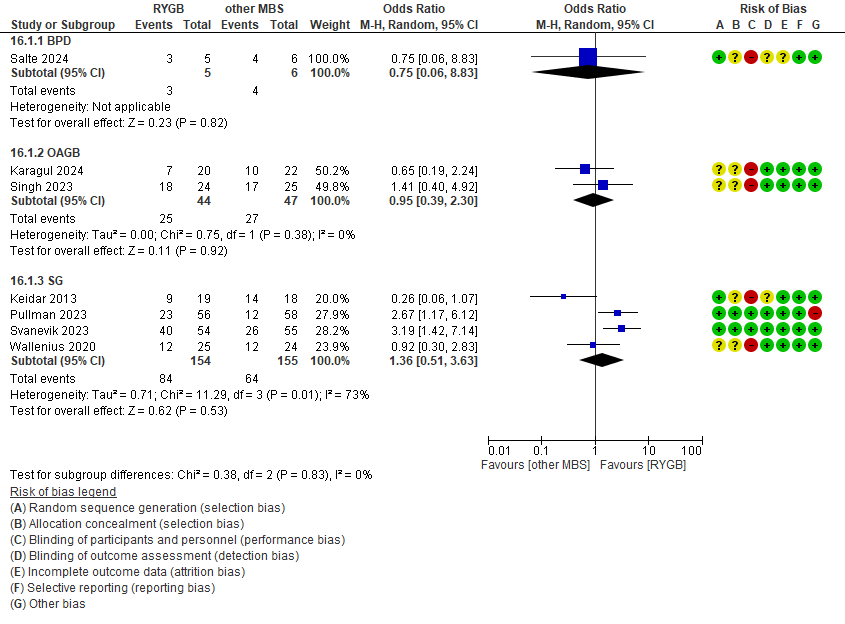
**

**C:** versus LSI/Pbo/No therapy

**
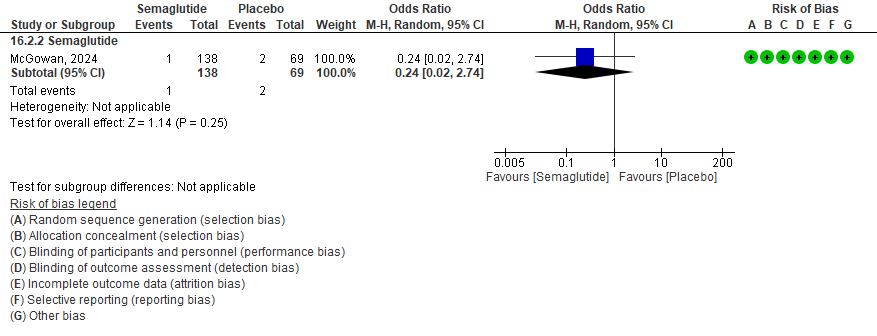
**

**D:** head-to-head comparisons

**
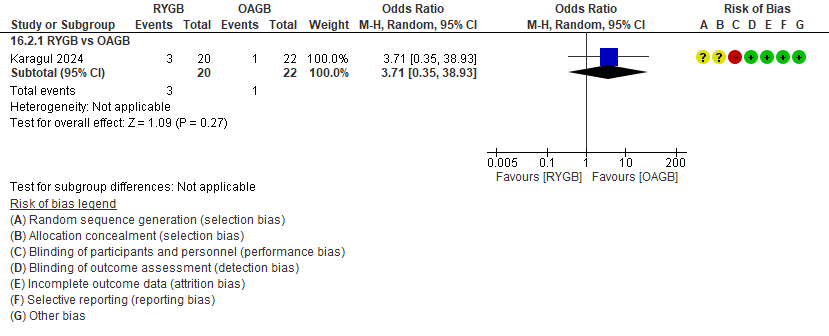
**

**E:** head-to-head comparisons

**
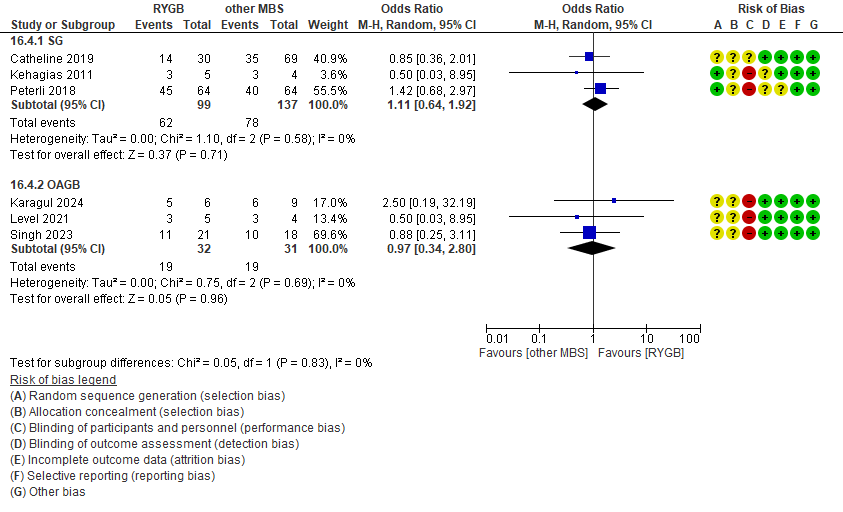
**

**F:**head-to-head comparisons

**RYGB**

**
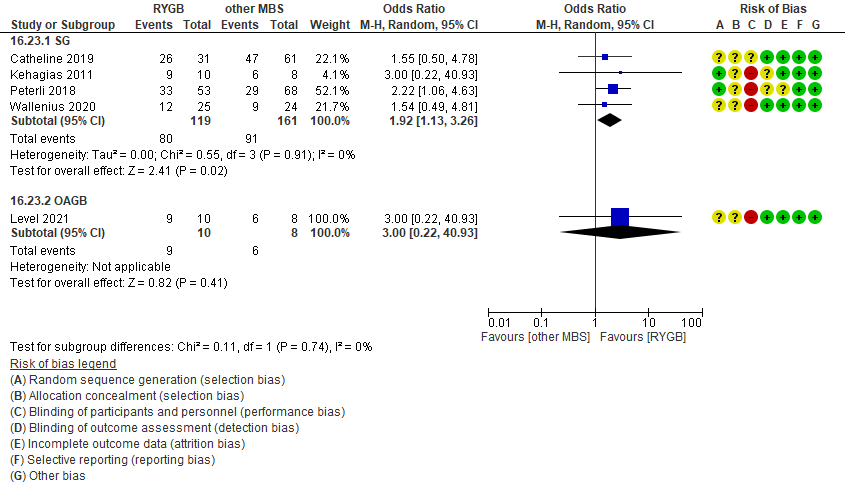
**

**G:** head-to-head comparisons


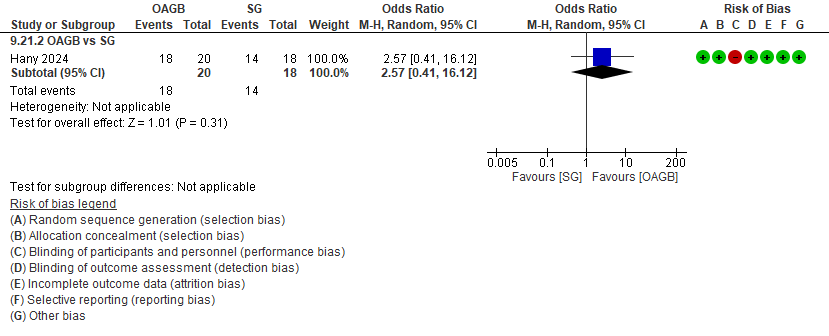


**H**

**RYGB vs:**


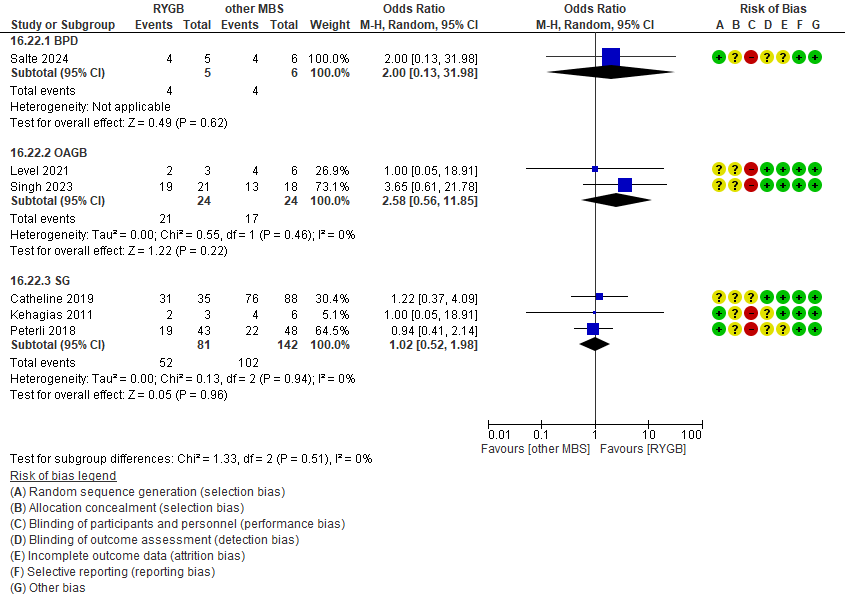


**OAG vs SG**


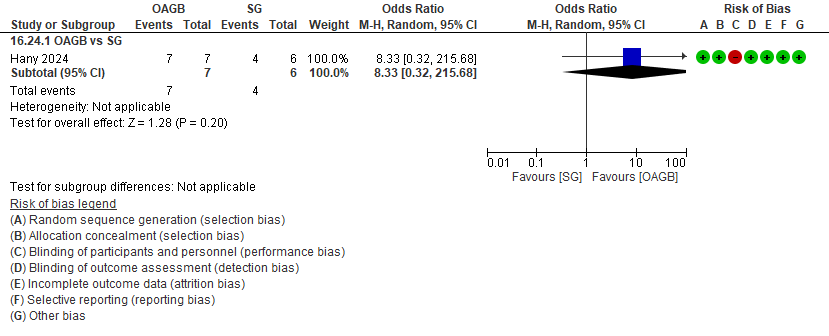


# Figure 35S – Effects of different anti-obesity strategies on surgical (Panel A: versus LSI/Pbo/No therapy; Panel B: head-to-head comparisons) and overall SAE (Panel C: versus LSI/Pbo/No therapy) at the endpoint in RCTs with a mean BMI at entry 30-34.9 Kg/m2. SG: Sleeve Gastrectomy; OAGB: One-anastomosis gastric bypass; RYGB: Roux-en-Y Gastric By-Pass.

**A**

**
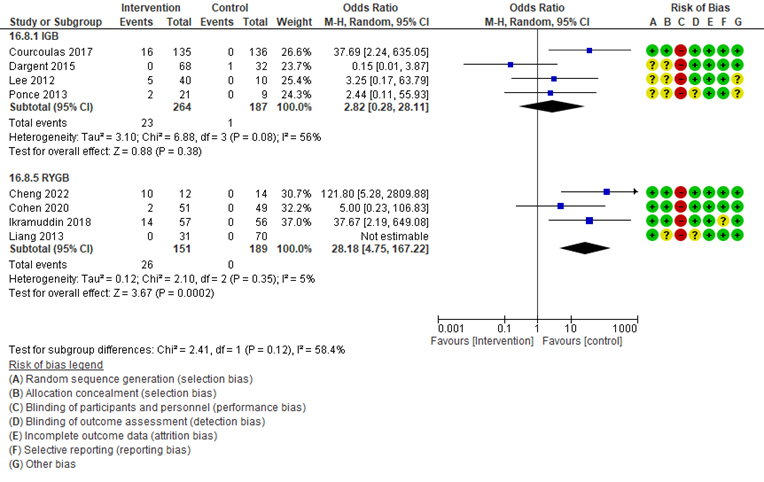
**

**B**

**
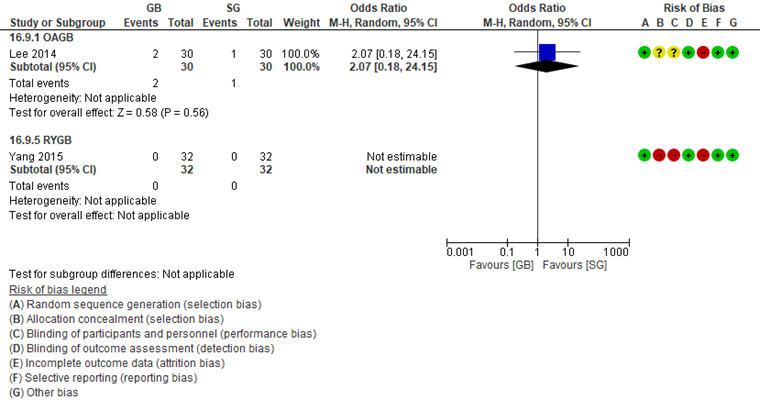
**

**C**

**
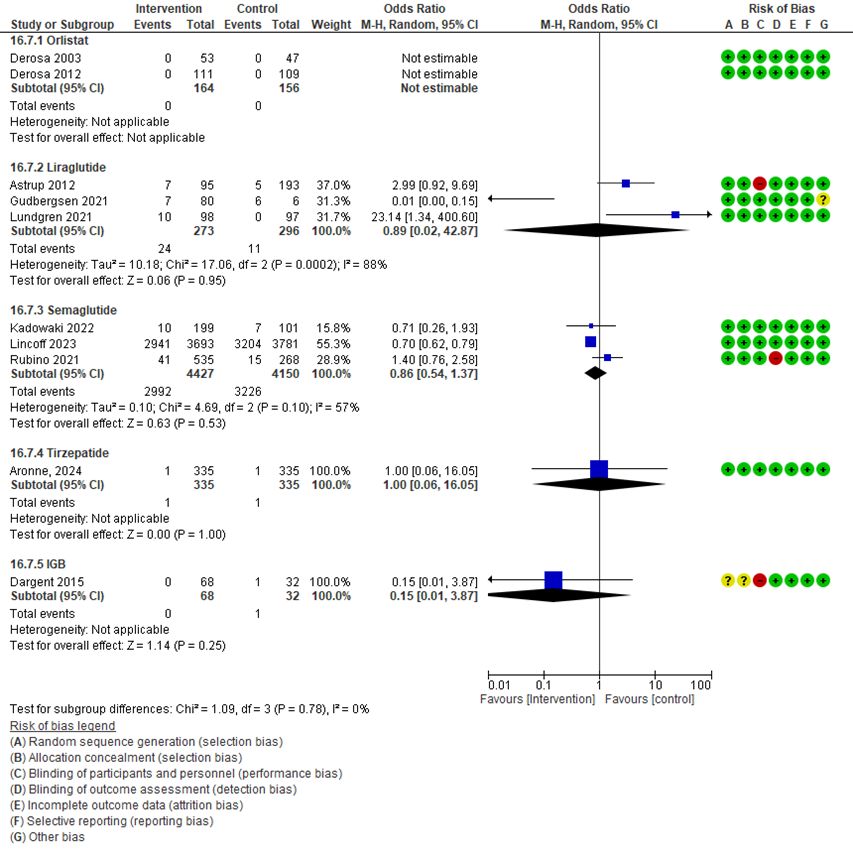
**

# Figure 36S – Effects of different treatments on surgical SAE (Panel A: versus LSI/Pbo/No therapy; Panel B: head-to-head comparisons) and overall SAE (Panel C: network plot; Panel D: forest plot) in trials with mean BMI at entry ranging from 35 to 39.9 kg/m^2^. The node size  represents the quantity of entities or participants, while the edge (line) thickness indicates the strength or frequency of the connection between them, such as the number of studies or data points assessing the relationship

**A**

**
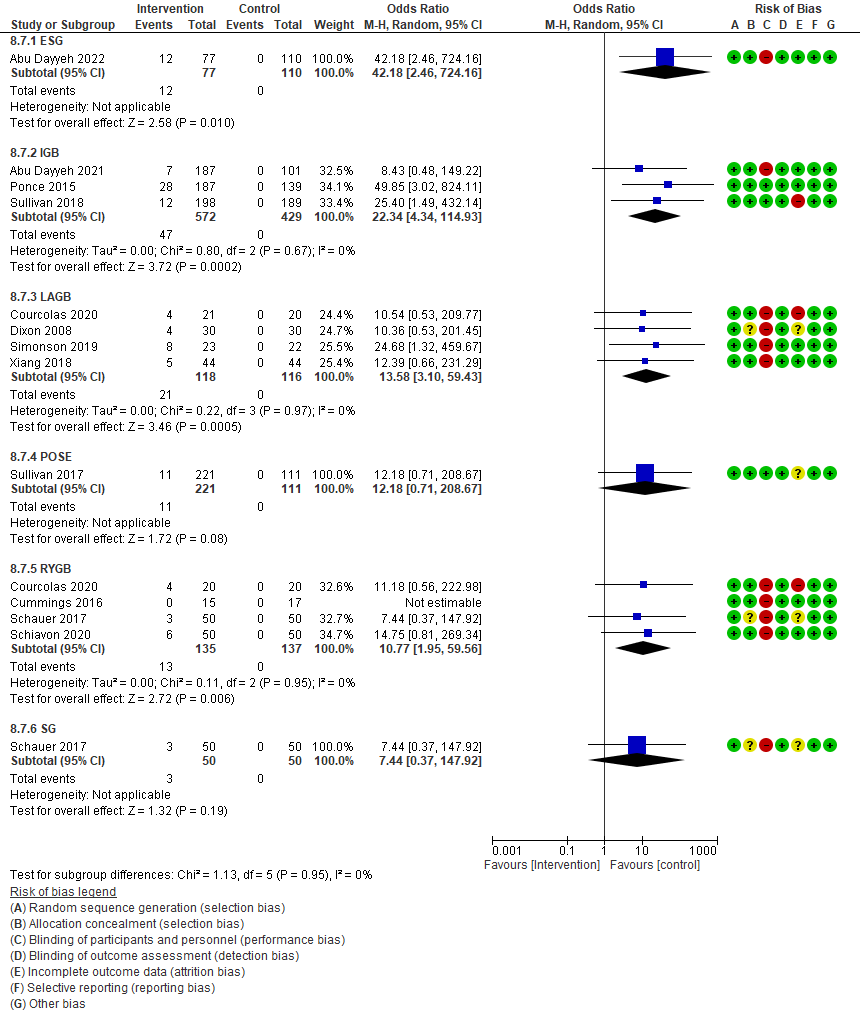
**

**B:**

**RYGB vs other MBS**

**
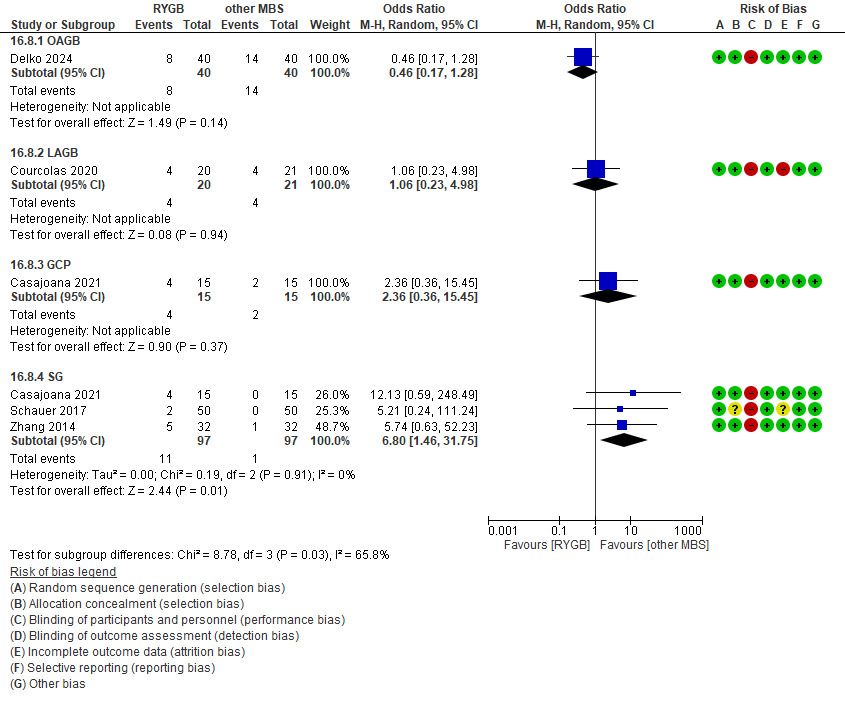
**

**SG vs GCP**


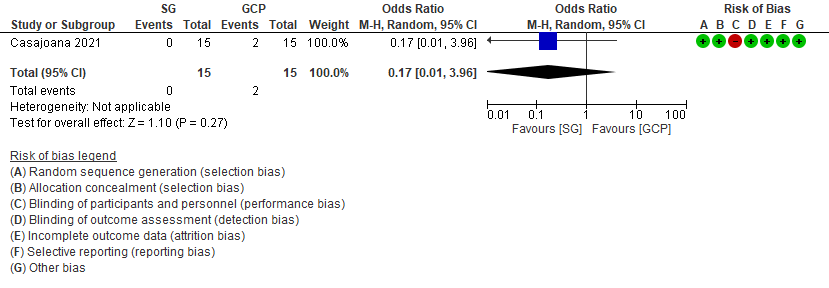


**C**

**D**

# Figure 37S – Effects of different treatments on overall SAE (Panel A: versus LSI/Pbo/No therapy; Panel B: head-to-head comparisons), surgical SAE (Panel C: network plot; Panel D: forest plot), and all-cause mortality (Panel E and F), in trials with mean BMI at entry > 39.9 kg/m^2^. The node size  represents the quantity of entities or participants, while the edge (line) thickness indicates the strength or frequency of the connection between them, such as the number of studies or data points assessing the relationship

**A**

**B**

**C**

**D**

**E**

**F**

# Figure 38S – Effects of different treatments on all-cause mortality (Panel A: network plot; Panel B: forest plot) in trials with mean BMI at entry ranging from 35 to 39.9 kg/m^2^. The node size  represents the quantity of entities or participants, while the edge (line) thickness indicates the strength or frequency of the connection between them, such as the number of studies or data points assessing the relationship

**A**

**B**

# Figure 39S – Effects of different treatments on quality of life at the endpoint (Panel A: IWQOL Lite; Panel B: SF-36 General Helath; Panel C: SF-36 Physical Functioning) in trials with mean BMI at entry ranging from 35 to 39.9 kg/m^2^.

**A**

**
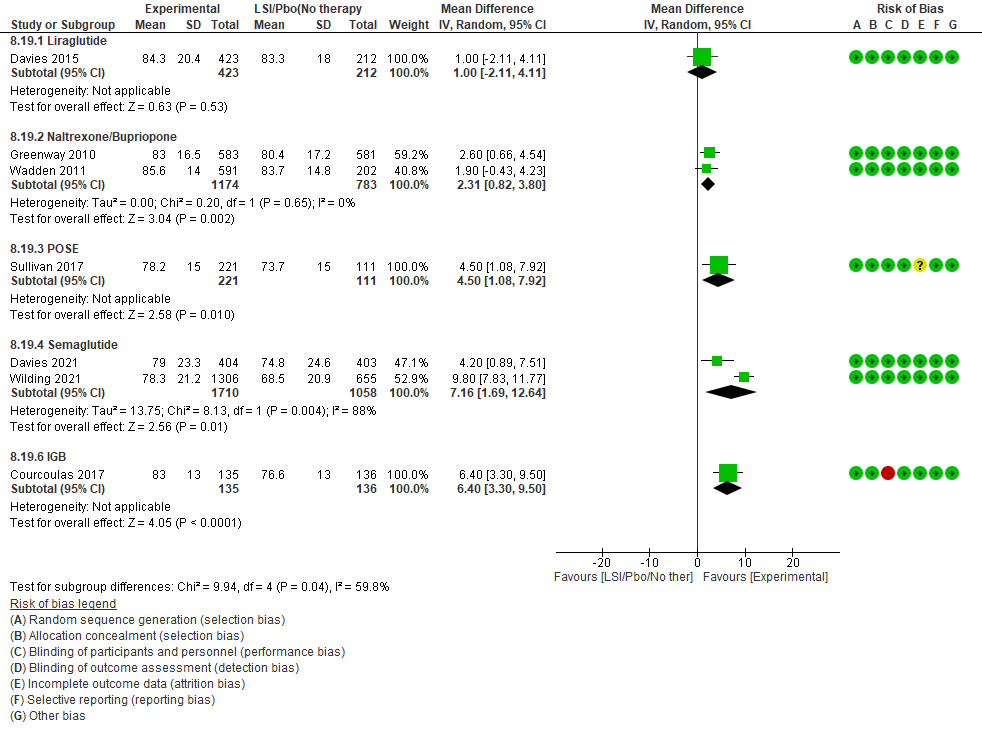
**

**B**

**
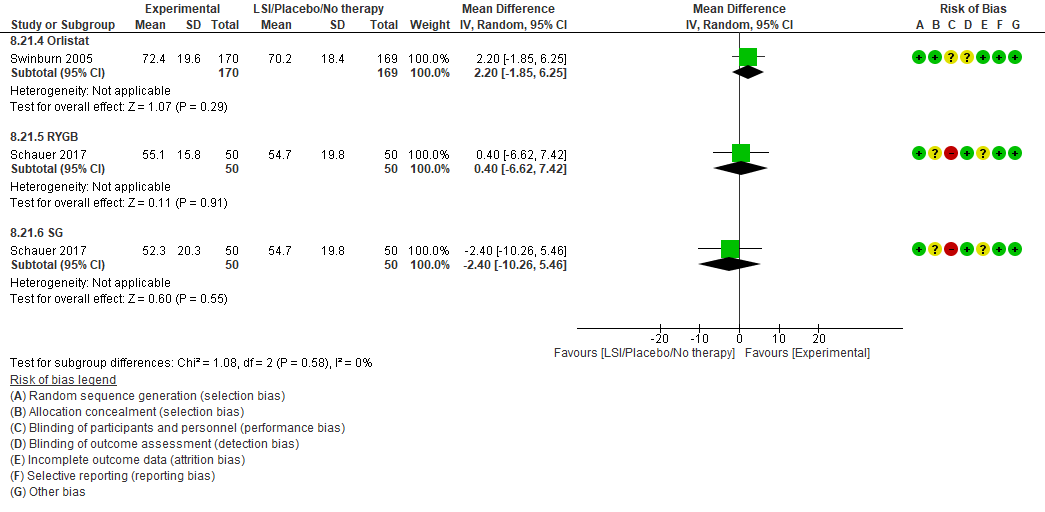
**

**C**

**
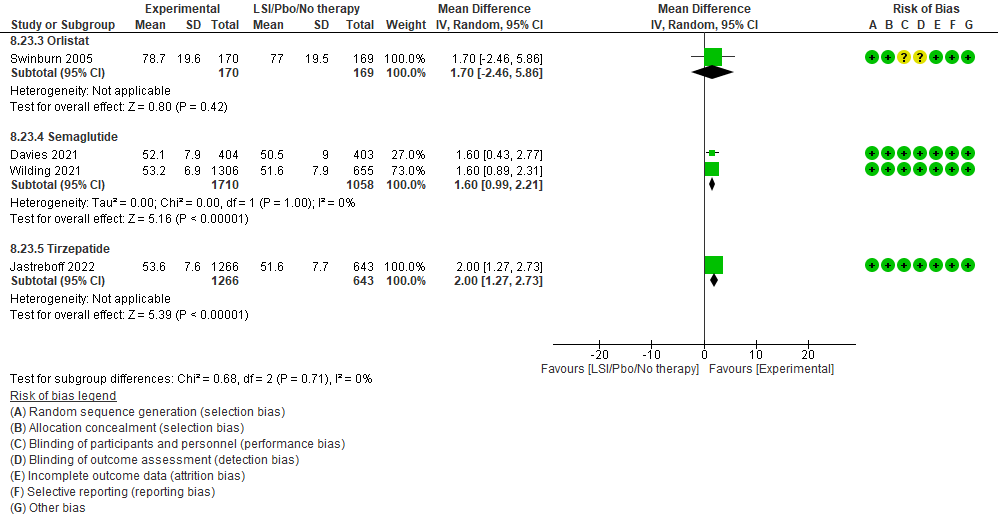
**

# Figure 40 – Overall NMA risk of bias (within-study and reporting bias, indirectness, imprecision, heterogeneity, and incoherence) for each comparison, including studies with a mean BMI at entry ranging from 30 to 34.9 kg/m2 (Panel A), 35 to 39.9 kg/m2 (Panel B), and >39.9 kg/m2 (Panel C) versus the reference category (i.e., LSI/Placebo/None).

**A**


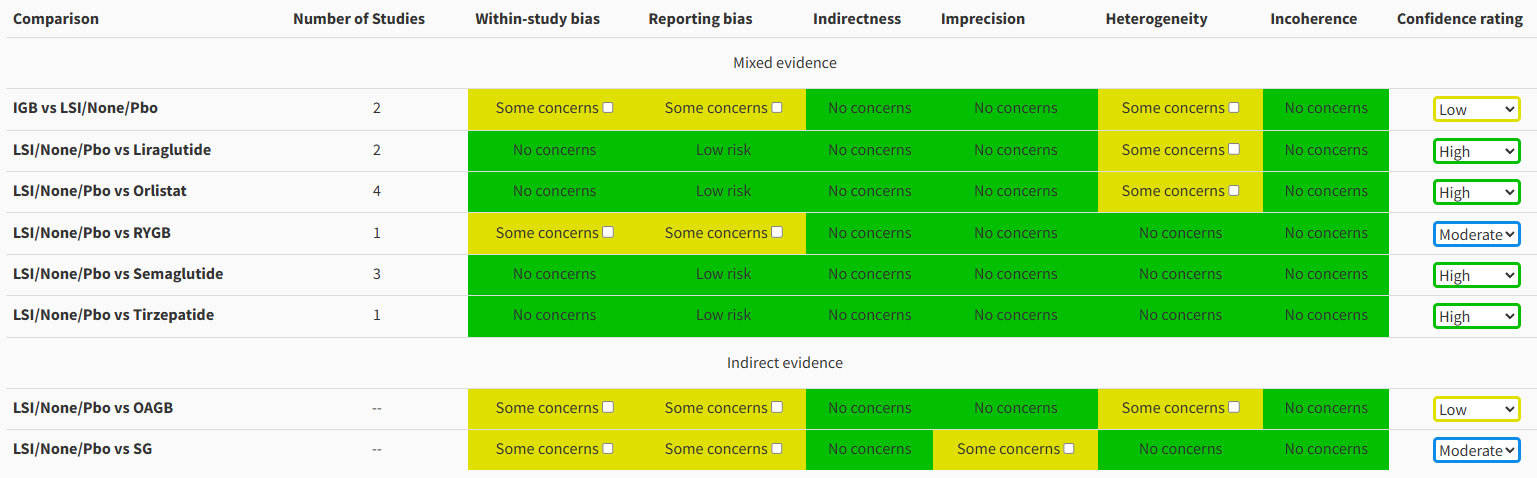


# **B**


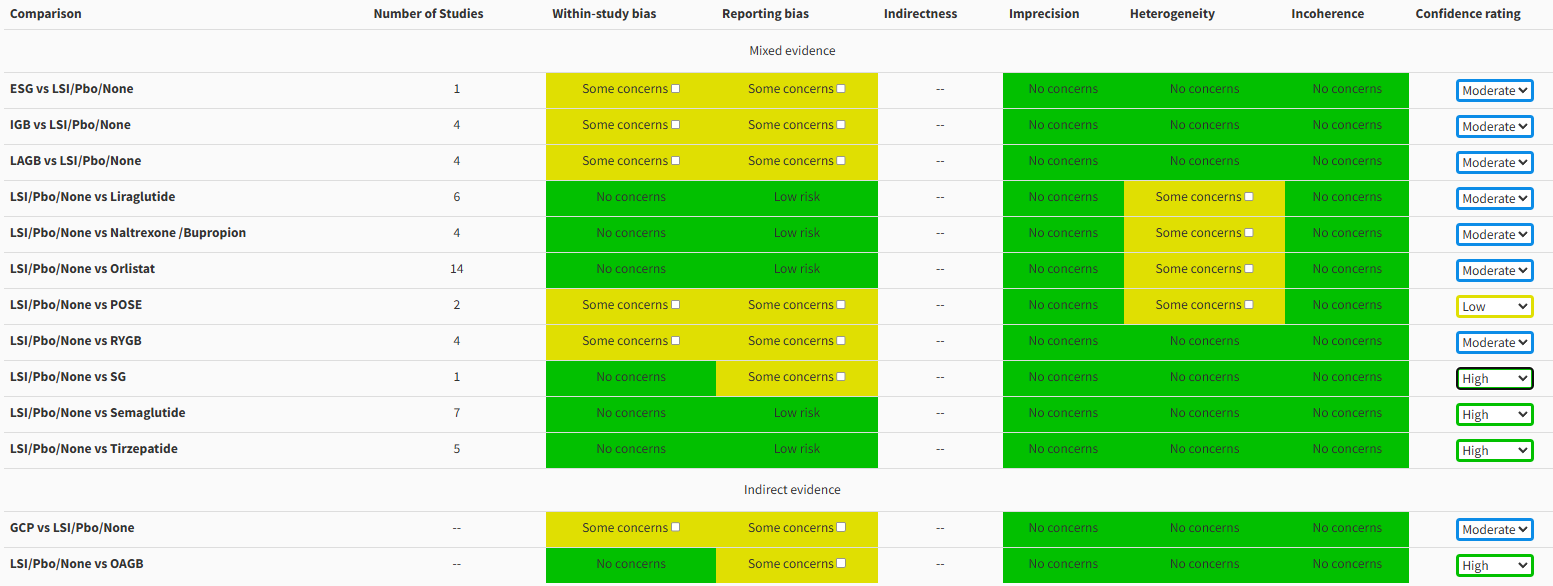


**C**


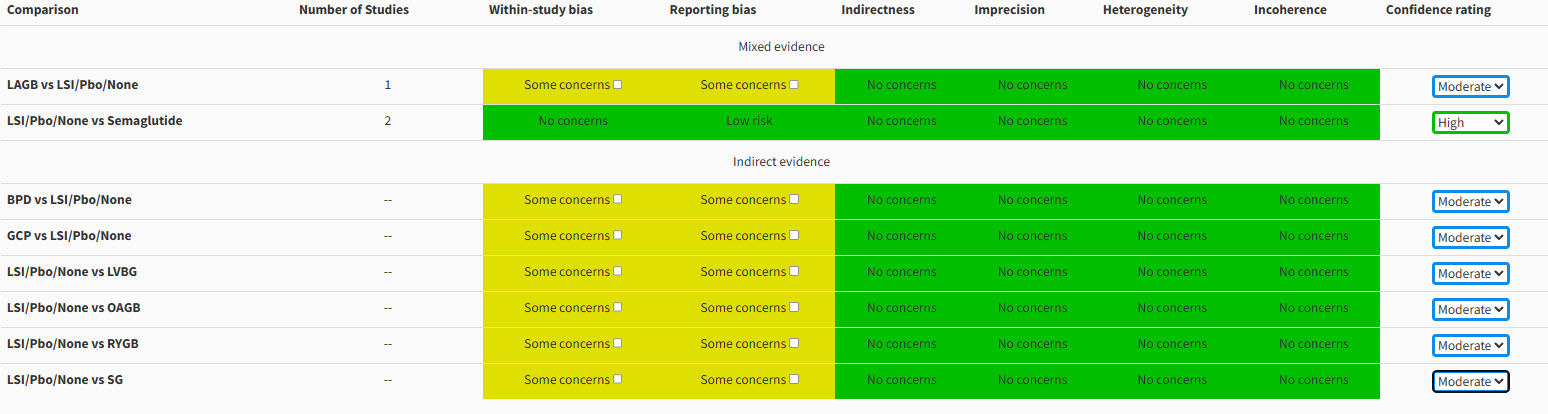


# TABLES

# Table 1S – PRISMA checklist.

| **Section/topic** | **#** | **Checklist item** | **Reported on page #** |
| --- | --- | --- | --- |
| **TITLE** | | |  |
| Title | 1 | Obesity in adults: efficacy and safety of 2024-EMA- and FDA-approved surgical, endoscopic, and pharmacological treatments. A comprehensive systematic review and network meta-analysis of randomized controlled trials. | - |
| **ABSTRACT** | | |  |
| Structured summary | 2 | Background: To compare different anti-obesity strategies (Obesity Management Medications – OMM, Endoscopic Bariatric Procedures – EBP, and Metabolic Bariatric Surgery – MBS) with Lifestyle Intervention/Placebo/No therapy (LSI/Pbo/NT) for the treatment of obesity. Methods: This systematic review and Network Meta-Analysis (NMA) included randomized clinical trials (RCTs) comparing OMM, EBP, and MBS versus either LSI/Pbo/NT or active comparators in subjects affected by obesity. A Medline and Embase search was performed up to 1st December 2024 for RCTs on approved weight-lowering interventions in adults with obesity. The primary endpoint was Total Body Weight Loss (%; TBWL%) analyzed at different time points: 26-52, 53-104, 105-156, and >156 weeks. Secondary endpoints included all-cause mortality, quality of life, and serious adverse events (SAE). Weighted Mean Difference and 95% Confidence Intervals (WMD, 95% CI) for continuous variables, and Mantel-Haenszel Odds Ratio (MH-OR, 95% CI) for categorical variables were calculated, using random effect models. The study was registered with PROSPERO, CRD42024625338.  Findings: Out of 139 trials, 54, 21, and 64 trials were performed on MBS, EP, and OMMs, enrolling 61,961, 2,934, and 5,991, respectively. At 26-52 weeks (N=104 comparisons, with no evidence of inconsistency; H=1.07), all treatments showed a significant effect versus reference (LSI/None/Placebo), except Primary Obesity Surgery, Endoluminal (POSE) and LAGB (Laparoscopic Adjustable Gastric Banded); the estimated TBWL was greater than 10% for most surgical procedures and tirzepatide. Only a limited number of comparisons was available at 53-104 weeks (N=42), 105-156 weeks (N=28), and 156-520 weeks (N=26). No long-term data was available for EBP and for most OMM (except orlistat). For the majority of the assessed treatments, the estimated longer-term efficacy was similar to that at 26-52 weeks, with the notable exception of LAGB and GCP, the effects of which seem to fade after 104 weeks. EBP and MBS were generally associated with a greater risk of SAE than OMM. In the long term, Bilio-Pancreatic Diversion) appeared to produce a greater incidence of SAE than other types of MBS.  Interpretation: the results obtained are in our opinion of interest for clinicians involved in the management of obesity. For the first time, different approaches have been compared in different categories of patients, giving clearer overall picture of their effectiveness. EBP, with exception of ESG, appear to be manifestly inferior to the most commonly used OMM (i.e., semaglutide and tirzepatide), which seem to be competitive even in candidates for MBS. Some surgical interventions seem to be outdated (i.e. LAGB, and GCP) or falsely considered more effective (BPD) than others and therefore they should be abandoned. | 1 |
| **INTRODUCTION** | | |  |
| Rationale | 3 | The development of pharmacological, endoscopic, and surgical research has led to the discovery of novel treatments in recent years, providing clinicians with a relatively wide range of therapeutic options. However, the availability of randomized controlled trials (RCTs) directly comparing different OMMs, other than LSIs, remains limited. In particular, there are very few studies comparing OMMs with MBS. A Network Meta-Analysis (NMA), providing indirect comparisons of efficacy and safety, can therefore be of help in guiding physicians’ choices. | 3 |
| Objectives | 4 | The present network meta-analysis is aimed the assessment of the effect of OMM, EBP, and MBS in subjects affected by obesity with respect to body weight loss, mortality, SAE, and quality of life. | 3 |
| **METHODS** | | |  |
| Protocol and registration | 5 | The study has been uploaded on the PROSPERO website (#CRD42024625338). | 4 |
| Eligibility criteria | 6 | All studies were included if they satisfied the following criteria: (i) randomized trials, (ii) duration ≥52 weeks for OMM and MBS and ≥26 weeks for EBP. (iii) on obesity and (iv) comparing antiobesity strategies versus LSI/Pbo/NT, or comparing two different strategies. | 4 |
| Information sources | 7 | A MEDLINE and embase database search were performed to identify all clinical trials (English only) up to December 1^st^, 2024. | 4 |
| Search | 8 | See Table 2S. | 4 and SM |
| Study selection | 9 | The identification of relevant abstracts. the selection of studies. and extraction were performed independently by two of the authors (B.R. and A.B.) and conflicts resolved by a fourth investigator (M.M.). | 5 |
| Data collection process | 10 | The principal characteristics of the included trials included were reported in Table 5S. | 6 and SM |
| Data items | 11 | Information on the baseline characteristics of the samples enrolled (age, gender, proportion of people with diabetes, baseline Body Mass Index – BMI), Total Body Weight Loss (TBWL%), Quality of Life (QoL), Serious Adverse Events (SAE), and all-cause mortality were extracted from the principal publication, when available (Table 3S of Supplementary Materials reports a detailed list) | 6 and SM |
| Risk of bias in individual studies | 12 | The quality of trials was assessed using the parameters proposed by the Cochrane Collaboration. | 6 |
| Summary measures | 13 | Mantel-Haenszel odds ratio (MH-OR) and Weighted Mean Difference (WMD) with 95% Confidence Interval (95%, CI) were calculated for all outcomes defined above on an intention-to-treat basis. | 5-6 |
| Synthesis of results | 14 | Heterogeneity was assessed by using I^2^ statistics. Random-effects model was applied in the primary analysis. We performed a Network Meta-Analysis (NMA) for all the outcomes listed above, in order to verify differences across individual anti-obesity strategies concerning their effects on primary and secondary endpoints. | 6 |
| **Section/topic** | **#** | **Checklist item** | **Reported on page #** |
| Risk of bias across studies | 15 | The risk of bias was assessed using the Cochrane recommended tool to determine the risk of bias in RCTs | 5 |
| Additional analyses | 16 | Several prespecified subgroup analyses were performed for the following baseline variables: different types of anti-obesity strategies (i.e., surgical and endoscopic procedures, and anti-obesity drugs), BMI categories (mean BMI at the enrolment <30, 30-34.9, 35-39.9, and >40 Kg/m2), and type 2 diabetes mellitus (T2DM; (yes: RCT enrolling at least 75% of subjects with diabetes; no: RCT enrolling no more than 25% of subjects with T2DM). Traditional meta-analyses were performed for all the endpoints for placebo- and active-controlled trials. | 5-6 |
| **RESULTS** | | |  |
| Study selection | 17 | The search of Medline and Embase database allowed the identification of 139 trials (i.e., 150 comparisons). | 7 and SM |
| Study characteristics | 18 | The principal characteristics of included trials were reported in Supplementary Materials (Table 5S). | 7 and SM |
| Risk of bias within studies | 19 | The quality of studies was heterogeneous. | 7 and SM |
| Results of individual studies | 20 | Forest plots for any primary and secondary endpoint are reported in the Results section and Supplementary Materials. | 8-10; SM |
| Synthesis of results | 21 | In trials enrolling subjects in class I of obesity, tirzepatide resulted in equal effectiveness to both OAGB and RYGB, and it was significantly superior to all the other comparisons. In trials on class II of obesity, tirzepatide was significantly superior to all the other comparisons and inferior to both OAGB and RYGB. Semaglutide was associated with a higher TBWL% than the other OMMs (with the notable exception of tirzepatide), and it was equally effective to EBP, GCP, and LAGB. In trials enrolling patients with a mean BMI>40 kg/m2, the procedure with the highest estimated weight loss was BPD. Semaglutide was statistically less effective than SG and gastric bypass, but not inferior to GCP and LAGB. Both RYGB and OAGB were superior to SG. | 8-10; SM |
| Risk of bias across studies | 22 | The certainty of the evidence evaluated by CINeMA for the primary endpoint (i.e., endpoint TBWL%) for all comparisons is presented in Figure 33S-36S of the Supplementary Materials. For class I of obesity, the confidence of evidence was high for all comparisons between OMMs and the reference category, and low or moderate for EBP and MBS. For class II of obesity, a high confidence of evidence was reported for tirzepatide, asemaglutide, RYGB, and OAGB, whereas for all the other treatments the certainty of the evidence renged from low to moderate. For class III of obesity, the confidence of evidence was moderate for all included treatments (all MBS), with the notable exception for semaglutide (“high”). | 14 and SM |
| Additional analysis | 23 | Sensitivity analyses of trials are available at pg. 8-9. | 8-9 and SM |
| **DISCUSSION** | | |  |
| Summary of evidence | 24 | The GRADE profile classified the strength as moderate for the primary endpoint. | 15 and SM |
| Limitations | 25 | Limitations were reported in extenso in the Discussion section. | 16-13 |
| Conclusions | 26 | In patients affected by mild to moderate obesity, newer OMMs (i.e., tirzepatide and semaglutide) appear to be valid alternatives to EBP and MBS. They could be preliminarily chosen as a first-line option based on similar efficacy and greater safety and tolerability. Higher degrees of obesity could be more effectively treated with MBS, the efficacy of which, with the notable exception of LAGB and GCP, appears superior to other treatments, especially in the long term. | 18, 13 |
| **FUNDING** | | |  |
| Funding | 27 | This research was performed as a part of the institutional activity of the unit, with no specific funding. | 18 |

# Table 2S – Detailed information on search strategy

| **Limits:** Human studies; any date up to January 31st. 2025  **N= 1,432** |
| --- |
| **Search string:** (obesity or overweight) AND (orlistat OR naltrexone OR bupropion OR liraglutide OR semaglutide OR tirzepatide OR Sleeve Gastrectomy OR Roux en Y Gastric Bypass OR One Anastomosis Gastric Bypass OR Laparoscopic Adjustable Gastric Banding OR Biliopancreatic Diversion OR Single Anastomosis Duodenal-Ileal bypass OR Intragastric Balloons OR Primary Obesity Surgery Endoluminal OR Endoscopic Sleeve Gastroplasty)  **Pubmed**  (("obeses"[All Fields] OR "obesity"[MeSH Terms] OR "obesity"[All Fields] OR "obese"[All Fields] OR "obesities"[All Fields] OR "obesity s"[All Fields] OR ("overweight"[MeSH Terms] OR "overweight"[All Fields] OR "overweighted"[All Fields] OR "overweightness"[All Fields] OR "overweights"[All Fields])) AND ("orlistat"[Supplementary Concept] OR "orlistat"[All Fields] OR "orlistat"[MeSH Terms] OR "orlistat s"[All Fields] OR ("naltrexone"[Supplementary Concept] OR "naltrexone"[All Fields] OR "naltrexon"[All Fields] OR "naltrexone"[MeSH Terms] OR "naltrexone s"[All Fields]) OR ("bupropion"[Supplementary Concept] OR "bupropion"[All Fields] OR "amfebutamone"[All Fields] OR "bupropion"[MeSH Terms] OR "bupropion s"[All Fields] OR "bupropione"[All Fields]) OR ("liraglutid"[All Fields] OR "liraglutide"[Supplementary Concept] OR "liraglutide"[All Fields] OR "liraglutide"[MeSH Terms] OR "liraglutide s"[All Fields]) OR ("semaglutide"[Supplementary Concept] OR "semaglutide"[All Fields]) OR ("tirzepatide"[Supplementary Concept] OR "tirzepatide"[All Fields] OR "tirzepatide"[MeSH Terms]) OR (("sleeve"[All Fields] OR "sleeved"[All Fields] OR "sleeves"[All Fields] OR "sleeving"[All Fields]) AND ("gastrectomy"[MeSH Terms] OR "gastrectomy"[All Fields] OR "gastrectomies"[All Fields])) OR ("gastric bypass"[MeSH Terms] OR ("gastric"[All Fields] AND "bypass"[All Fields]) OR "gastric bypass"[All Fields] OR "roux en y gastric bypass"[All Fields]) OR ("One"[All Fields] AND ("anastomosis, surgical"[MeSH Terms] OR ("anastomosis"[All Fields] AND "surgical"[All Fields]) OR "surgical anastomosis"[All Fields] OR "anastomosis"[All Fields]) AND ("gastric bypass"[MeSH Terms] OR ("gastric"[All Fields] AND "bypass"[All Fields]) OR "gastric bypass"[All Fields])) OR (("laparoscopes"[MeSH Terms] OR "laparoscopes"[All Fields] OR "laparoscope"[All Fields] OR "laparoscopical"[All Fields] OR "laparoscopically"[All Fields] OR "laparoscopics"[All Fields] OR "laparoscopy"[MeSH Terms] OR "laparoscopy"[All Fields] OR "laparoscopic"[All Fields]) AND ("adjustability"[All Fields] OR "adjustable"[All Fields] OR "adjustables"[All Fields] OR "adjustible"[All Fields]) AND ("gastrics"[All Fields] OR "stomach"[MeSH Terms] OR "stomach"[All Fields] OR "gastric"[All Fields]) AND ("banded"[All Fields] OR "banding"[All Fields] OR "bandings"[All Fields])) OR ("biliopancreatic diversion"[MeSH Terms] OR ("biliopancreatic"[All Fields] AND "diversion"[All Fields]) OR "biliopancreatic diversion"[All Fields]) OR (("single person"[MeSH Terms] OR ("single"[All Fields] AND "person"[All Fields]) OR "single person"[All Fields] OR "single"[All Fields] OR "singles"[All Fields]) AND ("anastomosis, surgical"[MeSH Terms] OR ("anastomosis"[All Fields] AND "surgical"[All Fields]) OR "surgical anastomosis"[All Fields] OR "anastomosis"[All Fields]) AND "Duodenal-Ileal"[All Fields] AND ("bypass"[All Fields] OR "bypassed"[All Fields] OR "bypasses"[All Fields] OR "bypassing"[All Fields])) OR (("intragastral"[All Fields] OR "intragastrally"[All Fields] OR "intragastric"[All Fields] OR "intragastrical"[All Fields] OR "intragastrically"[All Fields]) AND ("balloon"[All Fields] OR "balloon s"[All Fields] OR "balloons"[All Fields])) OR (("primaries"[All Fields] OR "primary"[All Fields]) AND ("obes surg"[Journal] OR ("obesity"[All Fields] AND "surgery"[All Fields]) OR "obesity surgery"[All Fields]) AND ("endoluminal"[All Fields] OR "endoluminally"[All Fields])) OR (("endoscope s"[All Fields] OR "endoscoped"[All Fields] OR "endoscopes"[MeSH Terms] OR "endoscopes"[All Fields] OR "endoscope"[All Fields] OR "endoscopical"[All Fields] OR "endoscopically"[All Fields] OR "endoscopy"[MeSH Terms] OR "endoscopy"[All Fields] OR "endoscopic"[All Fields]) AND ("sleeve"[All Fields] OR "sleeved"[All Fields] OR "sleeves"[All Fields] OR "sleeving"[All Fields]) AND ("gastroplasty"[MeSH Terms] OR "gastroplasty"[All Fields] OR "gastroplasties"[All Fields])))) AND ((randomizedcontrolledtrial[Filter]) AND (1967/1/1:2025/1/31[pdat]))  Translations  obesity: "obeses"[All Fields] OR "obesity"[MeSH Terms] OR "obesity"[All Fields] OR "obese"[All Fields] OR "obesities"[All Fields] OR "obesity's"[All Fields]  overweight: "overweight"[MeSH Terms] OR "overweight"[All Fields] OR "overweighted"[All Fields] OR "overweightness"[All Fields] OR "overweights"[All Fields]  orlistat: "orlistat"[Supplementary Concept] OR "orlistat"[All Fields] OR "orlistat"[MeSH Terms] OR "orlistat's"[All Fields]  naltrexone: "naltrexone"[Supplementary Concept] OR "naltrexone"[All Fields] OR "naltrexon"[All Fields] OR "naltrexone"[MeSH Terms] OR "naltrexone's"[All Fields]  bupropion: "bupropion"[Supplementary Concept] OR "bupropion"[All Fields] OR "amfebutamone"[All Fields] OR "bupropion"[MeSH Terms] OR "bupropion's"[All Fields] OR "bupropione"[All Fields]  liraglutide: "liraglutid"[All Fields] OR "liraglutide"[Supplementary Concept] OR "liraglutide"[All Fields] OR "liraglutide"[MeSH Terms] OR "liraglutide's"[All Fields]  semaglutide: "semaglutide"[Supplementary Concept] OR "semaglutide"[All Fields]  tirzepatide: "tirzepatide"[Supplementary Concept] OR "tirzepatide"[All Fields] OR "tirzepatide"[MeSH Terms]  Sleeve: "sleeve"[All Fields] OR "sleeved"[All Fields] OR "sleeves"[All Fields] OR "sleeving"[All Fields]  Gastrectomy: "gastrectomy"[MeSH Terms] OR "gastrectomy"[All Fields] OR "gastrectomies"[All Fields]  Roux en Y Gastric Bypass: "gastric bypass"[MeSH Terms] OR ("gastric"[All Fields] AND "bypass"[All Fields]) OR "gastric bypass"[All Fields] OR "roux en y gastric bypass"[All Fields]  Anastomosis: "anastomosis, surgical"[MeSH Terms] OR ("anastomosis"[All Fields] AND "surgical"[All Fields]) OR "surgical anastomosis"[All Fields] OR "anastomosis"[All Fields]  Gastric Bypass: "gastric bypass"[MeSH Terms] OR ("gastric"[All Fields] AND "bypass"[All Fields]) OR "gastric bypass"[All Fields]  Laparoscopic: "laparoscopes"[MeSH Terms] OR "laparoscopes"[All Fields] OR "laparoscope"[All Fields] OR "laparoscopical"[All Fields] OR "laparoscopically"[All Fields] OR "laparoscopics"[All Fields] OR "laparoscopy"[MeSH Terms] OR "laparoscopy"[All Fields] OR "laparoscopic"[All Fields]  Adjustable: "adjustability"[All Fields] OR "adjustable"[All Fields] OR "adjustables"[All Fields] OR "adjustible"[All Fields]  Gastric: "gastrics"[All Fields] OR "stomach"[MeSH Terms] OR "stomach"[All Fields] OR "gastric"[All Fields]  Banding: "banded"[All Fields] OR "banding"[All Fields] OR "bandings"[All Fields]  Biliopancreatic Diversion: "biliopancreatic diversion"[MeSH Terms] OR ("biliopancreatic"[All Fields] AND "diversion"[All Fields]) OR "biliopancreatic diversion"[All Fields]  Single: "single person"[MeSH Terms] OR ("single"[All Fields] AND "person"[All Fields]) OR "single person"[All Fields] OR "single"[All Fields] OR "singles"[All Fields]  Anastomosis: "anastomosis, surgical"[MeSH Terms] OR ("anastomosis"[All Fields] AND "surgical"[All Fields]) OR "surgical anastomosis"[All Fields] OR "anastomosis"[All Fields]  bypass: "bypass"[All Fields] OR "bypassed"[All Fields] OR "bypasses"[All Fields] OR "bypassing"[All Fields]  Intragastric: "intragastral"[All Fields] OR "intragastrally"[All Fields] OR "intragastric"[All Fields] OR "intragastrical"[All Fields] OR "intragastrically"[All Fields]  Balloons: "balloon"[All Fields] OR "balloon's"[All Fields] OR "balloons"[All Fields]  Primary: "primaries"[All Fields] OR "primary"[All Fields]  Obesity Surgery: "Obes Surg"[Journal:__jid9106714] OR ("obesity"[All Fields] AND "surgery"[All Fields]) OR "obesity surgery"[All Fields]  Endoluminal: "endoluminal"[All Fields] OR "endoluminally"[All Fields]  Endoscopic: "endoscope's"[All Fields] OR "endoscoped"[All Fields] OR "endoscopes"[MeSH Terms] OR "endoscopes"[All Fields] OR "endoscope"[All Fields] OR "endoscopical"[All Fields] OR "endoscopically"[All Fields] OR "endoscopy"[MeSH Terms] OR "endoscopy"[All Fields] OR "endoscopic"[All Fields]  Sleeve: "sleeve"[All Fields] OR "sleeved"[All Fields] OR "sleeves"[All Fields] OR "sleeving"[All Fields]  Gastroplasty: "gastroplasty"[MeSH Terms] OR "gastroplasty"[All Fields] OR "gastroplasties"[All Fields]  **EMBASE**  **N= 2,106**  ('obesity'/exp OR obesity OR 'overweight'/exp OR overweight) AND ('orlistat'/exp OR orlistat OR 'naltrexone'/exp OR naltrexone OR 'bupropion'/exp OR bupropion OR 'liraglutide'/exp OR liraglutide OR 'semaglutide'/exp OR semaglutide OR 'tirzepatide'/exp OR tirzepatide OR 'sleeve gastrectomy'/exp OR 'sleeve gastrectomy' OR (sleeve AND ('gastrectomy'/exp OR gastrectomy)) OR 'roux en y gastric bypass'/exp OR 'roux en y gastric bypass' OR (roux AND ('en'/exp OR en) AND y AND gastric AND ('bypass'/exp OR bypass)) OR 'one anastomosis gastric bypass'/exp OR 'one anastomosis gastric bypass' OR (('one'/exp OR one) AND ('anastomosis'/exp OR anastomosis) AND gastric AND ('bypass'/exp OR bypass)) OR 'laparoscopic adjustable gastric banding'/exp OR 'laparoscopic adjustable gastric banding' OR (laparoscopic AND adjustable AND gastric AND banding) OR 'biliopancreatic diversion'/exp OR 'biliopancreatic diversion' OR (biliopancreatic AND ('diversion'/exp OR diversion)) OR 'single anastomosis duodenal-ileal bypass' OR (single AND ('anastomosis'/exp OR anastomosis) AND 'duodenal ileal' AND ('bypass'/exp OR bypass)) OR 'intragastric balloons' OR (intragastric AND balloons) OR 'primary obesity surgery endoluminal'/exp OR 'primary obesity surgery endoluminal' OR (primary AND ('obesity'/exp OR obesity) AND ('surgery'/exp OR surgery) AND endoluminal) OR 'endoscopic sleeve gastroplasty'/exp OR 'endoscopic sleeve gastroplasty' OR (endoscopic AND sleeve AND ('gastroplasty'/exp OR gastroplasty)) OR 'aspiration therapy'/exp OR 'aspiration therapy' OR (('aspiration'/exp OR aspiration) AND ('therapy'/exp OR therapy)) AND [embase]/lim NOT ([embase]/lim AND [medline]/lim) AND ('controlled clinical trial'/de OR 'double blind procedure'/de OR 'phase 2 clinical trial topic'/de OR 'phase 3 clinical trial'/de OR 'phase 3 clinical trial topic'/de OR 'randomized controlled trial'/de OR 'randomized controlled trial topic'/de)  **CENTRAL**  **N= 89**  (obesity or overweight) AND (orlistat OR naltrexone OR bupropion OR liraglutide OR semaglutide OR tirzepatide OR Sleeve Gastrectomy OR Roux en Y Gastric Bypass OR One Anastomosis Gastric Bypass OR Laparoscopic Adjustable Gastric Banding OR Biliopancreatic Diversion OR Single Anastomosis Duodenal-Ileal bypass OR Intragastric Balloons OR Primary Obesity Surgery Endoluminal OR Endoscopic Sleeve Gastroplasty) in All Text |
| **Additional search:**  Additional manual search of the references of included trials and former meta-analyses was carried out to identify other newly published and unpublished studies. Completed but yet unpublished studies with the procedures specified above were searched in the www.clinicaltrials.gov register. using the same search string as above. |


# Table 3S. Information collected for each trial

| First author |
| --- |
| Publication year |
| National Clinical Trial (NCT) number or other registration identifiers/acronyms |
| Pharmacological (doses), surgical, and endoscopic procedures |
| Sample size |
| Duration of the trial |
| Minimum and maximum body mass index (BMI) |
| Minimum and maximum age |
| Baseline BMI |
| Mean age |
| Proportion of women |
| Proportion of patients with type 2 diabetes |
| Total body weight loss (TBWL%) |
| Waist circumference |
| Body composition |
| Proportion of patients achieving at least 5%, 10%, 15%, 20%, and 25% |
| Remission or improvement/resolution of obesity-associated medical conditions (OAMC) |
| Serious adverse events (SAE) |
| Mortality |
| Major adverse cardiovascular events (MACE) |
| Fasting plasma glucose (FPG) |
| Glycated hemoglobin (HbA1c) |
| Lipid profile |
| Estimated glomerular filtration rate (eGFR), creatinine, albuminuria |
| Mental health parameters |
| Quality of life (QoL) |

# Table 4S. Excluded trials and reasons for the exclusion.

| **N** | **Study** | **Publication year** | **Reason for the exclusion** |
| --- | --- | --- | --- |
| 1 | De Moura | 2019 | Short treatment/follow-up period |
| 2 | Pajecki | 2023 | Duplicate |
| 3 | Aldhwayan | 2022 | Duplicate |
| 4 | Newsome | 2021 | Submaximal doses |
| 5 | De Moura | 2019 | Not approved intervention for obesity |
| 6 | Fidler | 2011 | Not approved intervention for obesity |
| 7 | Bohula | 2018 | Not approved intervention for obesity |
| 8 | O'Neil | 2012 | Not approved intervention for obesity |
| 9 | Scirica | 2019 | Not approved intervention for obesity |
| 10 | Smith | 2010 | Not approved intervention for obesity |
| 11 | Mittemair | 2007 | Not approved intervention for obesity |
| 12 | Horwitz | 2014 | Not RCT |
| 13 | Mollan | 2021 | Same type of surgical procedure in both arms |
| 14 | Inagaki | 2022 | Not on obesity |

# Table 5S. Principal baseline characteristics of the included studies

| **n** | **Study Name** | **Intervention** | **Comparator** | **N. pat.**  **Interv.** | **N. pat.**  **Comp.** | **Trial duration**  (weeks) | **BMI min.**  (Kg/m^2^) | **BMI max.**  (Kg/m^2^) | **Age max.**  (years) | **BMI**  (Kg/m^2^) | **Age**  (years) | **Women**  (%) | **DM**  (%) |
| --- | --- | --- | --- | --- | --- | --- | --- | --- | --- | --- | --- | --- | --- |
| **OMM** | | | | | | | | | | | | | |
| ***BMI 30 – 34.9 kg/m^2^*** | | | | | | | | | | | | | |
| 1 | *Aronne^50^* | Tirzepatide | Placebo | 335 | 335 | 52 | 27 | NR | NR | 30.5 | 49 | 70 | 0 |
| 2 | *Kadowaki^44^* | Semaglutide | Placebo | 199 | 101 | 68 | 27 | NR | NR | 31.9 | 51 | 37 | 25 |
| 3 | *Gudbergsen^2^* | Liraglutide | Placebo | 80 | 76 | 52 | 27 | NR | 74 | 32.1 | 59 | 65 | NR |
| 4 | *Derosa^19^* | Orlistat | Placebo | 53 | 47 | 52 | 30 | NR | NR | 32.1 | 51 | 52 | 0 |
| 5 | *Lundgren^3^* | Liraglutide | Placebo | 98 | 97 | 52 | 32 | 43 | 65 | 32.6 | 43 | 64 | 0 |
| 6 | *Berne^16^* | Orlistat | Placebo | 111 | 109 | 52 | 28 | 40 | 75 | 32.7 | 59 | 45 | 100 |
| 7 | *Derosa^18^* | Orlistat | Placebo | 126 | 128 | 52 | 30 | NR | NR | 32.8 | 52 | 50 | 100 |
| 8 | *Hill^21^* | Orlistat | Placebo | 179 | 184 | 52 | 28 | 34 | NR | 32.8 | 46 | 85 | 0 |
| 9 | *Lincoff^49^* | Semaglutide | Placebo | 8803 | 8801 | 104 | 27 | NR | NR | 33.3 | 62 | 28 | 0 |
| 10 | *Hollander^31^* | Orlistat | Placebo | 162 | 159 | 57 | 28 | 40 | NR | 34.2 | 55 | 50 | 100 |
| 11 | *Rubino^45^* | Semaglutide | Placebo | 535 | 268 | 68 | 27 | NR | NR | 34.3 | 46 | 78 | 0 |
| 12 | *Astrup^1^* | Liraglutide | Orlistat | 93 | 95 | 52 | 30 | 40 | 65 | 34.4 | 46 | 75 | 3 |
|  |  |  | Placebo |  | 98 | 52 | 30 | 40 | 65 | 34.8 | 46 | 75 | 4 |
| ***BMI 35 – 39.9 kg/m^2^*** | | | | | | | | | | | | | |
| 14 | *Rossner^34^* | Orlistat | Placebo | 244 | 243 | 104 | 28 | 43 | NR | 35.0 | 44 | 85 | NR |
| 15 | *Miles^25^* | Orlistat | Placebo | 250 | 254 | 52 | 28 | 43 | 65 | 35.4 | 53 | 48 | 100 |
| 16 | *Garvey^6^* | Liraglutide | Placebo | 195 | 197 | 56 | 27 | NR | NR | 35.5 | 57 | 53 | 100 |
| 17 | *Wadden^7^* | Liraglutide | Placebo | 212 | 210 | 56 | 27 | NR | NR | 35.6 | 46 | 81 | 0 |
| 18 | *Bakris^15^* | Orlistat | Placebo | 267 | 265 | 52 | 28 | 43 | NR | 35.6 | 53 | 61 | 8 |
| 19 | *Kelley^24^* | Orlistat | Placebo | 266 | 269 | 52 | 28 | 40 | 65 | 35.7 | 58 | 56 | 100 |
| 20 | *Karhunen^23^* | Orlistat | Placebo | 36 | 36 | 52 | 30 | 43 | NR | 35.8 | 44 | 77 | 0 |
| 21 | *Davies^43^* | Semaglutide | Placebo | 404 | 403 | 68 | 27 | NR | NR | 35.9 | 55 | 51 | 100 |
| 22 | *Sjostrom^27^* | Orlistat | Placebo | 343 | 340 | 52 | 28 | 47 | NR | 36.0 | 45 | 83 | NR |
| 23 | *Hauptman^33^* | Orlistat | Placebo | 210 | 212 | 104 | 30 | 44 | NR | 36.0 | 42 | 68 | NR |
| 24 | *Apovian^10^* | Naltr./Bupr. | Placebo | 992 | 492 | 56 | 30 | 45 | 65 | 36.1 | 44 | 85 | 0 |
| 25 | *Greenway^11^* | Naltr./Bupr. | Placebo | 583 | 581 | 56 | 27 | 45 | 65 | 36.1 | 44 | 85 | 0 |
| 26 | *Krempf^32^* | Orlistat | Placebo | 346 | 350 | 78 | 28 | NR | 65 | 36.1 | 41 | 86 | 0 |
| 27 | *Loomba ^51^* | Tirzepatide | Placebo | 95 | 48 | 52 | 27 | 50 | 80 | 36.1 | 54 | 57 | 58 |
| 28 | *Garvey^53^* | Tirzepatide | Placebo | 623 | 315 | 72 | 27 | NR | NR | 36.1 | 54 | 51 | 100 |
| 29 | *Davidson^17^* | Orlistat | Placebo | 657 | 223 | 52 | 30 | 43 | NR | 36.3 | 44 | 84 | 0 |
| 30 | *Hollander^12^* | Naltr./Bupr. | Placebo | 335 | 170 | 56 | 27 | 45 | 70 | 36.4 | 54 | 55 | 100 |
| 31 | *Wadden^13^* | Naltr./Bupr. | Placebo | 591 | 202 | 56 | 27 | 45 | 65 | 36.5 | 46 | 90 | 0 |
| 32 | *Nissen^14^* | Naltr./Bupr. | Placebo | 4455 | 4450 | 156 | 27 | 50 | NR | 36.6 | 61 | 54 | 85 |
| 33 | *Finer^20^* | Orlistat | Placebo | 110 | 108 | 52 | 30 | 43 | NR | 36.8 | 41 | 89 | 0 |
| 34 | *Poston^26^* | Orlistat | Placebo | 37 | 35 | 52 | 27 | NR | 65 | 36.8 | 43 | 100 | 11 |
| 35 | *Kosiborod^39^* | Semaglutide | Placebo | 310 | 306 | 52 | 30 | NR | NR | 36.9 | 70 | 44 | 100 |
| 36 | *Kosiborod^40^* | Semaglutide | Placebo | 263 | 266 | 52 | 30 | NR | NR | 37.0 | 69 | 56 | 0 |
| 37 | *Davies^5^* | Liraglutide | Placebo | 423 | 212 | 56 | 27 | NR | NR | 37.2 | 55 | 51 | 100 |
| 38 | *Togerson^36^* | Orlistat | Placebo | 1640 | 1637 | 208 | 30 | NR | 60 | 37.3 | 43 | 55 | 0 |
| 39 | *James^22^* | Orlistat | Placebo | 23 | 23 | 52 | 30 | 43 | NR | 37.5 | 43 | 85 | 0 |
| 40 | *Svendsen^28^* | Orlistat | Placebo | 23 | 21 | 52 | 30 | NR | 63 | 37.5 | 48 | 55 | NR |
| 41 | *Richelsen^35^* | Orlistat | Placebo | 153 | 156 | 156 | 30 | 45 | 65 | 37.5 | 47 | 51 | 20 |
| 42 | *Swinburn^29^* | Orlistat | Placebo | 170 | 169 | 52 | 30 | 50 | 70 | 37.8 | 52 | 57 | 8 |
| 43 | *Wilding^47^* | Semaglutide | Placebo | 1306 | 655 | 68 | 27 | NR | NR | 37.9 | 46 | 75 | 0 |
| 44 | *Wadden^46^* | Semaglutide | Placebo | 407 | 204 | 68 | 27 | NR | NR | 38.0 | 46 | 82 | 0 |
| 45 | *Jastreboff^54^* | Tirzepatide | Placebo | 1266 | 643 | 72 | 27 | NR | NR | 38.0 | 45 | 67 | 0 |
| 46 | *Garvey^48^* | Semaglutide | Placebo | 152 | 152 | 104 | 27 | NR | NR | 38.5 | 47 | 77 | 0 |
| 47 | *Le Roux^9^* | Liraglutide | Placebo | 1505 | 749 | 156 | 27 | NR | NR | 38.9 | 47 | 76 | 0 |
| 48 | *Wadden^8^* | Liraglutide | Placebo | 142 | 140 | 56 | 30 | NR | NR | 39.0 | 47 | 84 | 0 |
| 49 | *Malhotra^52^* | Tirzepatide | Placebo | 234 | 235 | 52 | 30 | NR | NR | 39.0 | 50 | 30 | 0 |
| 50 | *O'Neil^4^* | Liraglutide | Placebo | 103 | 136 | 52 | 30 | NR | NR | 39.3 | 47 | 35 | 0 |
| 51 | *Loomba^30^* | Semaglutide | Placebo | 47 | 24 | 52 | 27 | NR | 75 | 35.0 | 59 | 70 | 75 |
| ***BMI> 39.9 kg/m^2^*** | | | | | | | | | | | | | |
| 52 | *McGowan^41^* | Semaglutide | Placebo | 138 | 69 | 52 | 30 | NR | NR | 40.1 | 53 | 71 | 0 |
| 53 | *Bliddal^42^* | Semaglutide | Placebo | 271 | 136 | 68 | 30 | NR | NR | 40.3 | 56 | 82 | 0 |
| **Total (mean value) in trials on OMM** | | | | **32,162** | **26,855** | **68** | **29** | **44** | **67** | **36.1** | **50** | **66** | **28** |

| **EBP** | | | | | | | | | | | | | |
| --- | --- | --- | --- | --- | --- | --- | --- | --- | --- | --- | --- | --- | --- |
| ***BMI 30 – 34.9 kg/m^2^*** | | | | | | | | | | | | | |
| 1 | *Lee^71^* | IGB | Placebo | 8 | 10 | 26 | 27 | NR | 65 | 31.3 | 45 | 40 | 11 |
| 2 | *Dargent^66^* | IGB | No therapy | 68 | 32 | 24 | 27 | NR | 65 | 34.1 | 37 | 90 | 4 |
| 3 | *Courcoulas^67^* | IGB | No therapy | 135 | 136 | 26 | 30 | 40 | 65 | 34.9 | 39 | 90 | 7 |
| 4 | *Ponce^69^* | IGB | No therapy | 21 | 9 | 26 | 30 | 40 | 60 | 34.9 | 42 | 90 | NR |
| ***BMI 35 – 39.9 kg/m^2^*** | | | | | | | | | | | | | |
| 5 | *Ponce^69^* | IGB | Placebo | 187 | 139 | 24 | 30 | 40 | 60 | 35.3 | 44 | 95 | 7 |
| 6 | *Sullivan^72^* | IGB | Placebo | 198 | 189 | 26 | 30 | 40 | 64 | 35.3 | 43 | 87 | 0 |
| 7 | *Abu Dayyeh^63^* | ESG | No therapy | 77 | 110 | 52 | 30 | 40 | 65 | 35.6 | 47 | 85 | 25 |
| 8 | *Abu Dayyeh^70^* | IGB | No therapy | 187 | 101 | 32 | 30 | 40 | 65 | 35.8 | 44 | 88 | NR |
| 9 | *Sullivan^74^* | POSE | Placebo | 221 | 111 | 52 | 30 | 40 | 60 | 36.1 | 45 | 89 | 9 |
| 10 | *Fuller^68^* | IGB | No therapy | 37 | 37 | 26 | 30 | 40 | 60 | 36.3 | 45 | 67 | NR |
| 11 | *Miller^73^* | POSE | No therapy | 30 | 9 | 52 | 30 | 40 | 60 | 36.5 | 39 | 67 | 5 |
| ***BMI> 39.9 kg/m^2^*** | | | | | | | | | | | | | |
| 12 | *Genco^65^* | IGB | Lifestyle | 25 | 25 | 26 | 40 | 45 | 35 | 41.2 | 31 | 70 | NR |
| 13 | *Coffin^64^* | IGB | Lifestyle | 55 | 60 | 26 | 45 | NR | 65 | 54.3 | 40 | 73 | 30 |
| **Total (mean value) in trials on EP** | | | | **1,017** | **781** | **35** | **31** | **44** | **61** | **37.1** | **44** | **75** | **41** |

| **MBS** | | | | | | | | | | | | | |
| --- | --- | --- | --- | --- | --- | --- | --- | --- | --- | --- | --- | --- | --- |
| ***BMI 27 – 29.9 kg/m^2^*** | | | | | | | | | | | | | |
| 1 | *Cheng^101^* | RYGB | Lifestyle | 12 | 14 | 260 | 27 | 32 | 65 | 29.0 | 44 | 35 | 100 |
| ***BMI 30 – 34.9 kg/m^2^*** | | | | | | | | | | | | | |
| 2 | *Liang^108^* | RYGB | No therapy | 31 | 70 | 52 | 28 | NR | 30 | 30.4 | 51 | 33 | 8 |
| 3 | *Lee^91^* | OAGB | SG | 30 | 30 | 260 | 27 | 35 | 60 | 30.6 | 45 | 71 | 17 |
| 4 | *Yang^129^* | RYGB | SG | 32 | 32 | 156 | 28 | 35 | 60 | 32.0 | 41 | 66 | 100 |
| 5 | *Cohen^100^* | RYGB | Lifestyle | 51 | 49 | 104 | 30 | 35 | 65 | 32.5 | 51 | 45 | 100 |
| 6 | *O'Brien^77^* | LAGB | Lifestyle | 40 | 10 | 520 | 30 | 35 | 50 | 33.6 | 41 | 86 | NR |
| 7 | *Ikramuddin^110^* | RYGB | No therapy | 57 | 56 | 260 | 30 | 40 | 67 | 34.6 | 48 | 60 | 100 |
| ***BMI 35 – 39.9 kg/m^2^*** | | | | | | | | | | | | | |
| 8 | *Xiang^82^* | LAGB | No therapy | 44 | 44 | 104 | 30 | 40 | 65 | 35.3 | 34 | 78 | 7 |
| 9 | *Courcolas^99^* | RYGB | Lifestyle | 20 | 20 | 260 | 30 | 40 | 55 | 35.7 | 47 | 82 | 100 |
| 10 | *Courcolas^100^* | RYGB | LAGB | 20 | 21 | 260 | 30 | 40 | 55 | 35.7 | 47 | 82 | 24 |
| 11 | *Courcolas^101^* | LAGB | Lifestyle | 21 | 20 | 260 | 30 | 40 | 55 | 35.7 | 47 | 82 | NR |
| 12 | *Simonson^76^* | LAGB | Lifestyle | 23 | 22 | 156 | 30 | 45 | 65 | 36.5 | 51 | 45 | 100 |
| 13 | *Schauer^102^* | RYGB | Lifestyle | 50 | 50 | 260 | 27 | 43 | 60 | 36.7 | 49 | 66 | NR |
| 14 | *Schauer^103^* | RYGB | SG | 50 | 50 | 260 | 27 | 43 | 60 | 36.7 | 49 | 66 | 27 |
| 15 | *Schauer^104^* | SG | Lifestyle | 50 | 50 | 260 | 27 | 43 | 60 | 36.7 | 49 | 66 | 6 |
| 16 | *Schiavon^109^* | RYGB | No therapy | 50 | 50 | 156 | 30 | 40 | 65 | 36.9 | 44 | 76 | 100 |
| 17 | *Dixon^80^* | LAGB | No therapy | 30 | 30 | 104 | 30 | 40 | 60 | 37.1 | 47 | 54 | 33 |
| 18 | *Tang^123^* | RYGB | SG | 40 | 40 | 104 | 28 | NR | 65 | 38.1 | 38 | 57 | 100 |
| 19 | *Cummings^107^* | RYGB | No therapy | 15 | 17 | 52 | 30 | 40 | 65 | 38.3 | 53 | 69 | 100 |
| 20 | *Zhang^132^* | RYGB | SG | 32 | 32 | 260 | 32 | 50 | 60 | 39.0 | 31 | 59 | 100 |
| 21 | *Casajoana^97^* | RYGB | GCP | 15 | 15 | 260 | 35 | 43 | 60 | 39.5 | 49 | 66 | 100 |
| 22 | *Casajoana^98^* | RYGB | SG | 15 | 15 | 260 | 35 | 43 | 60 | 39.5 | 49 | 60 | 8 |
| 23 | *Casajoana^99^* | SG | GCP | 15 | 15 | 260 | 35 | 43 | 60 | 39.5 | 49 | 72 | 56 |
| 24 | *Delko^111^* | RYGB | OAGB | 40 | 40 | 52 | 35 | 50 | NR | 39.8 | 40 | 89 | 35 |
| ***BMI> 39.9 kg/m^2^*** | | | | | | | | | | | | | |
| 25 | *Wallenius^124^* | RYGB | SG | 25 | 24 | 104 | 35 | 60 | 60 | 40.2 | 48 | 47 | 75 |
| 26 | *Spaggiari^138^* | SG | Lifestyle | 11 | 9 | 52 | 35 | NR | NR | 41.8 | 45 | 56 | 100 |
| 27 | *Olbers^104^* | RYGB | LVGB | 37 | 46 | 104 | 35 | 50 | NR | 42.0 | 35 | 73 | 100 |
| 28 | *Verrastro^122^* | RYGB | SG | 77 | 79 | 52 | 30 | 55 | 70 | 42.1 | 47 | 47 | 100 |
| 29 | *Pullman^133^* | RYGB | SG | 56 | 58 | 364 | 35 | 65 | 55 | 42.1 | 47 | 52 | 100 |
| 30 | *Keidar^119^* | RYGB | SG | 19 | 18 | 52 | 35 | NR | 65 | 42.2 | 49 | 46 | 35 |
| 31 | *Svanevik^128^* | RYGB | SG | 54 | 55 | 156 | 35 | NR | NR | 42.2 | 48 | 66 | 100 |
| 32 | *Werling^105^* | RYGB | LVGB | 37 | 46 | 312 | 35 | 50 | NR | 42.3 | 45 | 73 | 3 |
| 33 | *Lundell^85^* | LVGB | LAGB | 24 | 26 | 52 | 35 | NR | NR | 42.5 | 48 | 54 | NR |
| 34 | *Level^115^* | RYGB | OAGB | 9 | 19 | 260 | 35 | NR | NR | 42.7 | 37 | NR | 27 |
| 35 | *Lee^103^* | RYGB | LVGB | 40 | 40 | 104 | 35 | 60 | 60 | 43.1 | 32 | 70 | Nr |
| 36 | *Nilsell^83^* | LVBG | LAGB | 30 | 29 | 260 | 37 | NR | 60 | 43.4 | 39 | 81 | 3 |
| 37 | *Dowsey^79^* | LAGB | No therapy | 42 | 41 | 52 | 35 | NR | 65 | 43.7 | 58 | 80 | 100 |
| 38 | *Grubnik^137^* | SG | GCP | 27 | 25 | 156 | 35 | 65 | 75 | 43.7 | 42 | 76 | 100 |
| 39 | *Ramon^121^* | RYGB | SG | 7 | 8 | 52 | 35 | 50 | 60 | 43.8 | 48 | NR | 35 |
| 40 | *Robert^116^* | RYGB | OAGB | 117 | 117 | 260 | 35 | NR | 65 | 43.9 | 43 | 75 | 22 |
| 41 | *Peterli^131^* | RYGB | SG | 110 | 107 | 260 | 35 | NR | 65 | 43.9 | 42 | 72 | 100 |
| 42 | *Hany^89^* | OAGB | SG | 150 | 150 | 260 | 35 | NR | 60 | 44.0 | 34 | 77 | 48 |
| 43 | *Biter^117^* | RYGB | SG | 74 | 76 | 52 | 35 | NR | NR | 44.1 | 44 | 82 | 6 |
| 44 | *Morino^78^* | LAGB | LVGB | 49 | 51 | 156 | 40 | 50 | 60 | 44.3 | 38 | 81 | 29 |
| 45 | *Jain^90^* | OAGB | SG | 101 | 100 | 260 | 35 | 60 | 60 | 44.4 | 42 | 36 | 100 |
| 46 | *Scozzari^84^* | LVGB | LAGB | 51 | 49 | 364 | 40 | 50 | 60 | 44.5 | 38 | 81 | 8 |
| 47 | *Skroubis^93^* | RYGB | BPD | 65 | 65 | 416 | 35 | 50 | NR | 44.9 | 35 | 52 | 100 |
| 48 | *Dixon^81^* | LAGB | No therapy | 30 | 30 | 104 | 35 | 55 | 60 | 45.0 | 48 | 42 | 42 |
| 49 | *Mingrone^94^* | RYGB | BPD | 20 | 20 | 520 | 35 | NR | 60 | 45.0 | 44 | 55 | 18 |
| 50 | *Mingrone^95^* | RYGB | No therapy | 20 | 20 | 520 | 35 | NR | 60 | 45.0 | 44 | 50 | 22 |
| 51 | *Mingrone^96^* | BPD | No therapy | 20 | 20 | 520 | 35 | NR | 60 | 45.0 | 44 | 55 | 31 |
| 52 | *Pajecki^125^* | RYGB | SG | 18 | 18 | 152 | 35 | NR | NR | 45.0 | 68 | 85 | 23 |
| 53 | *Catheline^126^* | RYGB | SG | 91 | 186 | 156 | 35 | NR | 60 | 45.3 | 41 | 86 | 17 |
| 54 | *Kehagias^127^* | RYGB | SG | 30 | 30 | 156 | 30 | 50 | NR | 45.4 | 35 | 36 | 100 |
| 55 | *Karagul^113^* | RYGB | OAGB | 20 | 22 | 156 | 35 | NR | 65 | 45.5 | 43 | 82 | 100 |
| 56 | *Karamanakos^118^* | RYGB | SG | 16 | 16 | 52 | 30 | NR | NR | 45.6 | 33 | 84 | 100 |
| 57 | *Singh^114^* | RYGB | OAGB | 24 | 25 | 208 | 30 | NR | NR | 45.8 | 46 | 69 | 11 |
| 58 | *Salminen^134^* | RYGB | SG | 49 | 52 | 520 | 35 | 60 | 60 | 45.9 | 48 | 69 | 18 |
| 59 | *Talebpour^136^* | SG | GCP | 35 | 35 | 104 | 35 | NR | 65 | 46.0 | 37 | 80 | 9 |
| 60 | *Ignat^130^* | RYGB | SG | 45 | 55 | 260 | 40 | 60 | 60 | 46.3 | 35 | 82 | 25 |
| 61 | *Nguyen^98^* | RYGB | LAGB | 111 | 86 | 520 | 35 | 60 | 60 | 46.5 | 43 | 76 | 100 |
| 62 | *Feigel-Guiller^75^* | LAGB | Lifestyle | 5 | 24 | 156 | 35 | NR | 65 | 47.0 | 48 | 67 | 100 |
| 63 | *Paluszkiewicz^120^* | RYGB | SG | 36 | 36 | 52 | 35 | 60 | 60 | 47.3 | 44 | 67 | 27 |
| 64 | *Musella^87^* | OAGB | SG | 32 | 32 | 52 | 35 | NR | 65 | 48.0 | NR | NR | 40 |
| 65 | *Darabi^86^* | OAGB | GCP | 20 | 20 | 52 | 35 | NR | 65 | 48.5 | 35 | 70 | NR |
| 66 | *MacLean^106^* | RYGB | LVGB | 52 | 54 | 156 | 40 | 50 | NR | 49.0 | 39 | NR | 100 |
| 67 | *Eskandaros ^112^* | RYGB | OAGB | 40 | 40 | 52 | 35 | NR | 60 | 50.0 | 36 | 51 | 60 |
| 68 | *Axer^135^* | SADI | BPD | 30 | 30 | 52 | 42 | 72 | NR | 50.0 | 40 | 50 | 23 |
| 69 | *Roushdy^88^* | OAGB | SG | 21 | 21 | 52 | 35 | NR | 60 | 50.2 | 34 | 95 | 4 |
| 70 | *Hedberg^92^* | RYGB | BPD | 23 | 24 | 208 | 48 | NR | NR | 54.5 | 39 | 49 | 13 |
| 71 | *Salte ^95^* | RYGB | BPD | 31 | 29 | 520 | 50 | 60 | 50 | 55.0 | 36 | 70 | 3 |
| **Total (mean value) in trials on MBS** | | | | **2,930** | **3,061** | **201** | **34** | **49** | **61** | **42.1** | **43** | **67** | **56** |

**N.:** Number; **Min.;** Minimum; **Max.:** Maximum; **BMI:** Body Mass Index; **DM:** Proportion of subjects with diabetes enrolled; **Naltr./Bupr.**: Naltrexone/Bupropione; **Phen./Topir.**: Phentermine/Topiramate; **LAGB**: Laparoscopic Adjustable Gastric Banding; **LVBG**: Vertical Banding Gastroplasty; **VG**: Vertical Gastrogastrostomy; **SG**: Sleeve Gastrectomy; **RYGB**: Roux-en-Y Gastric By-Pass; **BPD**: Bilio-Pancreatic Diversion; **GCP**: Greater Curvature Plication Gastric; **OAGB**: One-anastomosis gastric bypass; **DJB**: duodenojejunal bypass; **NR**: Not Reported. **OMM**: Obesity Management Medications; **EP**: Endoscopic Procedures; **MBS**: Metabolic Bariatric Surgery.

Table 6S – Principal characteristics of the studies and comparisons included in the NMA for TBWL% (class I of obesity).

| **Characteristic** | **Value** |
| --- | --- |
| Number of Interventions | 9 |
| Number of Studies | 22 |
| Total Number of Patients in Network | 21,563 |
| Total Possible Pairwise Comparisons | 36 |
| Total Number of Pairwise Comparisons With Direct Data | 9 |
| Is the network connected? | TRUE |
| Number of Two-arm Studies | 16 |
| Number of Multi-Arms Studies | 0 |
| Average Outcome (TBWL, %) | 5.4 |

# Table 7S – Pairwise comparison table for TBWL% at the endpoint for class I of obesity.

Treatments are ranked from best to worst along the leading diagonal for TBWL% at the endpoint for studies with a mean BMI at entry ranging from 30 to 34.9 kg/m^2^. Above the leading diagonal are estimates from pairwise meta-analyses, below the leading diagonal are estimates from network meta-analyses. Relative treatment effects in ranked order for all studies. Blue boxes: p<0.05.


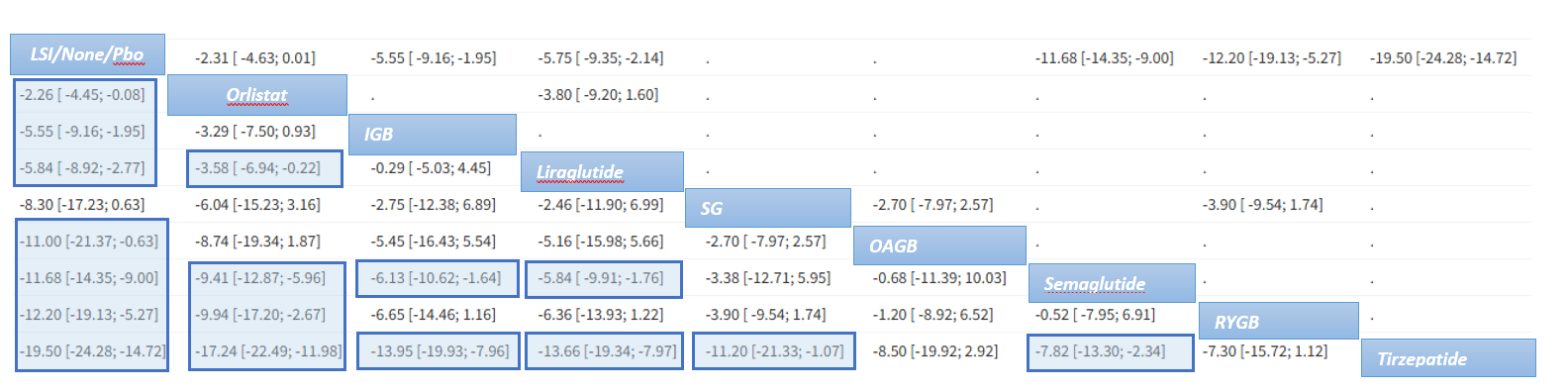


*LSI/None/Pbo: Lifestyle interventions(No therapy/Placebo; IGB: Intragastric Balloon; RYGB: Rou-en-Y Gastric By-pass; OAGB: One-Anastomosis Gastric By-pass; SG: Sleeve Gastrectomy.*

# Table 8S – Assessment of inconsistency for all comparisons for TBWL% at the endpoint in class I of obesity.

# Diff.: difference; CI: Confidence intervals; L/P/None; Lifestyle interventions/Placebo/No interventions. IGB: Intragastric Balloon; RYGB: Rou-en-Y Gastric By-pass; OAGB: One-Anastomosis Gastric By-pass; SG: Sleeve Gastrectomy.

| **Comparison** | **N. Studies** | **NMA** | **Direct** | **Indirect** | **Diff.** | **Diff.**  **95%. CI lower** | **Diff.**  **95%. CI upper** | **p value** |
| --- | --- | --- | --- | --- | --- | --- | --- | --- |
| IGB:Liraglutide | 0 | -0.29 | NA | -0.29 | NA | NA | NA | NA |
| IGB:LSI_None_Pbo | 2 | 5.55 | 5.55 | NA | NA | NA | NA | NA |
| IGB:OAGB | 0 | -5.45 | NA | -5.45 | NA | NA | NA | NA |
| IGB:Orlistat | 0 | 3.29 | NA | 3.29 | NA | NA | NA | NA |
| IGB:RYGB | 0 | -6.65 | NA | -6.65 | NA | NA | NA | NA |
| IGB:Semaglutide | 0 | -6.13 | NA | -6.13 | NA | NA | NA | NA |
| IGB:SG | 0 | -2.75 | NA | -2.75 | NA | NA | NA | NA |
| IGB:Tirzepatide | 0 | -13.95 | NA | -13.95 | NA | NA | NA | NA |
| Liraglutide:LSI_None_Pbo | 2 | 5.84 | 5.75 | 6.11 | -0.36 | -7.26 | 6.54 | 0.92 |
| Liraglutide:OAGB | 0 | -5.16 | NA | -5.16 | NA | NA | NA | NA |
| Liraglutide:Orlistat | 1 | 3.58 | 3.80 | 3.44 | 0.36 | -6.54 | 7.26 | 0.92 |
| Liraglutide:RYGB | 0 | -6.36 | NA | -6.36 | NA | NA | NA | NA |
| Liraglutide:Semaglutide | 0 | -5.84 | NA | -5.84 | NA | NA | NA | NA |
| Liraglutide:SG | 0 | -2.46 | NA | -2.46 | NA | NA | NA | NA |
| Liraglutide:Tirzepatide | 0 | -13.66 | NA | -13.66 | NA | NA | NA | NA |
| OAGB:LSI_None_Pbo | 0 | 11.00 | NA | 11.00 | NA | NA | NA | NA |
| Orlistat:LSI_None_Pbo | 4 | 2.26 | 2.31 | 1.95 | 0.36 | -6.54 | 7.26 | 0.92 |
| RYGB:LSI_None_Pbo | 1 | 12.20 | 12.20 | NA | NA | NA | NA | NA |
| Semaglutide:LSI_None_Pbo | 3 | 11.68 | 11.68 | NA | NA | NA | NA | NA |
| SG:LSI_None_Pbo | 0 | 8.30 | NA | 8.30 | NA | NA | NA | NA |
| Tirzepatide:LSI_None_Pbo | 1 | 19.50 | 19.50 | NA | NA | NA | NA | NA |
| OAGB:Orlistat | 0 | 8.74 | NA | 8.74 | NA | NA | NA | NA |
| OAGB:RYGB | 0 | -1.20 | NA | -1.20 | NA | NA | NA | NA |
| OAGB:Semaglutide | 0 | -0.68 | NA | -0.68 | NA | NA | NA | NA |
| OAGB:SG | 1 | 2.70 | 2.70 | NA | NA | NA | NA | NA |
| OAGB:Tirzepatide | 0 | -8.50 | NA | -8.50 | NA | NA | NA | NA |
| Orlistat:RYGB | 0 | -9.94 | NA | -9.94 | NA | NA | NA | NA |
| Orlistat:Semaglutide | 0 | -9.41 | NA | -9.41 | NA | NA | NA | NA |
| Orlistat:SG | 0 | -6.04 | NA | -6.04 | NA | NA | NA | NA |
| Orlistat:Tirzepatide | 0 | -17.24 | NA | -17.24 | NA | NA | NA | NA |
| RYGB:Semaglutide | 0 | 0.52 | NA | 0.52 | NA | NA | NA | NA |
| RYGB:SG | 1 | 3.90 | 3.90 | NA | NA | NA | NA | NA |
| RYGB:Tirzepatide | 0 | -7.30 | NA | -7.30 | NA | NA | NA | NA |
| Semaglutide:SG | 0 | 3.38 | NA | 3.38 | NA | NA | NA | NA |
| Semaglutide:Tirzepatide | 0 | -7.82 | NA | -7.82 | NA | NA | NA | NA |
| SG:Tirzepatide | 0 | -11.20 | NA | -11.20 | NA | NA | NA | NA |

Table 9S – Principal characteristics of the studies and comparisons included in the NMA for TBWL% (class II of obesity).

| **Characteristic** | **Value** |
| --- | --- |
| Number of Interventions | 14 |
| Number of Studies | 56 |
| Total Number of Patients in Network | 28,368 |
| Total Possible Pairwise Comparisons | 91 |
| Total Number of Pairwise Comparisons With Direct Data | 16 |
| Is the network connected? | TRUE |
| Number of Two-arm Studies | 50 |
| Number of Multi-Arms Studies | 3 |
| Average Outcome (TBWL, %) | 7.2 |

# Table 10S – Pairwise comparison table for TBWL% at the endpoint for class II of obesity.

Treatments are ranked from best to worst along the leading diagonal for TBWL% at the endpoint for studies with a mean BMI at entry ranging from 30 to 34.9 kg/m^2^. Above the leading diagonal are estimates from pairwise meta-analyses, below the leading diagonal are estimates from network meta-analyses. Relative treatment effects in ranked order for all studies. Blue boxes: p<0.05.


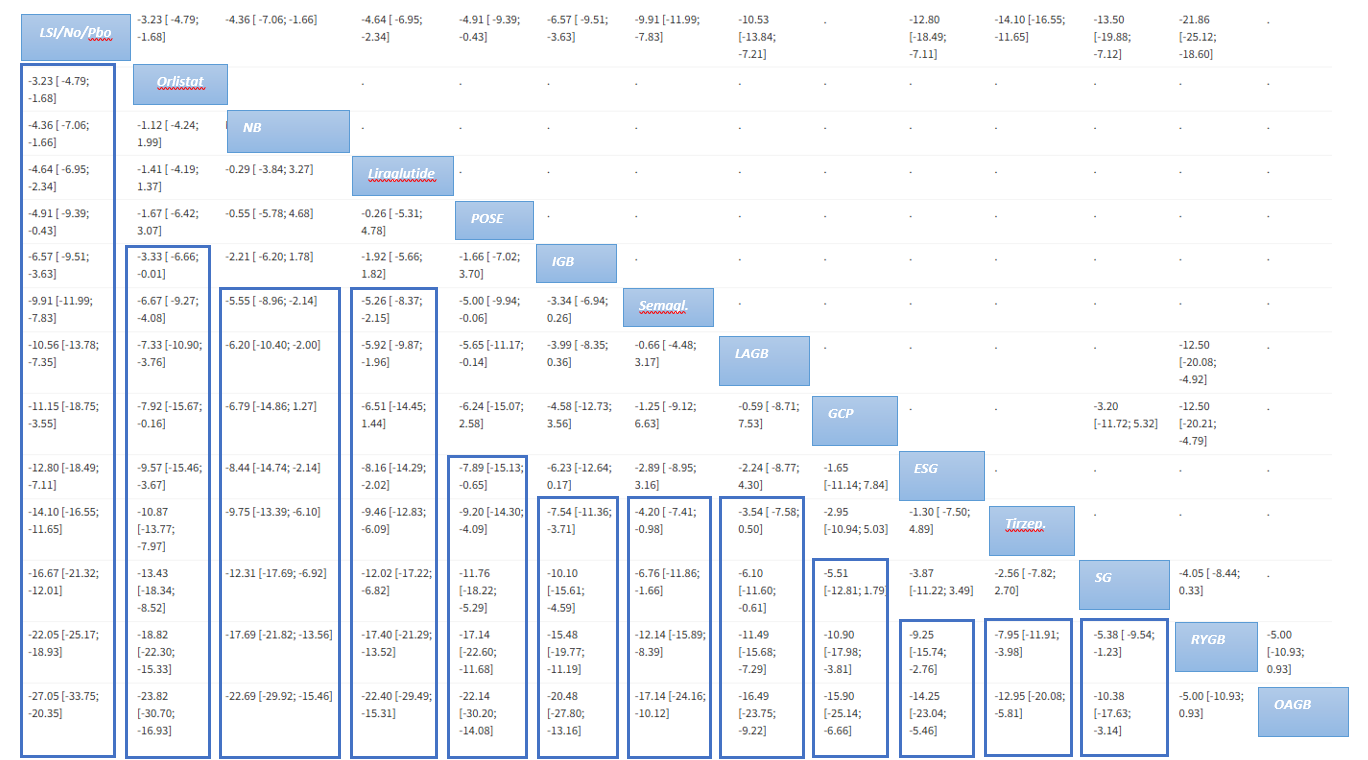


*LSI/No/Pbo: Lifestyle interventions/No therapy/Placebo; IGB: Intragastric Balloon; RYGB: Rou-en-Y Gastric By-pass; OAGB: One-Anastomosis Gastric By-pass; SG: Sleeve Gastrectomy; NB: Naltrexone/Bupropion; Semagl.: Semgalutide; Tirzep.: Tirzepatide; GCP: Greater Curvature Plication; LAGB; Laparoscopic Adjustable Gastric Banding; ESG; Endo Sleeve Gastrectomy; SG: Sleeve Gastrectomy.*

# Table 11S – Assessment of inconsistency for all comparisons for TBWL% at the endpoint in class II of obesity.

# Diff.: difference; CI: Confidence intervals; L/P/None; Lifestyle interventions/Placebo/No interventions. IGB: Intragastric Balloon; RYGB: Rou-en-Y Gastric By-pass; OAGB: One-Anastomosis Gastric By-pass; SG: Sleeve Gastrectomy.

| **Comparison** | **N. Studies** | **NMA** | **Direct** | **Indirect** | **Diff.** | **Diff.**  **95%. CI lower** | **Diff.**  **95%. CI upper** | **p value** |
| --- | --- | --- | --- | --- | --- | --- | --- | --- |
| ESG:GCP | 0 | 1.65 | NA | 1.65 | NA | NA | NA | NA |
| ESG:IGB | 0 | 6.23 | NA | 6.23 | NA | NA | NA | NA |
| ESG:LAGB | 0 | 2.24 | NA | 2.24 | NA | NA | NA | NA |
| ESG:Liraglutide | 0 | 8.16 | NA | 8.16 | NA | NA | NA | NA |
| ESG:LSI_Pbo_None | 1 | 12.80 | 12.80 | NA | NA | NA | NA | NA |
| ESG:Naltrexone_Bupropion | 0 | 8.44 | NA | 8.44 | NA | NA | NA | NA |
| ESG:OAGB | 0 | -14.25 | NA | -14.25 | NA | NA | NA | NA |
| ESG:Orlistat | 0 | 9.57 | NA | 9.57 | NA | NA | NA | NA |
| ESG:POSE | 0 | 7.89 | NA | 7.89 | NA | NA | NA | NA |
| ESG:RYGB | 0 | -9.25 | NA | -9.25 | NA | NA | NA | NA |
| ESG:Semaglutide | 0 | 2.89 | NA | 2.89 | NA | NA | NA | NA |
| ESG:SG | 0 | -3.87 | NA | -3.87 | NA | NA | NA | NA |
| ESG:Tirzepatide | 0 | -1.30 | NA | -1.30 | NA | NA | NA | NA |
| GCP:IGB | 0 | 4.58 | NA | 4.58 | NA | NA | NA | NA |
| GCP:LAGB | 0 | 0.59 | NA | 0.59 | NA | NA | NA | NA |
| GCP:Liraglutide | 0 | 6.51 | NA | 6.51 | NA | NA | NA | NA |
| GCP:LSI_Pbo_None | 0 | 11.15 | NA | 11.15 | NA | NA | NA | NA |
| GCP:Naltrexone_Bupropion | 0 | 6.79 | NA | 6.79 | NA | NA | NA | NA |
| GCP:OAGB | 0 | -15.90 | NA | -15.90 | NA | NA | NA | NA |
| GCP:Orlistat | 0 | 7.92 | NA | 7.92 | NA | NA | NA | NA |
| GCP:POSE | 0 | 6.24 | NA | 6.24 | NA | NA | NA | NA |
| GCP:RYGB | 1 | -10.90 | -12.50 | -2.23 | -10.27 | -29.79 | 9.25 | 0.30 |
| GCP:Semaglutide | 0 | 1.25 | NA | 1.25 | NA | NA | NA | NA |
| GCP:SG | 1 | -5.51 | -3.20 | -11.89 | 8.69 | -7.83 | 25.21 | 0.30 |
| GCP:Tirzepatide | 0 | -2.95 | NA | -2.95 | NA | NA | NA | NA |
| IGB:LAGB | 0 | -3.99 | NA | -3.99 | NA | NA | NA | NA |
| IGB:Liraglutide | 0 | 1.92 | NA | 1.92 | NA | NA | NA | NA |
| IGB:LSI_Pbo_None | 4 | 6.57 | 6.57 | NA | NA | NA | NA | NA |
| IGB:Naltrexone_Bupropion | 0 | 2.21 | NA | 2.21 | NA | NA | NA | NA |
| IGB:OAGB | 0 | -20.48 | NA | -20.48 | NA | NA | NA | NA |
| IGB:Orlistat | 0 | 3.33 | NA | 3.33 | NA | NA | NA | NA |
| IGB:POSE | 0 | 1.66 | NA | 1.66 | NA | NA | NA | NA |
| IGB:RYGB | 0 | -15.48 | NA | -15.48 | NA | NA | NA | NA |
| IGB:Semaglutide | 0 | -3.34 | NA | -3.34 | NA | NA | NA | NA |
| IGB:SG | 0 | -10.10 | NA | -10.10 | NA | NA | NA | NA |
| IGB:Tirzepatide | 0 | -7.54 | NA | -7.54 | NA | NA | NA | NA |
| LAGB:Liraglutide | 0 | 5.92 | NA | 5.92 | NA | NA | NA | NA |
| LAGB:LSI_Pbo_None | 4 | 10.56 | 10.53 | 11.13 | -0.60 | -14.30 | 13.10 | 0.93 |
| LAGB:Naltrexone_Bupropion | 0 | 6.20 | NA | 6.20 | NA | NA | NA | NA |
| LAGB:OAGB | 0 | -16.49 | NA | -16.49 | NA | NA | NA | NA |
| LAGB:Orlistat | 0 | 7.33 | NA | 7.33 | NA | NA | NA | NA |
| LAGB:POSE | 0 | 5.65 | NA | 5.65 | NA | NA | NA | NA |
| LAGB:RYGB | 1 | -11.49 | -12.50 | -11.04 | -1.46 | -10.56 | 7.64 | 0.75 |
| LAGB:Semaglutide | 0 | 0.66 | NA | 0.66 | NA | NA | NA | NA |
| LAGB:SG | 0 | -6.10 | NA | -6.10 | NA | NA | NA | NA |
| LAGB:Tirzepatide | 0 | -3.54 | NA | -3.54 | NA | NA | NA | NA |
| Liraglutide:LSI_Pbo_None | 6 | 4.64 | 4.64 | NA | NA | NA | NA | NA |
| Liraglutide:Naltrexone_Bupropion | 0 | 0.29 | NA | 0.29 | NA | NA | NA | NA |
| Liraglutide:OAGB | 0 | -22.40 | NA | -22.40 | NA | NA | NA | NA |
| Liraglutide:Orlistat | 0 | 1.41 | NA | 1.41 | NA | NA | NA | NA |
| Liraglutide:POSE | 0 | -0.26 | NA | -0.26 | NA | NA | NA | NA |
| Liraglutide:RYGB | 0 | -17.40 | NA | -17.40 | NA | NA | NA | NA |
| Liraglutide:Semaglutide | 0 | -5.26 | NA | -5.26 | NA | NA | NA | NA |
| Liraglutide:SG | 0 | -12.02 | NA | -12.02 | NA | NA | NA | NA |
| Liraglutide:Tirzepatide | 0 | -9.46 | NA | -9.46 | NA | NA | NA | NA |
| Naltrexone_Bupropion:LSI_Pbo_None | 4 | 4.36 | 4.36 | NA | NA | NA | NA | NA |
| OAGB:LSI_Pbo_None | 0 | 27.05 | NA | 27.05 | NA | NA | NA | NA |
| Orlistat:LSI_Pbo_None | 14 | 3.23 | 3.23 | NA | NA | NA | NA | NA |
| POSE:LSI_Pbo_None | 2 | 4.91 | 4.91 | NA | NA | NA | NA | NA |
| RYGB:LSI_Pbo_None | 4 | 22.05 | 21.86 | 24.08 | -2.22 | -13.45 | 9.01 | 0.70 |
| Semaglutide:LSI_Pbo_None | 7 | 9.91 | 9.91 | NA | NA | NA | NA | NA |
| SG:LSI_Pbo_None | 1 | 16.67 | 13.50 | 20.27 | -6.77 | -16.11 | 2.57 | 0.16 |
| Tirzepatide:LSI_Pbo_None | 5 | 14.10 | 14.10 | NA | NA | NA | NA | NA |
| Naltrexone_Bupropion:OAGB | 0 | -22.69 | NA | -22.69 | NA | NA | NA | NA |
| Naltrexone_Bupropion:Orlistat | 0 | 1.12 | NA | 1.12 | NA | NA | NA | NA |
| Naltrexone_Bupropion:POSE | 0 | -0.55 | NA | -0.55 | NA | NA | NA | NA |
| Naltrexone_Bupropion:RYGB | 0 | -17.69 | NA | -17.69 | NA | NA | NA | NA |
| Naltrexone_Bupropion:Semaglutide | 0 | -5.55 | NA | -5.55 | NA | NA | NA | NA |
| Naltrexone_Bupropion:SG | 0 | -12.31 | NA | -12.31 | NA | NA | NA | NA |
| Naltrexone_Bupropion:Tirzepatide | 0 | -9.75 | NA | -9.75 | NA | NA | NA | NA |
| OAGB:Orlistat | 0 | 23.82 | NA | 23.82 | NA | NA | NA | NA |
| OAGB:POSE | 0 | 22.14 | NA | 22.14 | NA | NA | NA | NA |
| OAGB:RYGB | 1 | 5.00 | 5.00 | NA | NA | NA | NA | NA |
| OAGB:Semaglutide | 0 | 17.14 | NA | 17.14 | NA | NA | NA | NA |
| OAGB:SG | 0 | 10.38 | NA | 10.38 | NA | NA | NA | NA |
| OAGB:Tirzepatide | 0 | 12.95 | NA | 12.95 | NA | NA | NA | NA |
| Orlistat:POSE | 0 | -1.67 | NA | -1.67 | NA | NA | NA | NA |
| Orlistat:RYGB | 0 | -18.82 | NA | -18.82 | NA | NA | NA | NA |
| Orlistat:Semaglutide | 0 | -6.67 | NA | -6.67 | NA | NA | NA | NA |
| Orlistat:SG | 0 | -13.43 | NA | -13.43 | NA | NA | NA | NA |
| Orlistat:Tirzepatide | 0 | -10.87 | NA | -10.87 | NA | NA | NA | NA |
| POSE:RYGB | 0 | -17.14 | NA | -17.14 | NA | NA | NA | NA |
| POSE:Semaglutide | 0 | -5.00 | NA | -5.00 | NA | NA | NA | NA |
| POSE:SG | 0 | -11.76 | NA | -11.76 | NA | NA | NA | NA |
| POSE:Tirzepatide | 0 | -9.20 | NA | -9.20 | NA | NA | NA | NA |
| RYGB:Semaglutide | 0 | 12.14 | NA | 12.14 | NA | NA | NA | NA |
| RYGB:SG | 3 | 5.38 | 4.05 | 16.70 | -12.65 | -26.19 | 0.89 | 0.07 |
| RYGB:Tirzepatide | 0 | 7.95 | NA | 7.95 | NA | NA | NA | NA |
| Semaglutide:SG | 0 | -6.76 | NA | -6.76 | NA | NA | NA | NA |
| Semaglutide:Tirzepatide | 0 | -4.20 | NA | -4.20 | NA | NA | NA | NA |
| SG:Tirzepatide | 0 | 2.56 | NA | 2.56 | NA | NA | NA | NA |

Table 12S – Principal characteristics of the studies and comparisons included in the NMA for TBWL% (class III of obesity).

| **Characteristic** | **Value** |
| --- | --- |
| Number of Interventions | 9 |
| Number of Studies | 47 |
| Total Number of Patients in Network | 5,991 |
| Total Possible Pairwise Comparisons | 36 |
| Total Number of Pairwise Comparisons With Direct Data | 12 |
| Is the network connected? | TRUE |
| Number of Two-arm Studies | 44 |
| Number of Multi-Arms Studies | 3 |
| Average Outcome (TBWL, %) | 25.8 |

# Table 13S – Pairwise comparison table for TBWL% at the endpoint for class III of obesity.

Treatments are ranked from best to worst along the leading diagonal for TBWL% at the endpoint for studies with a mean BMI at entry ranging from 30 to 34.9 kg/m^2^. Above the leading diagonal are estimates from pairwise meta-analyses, below the leading diagonal are estimates from network meta-analyses. Relative treatment effects in ranked order for all studies. Blue boxes: p<0.05.


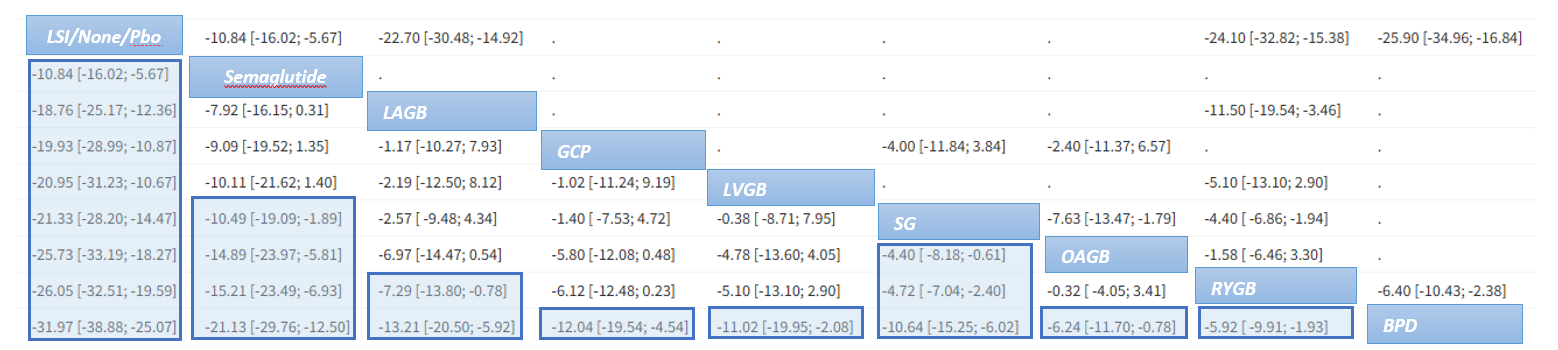


*LSI/No/Pbo: Lifestyle interventions/No therapy/Placebo; IGB: Intragastric Balloon; RYGB: Rou-en-Y Gastric By-pass; OAGB: One-Anastomosis Gastric By-pass; SG: Sleeve Gastrectomy; GCP: Greater Curvature Plication; LAGB; Laparoscopic Adjustable Gastric Banding; ESG; Endo Sleeve Gastrectomy; SG: Sleeve Gastrectomy; BPD: Bili-Pancreatic Diversion.*

# Table 14S – Assessment of inconsistency for all comparisons for TBWL% at the endpoint in class III of obesity.

Diff.: difference; CI: Confidence intervals; L/P/None; Lifestyle interventions/Placebo/No interventions. IGB: Intragastric Balloon; RYGB: Rou-en-Y Gastric By-pass; OAGB: One-Anastomosis Gastric By-pass; SG: Sleeve Gastrectomy.

| **Comparison** | **N. Studies** | **NMA** | **Direct** | **Indirect** | **Diff.** | **Diff.**  **95%. CI lower** | **Diff.**  **95%. CI upper** | **p value** |
| --- | --- | --- | --- | --- | --- | --- | --- | --- |
| BPD:GCP | 0 | 12.04 | NA | 12.04 | NA | NA | NA | NA |
| BPD:LAGB | 0 | 13.21 | NA | 13.21 | NA | NA | NA | NA |
| BPD:LSI_Pbo_None | 1 | 31.97 | 25.90 | 40.38 | -14.48 | -28.47 | -0.48 | 0.04 |
| BPD:LVBG | 0 | 11.02 | NA | 11.02 | NA | NA | NA | NA |
| BPD:OAGB | 0 | 6.24 | NA | 6.24 | NA | NA | NA | NA |
| BPD:RYGB | 4 | 5.92 | 6.40 | -20.09 | 26.49 | -3.20 | 56.19 | 0.08 |
| BPD:Semaglutide | 0 | 21.13 | NA | 21.13 | NA | NA | NA | NA |
| BPD:SG | 0 | 10.64 | NA | 10.64 | NA | NA | NA | NA |
| GCP:LAGB | 0 | 1.17 | NA | 1.17 | NA | NA | NA | NA |
| GCP:LSI_Pbo_None | 0 | 19.93 | NA | 19.93 | NA | NA | NA | NA |
| GCP:LVBG | 0 | -1.02 | NA | -1.02 | NA | NA | NA | NA |
| GCP:OAGB | 1 | -5.80 | -2.40 | -9.07 | 6.67 | -5.89 | 19.23 | 0.30 |
| GCP:RYGB | 0 | -6.12 | NA | -6.12 | NA | NA | NA | NA |
| GCP:Semaglutide | 0 | 9.09 | NA | 9.09 | NA | NA | NA | NA |
| GCP:SG | 1 | -1.40 | -4.00 | 2.67 | -6.67 | -19.23 | 5.89 | 0.30 |
| LAGB:LSI_Pbo_None | 1 | 18.76 | 22.70 | 10.47 | 12.23 | -1.48 | 25.93 | 0.08 |
| LAGB:LVBG | 0 | -2.19 | NA | -2.19 | NA | NA | NA | NA |
| LAGB:OAGB | 0 | -6.97 | NA | -6.97 | NA | NA | NA | NA |
| LAGB:RYGB | 1 | -7.29 | -11.50 | 0.73 | -12.23 | -25.93 | 1.48 | 0.08 |
| LAGB:Semaglutide | 0 | 7.92 | NA | 7.92 | NA | NA | NA | NA |
| LAGB:SG | 0 | -2.57 | NA | -2.57 | NA | NA | NA | NA |
| LVBG:LSI_Pbo_None | 0 | 20.95 | NA | 20.95 | NA | NA | NA | NA |
| OAGB:LSI_Pbo_None | 0 | 25.73 | NA | 25.73 | NA | NA | NA | NA |
| RYGB:LSI_Pbo_None | 1 | 26.05 | 24.10 | 28.43 | -4.33 | -17.31 | 8.66 | 0.51 |
| Semaglutide:LSI_Pbo_None | 2 | 10.84 | 10.84 | NA | NA | NA | NA | NA |
| SG:LSI_Pbo_None | 0 | 21.33 | NA | 21.33 | NA | NA | NA | NA |
| LVBG:OAGB | 0 | -4.78 | NA | -4.78 | NA | NA | NA | NA |
| LVBG:RYGB | 1 | -5.10 | -5.10 | NA | NA | NA | NA | NA |
| LVBG:Semaglutide | 0 | 10.11 | NA | 10.11 | NA | NA | NA | NA |
| LVBG:SG | 0 | -0.38 | NA | -0.38 | NA | NA | NA | NA |
| OAGB:RYGB | 3 | -0.32 | -1.58 | 1.44 | -3.02 | -10.59 | 4.55 | 0.43 |
| OAGB:Semaglutide | 0 | 14.89 | NA | 14.89 | NA | NA | NA | NA |
| OAGB:SG | 2 | 4.40 | 7.63 | 2.06 | 5.57 | -2.09 | 13.24 | 0.15 |
| RYGB:Semaglutide | 0 | 15.21 | NA | 15.21 | NA | NA | NA | NA |
| RYGB:SG | 11 | 4.72 | 4.40 | 7.42 | -3.02 | -10.59 | 4.55 | 0.43 |
| Semaglutide:SG | 0 | -10.49 | NA | -10.49 | NA | NA | NA | NA |

# Table 12S – GRADE evaluation of retrieved evidence for the primary endpoint (i.e., TBWL% at the endpoint) for studies versus LSI/Pbo/No therapy.

| **Certainty assessment** | | | | | | | **Summary of findings** | | | | |
| --- | --- | --- | --- | --- | --- | --- | --- | --- | --- | --- | --- |
| **Participants (studies) Follow-up** | **Risk of bias** | **Inconsistency** | **Indirectness** | **Imprecision** | **Publication bias** | **Overall certainty of evidence** | **Study event rates (%)** | | **Relative effect (95% CI)** | **Anticipated absolute effects** | |
|  |  |  |  |  |  |  | **With PBO** | **With AOS** |  | **Risk with PBO** | **Risk difference with TBWL%** |
| **TBWL% endpoint**  **BMI at entry 30-34.9 kg/m^2^** | | | | | | | | | | | |
| 21,268 (14 RCTs) | serious a | serious b | not serious | not serious | strong association | **⨁⨁⨁◯ Moderate^a,b^** | 10,430 | 10,838 | - | - | WMD **7.53** higher (4.97 higher to 10.09 higher) |

**BMI at entry 35-39.9 kg/m^2^**

| 31,110 (64 RCTs) | serious ^a^ | serious ^b^ | not serious | not serious | strong association | **⨁⨁⨁◯ Moderate^a,b^** | 13,285 | 17,825 | - | - | MD **7.75 higher** (6.38 higher to 9.11 higher) |
| --- | --- | --- | --- | --- | --- | --- | --- | --- | --- | --- | --- |

**BMI at entry >39.9 kg/m^2^**

| 754 (5 RCTs) | serious ^a^ | serious ^b^ | not serious | serious^c^ | very strong association | **⨁⨁⨁◯ Moderate^a,b,c^** | 275 | 479 | - | - | WMD **18.37 higher**  (13.23 higher to 23.5 higher) |
| --- | --- | --- | --- | --- | --- | --- | --- | --- | --- | --- | --- |

**CI:** confidence interval; **WMD:** weighted mean difference; **AOS**: Anti-obesity strategy.

**Explanations:** a. Open-label studies. b. High heterogeneity (I^2^>50%); c. Small sample size.

# Table 13S – GRADE evaluation of retrieved evidence for the secondary endpoints with at least 10 studies versus LSI/Pbo/No therapy.

| **Certainty assessment** | | | | | | | **Summary of findings** | | | | |
| --- | --- | --- | --- | --- | --- | --- | --- | --- | --- | --- | --- |
| **Participants (studies) Follow-up** | **Risk of bias** | **Inconsistency** | **Indirectness** | **Imprecision** | **Publication bias** | **Overall certainty of evidence** | **Study event rates (%)** | | **Relative effect (95% CI)** | **Anticipated absolute effects** | |
|  |  |  |  |  |  |  | **With PBO** | **With AOS** |  | **Risk with PBO** | **Risk difference with TBWL%** |
| **Serious adverse events**  **BMI at entry 30-34.9 kg/m^2^** | | | | | | | | | | | |
| 10,236 (10 RCTs) | not serious | serious^b^ | not serious | not serious | none | **⨁⨁⨁◯ Moderate^b^** | 3,239/4,969 | 3,017/5,267 | 0.94 [0.46, 1.95] | - | - |

**BMI at entry 35-39.9 kg/m^2^**

| 35,060 (59 RCTs) | serious^a^ | serious^b^ | not serious | not serious | strong association | **⨁⨁⨁◯ Moderate**^a,^**^b^** | 1,421/15,950 | 1,861/19,110 | 1.20 [1.03, 1.41] | - | - |
| --- | --- | --- | --- | --- | --- | --- | --- | --- | --- | --- | --- |

**CI:** confidence interval; **WMD:** weighted mean difference; **AOS**: Anti-obesity strategy.

**Explanations:** a. Open-label studies. b. High heterogeneity (I^2^>50%); c. Small sample size.

|  |  |  |  |
| --- | --- | --- | --- |
|  |  |  |  |

# References

1. Astrup A, Carraro R, Finer N, Harper A, Kunesova M, Lean ME, et al. Safety, tolerability and sustained weight loss over 2 years with the once-daily human GLP-1 analog, liraglutide. Int J Obes (Lond) 2012;36(6):843-54. (In eng). DOI: 10.1038/ijo.2011.158.

2. Gudbergsen H, Overgaard A, Henriksen M, Wæhrens EE, Bliddal H, Christensen R, et al. Liraglutide after diet-induced weight loss for pain and weight control in knee osteoarthritis: a randomized controlled trial. Am J Clin Nutr 2021;113(2):314-323. (In eng). DOI: 10.1093/ajcn/nqaa328.

3. Lundgren JR, Janus C, Jensen SBK, Juhl CR, Olsen LM, Christensen RM, et al. Healthy Weight Loss Maintenance with Exercise, Liraglutide, or Both Combined. N Engl J Med 2021;384(18):1719-1730. (In eng). DOI: 10.1056/NEJMoa2028198.

4. O'Neil PM, Birkenfeld AL, McGowan B, Mosenzon O, Pedersen SD, Wharton S, et al. Efficacy and safety of semaglutide compared with liraglutide and placebo for weight loss in patients with obesity: a randomised, double-blind, placebo and active controlled, dose-ranging, phase 2 trial. Lancet 2018;392(10148):637-649. (In eng). DOI: 10.1016/s0140-6736(18)31773-2.

5. Davies MJ, Bergenstal R, Bode B, Kushner RF, Lewin A, Skjøth TV, et al. Efficacy of Liraglutide for Weight Loss Among Patients With Type 2 Diabetes: The SCALE Diabetes Randomized Clinical Trial. Jama 2015;314(7):687-99. (In eng). DOI: 10.1001/jama.2015.9676.

6. Garvey WT, Birkenfeld AL, Dicker D, Mingrone G, Pedersen SD, Satylganova A, et al. Efficacy and Safety of Liraglutide 3.0 mg in Individuals With Overweight or Obesity and Type 2 Diabetes Treated With Basal Insulin: The SCALE Insulin Randomized Controlled Trial. Diabetes Care 2020;43(5):1085-1093. (In eng). DOI: 10.2337/dc19-1745.

7. Wadden TA, Hollander P, Klein S, Niswender K, Woo V, Hale PM, et al. Weight maintenance and additional weight loss with liraglutide after low-calorie-diet-induced weight loss: the SCALE Maintenance randomized study. Int J Obes (Lond) 2013;37(11):1443-51. (In eng). DOI: 10.1038/ijo.2013.120.

8. Wadden TA, Tronieri JS, Sugimoto D, Lund MT, Auerbach P, Jensen C, et al. Liraglutide 3.0 mg and Intensive Behavioral Therapy (IBT) for Obesity in Primary Care: The SCALE IBT Randomized Controlled Trial. Obesity (Silver Spring) 2020;28(3):529-536. (In eng). DOI: 10.1002/oby.22726.

9. le Roux CW, Astrup A, Fujioka K, Greenway F, Lau DCW, Van Gaal L, et al. 3 years of liraglutide versus placebo for type 2 diabetes risk reduction and weight management in individuals with prediabetes: a randomised, double-blind trial. Lancet 2017;389(10077):1399-1409. (In eng). DOI: 10.1016/s0140-6736(17)30069-7.

10. Apovian CM, Aronne L, Rubino D, Still C, Wyatt H, Burns C, et al. A randomized, phase 3 trial of naltrexone SR/bupropion SR on weight and obesity-related risk factors (COR-II). Obesity (Silver Spring) 2013;21(5):935-43. (In eng). DOI: 10.1002/oby.20309.

11. Greenway FL, Fujioka K, Plodkowski RA, Mudaliar S, Guttadauria M, Erickson J, et al. Effect of naltrexone plus bupropion on weight loss in overweight and obese adults (COR-I): a multicentre, randomised, double-blind, placebo-controlled, phase 3 trial. Lancet 2010;376(9741):595-605. (In eng). DOI: 10.1016/s0140-6736(10)60888-4.

12. Hollander P, Gupta AK, Plodkowski R, Greenway F, Bays H, Burns C, et al. Effects of naltrexone sustained-release/bupropion sustained-release combination therapy on body weight and glycemic parameters in overweight and obese patients with type 2 diabetes. Diabetes Care 2013;36(12):4022-9. (In eng). DOI: 10.2337/dc13-0234.

13. Wadden TA, Foreyt JP, Foster GD, Hill JO, Klein S, O'Neil PM, et al. Weight loss with naltrexone SR/bupropion SR combination therapy as an adjunct to behavior modification: the COR-BMOD trial. Obesity (Silver Spring) 2011;19(1):110-20. (In eng). DOI: 10.1038/oby.2010.147.

14. Nissen SE, Wolski KE, Prcela L, Wadden T, Buse JB, Bakris G, et al. Effect of Naltrexone-Bupropion on Major Adverse Cardiovascular Events in Overweight and Obese Patients With Cardiovascular Risk Factors: A Randomized Clinical Trial. Jama 2016;315(10):990-1004. (In eng). DOI: 10.1001/jama.2016.1558.

15. Bakris G, Calhoun D, Egan B, Hellmann C, Dolker M, Kingma I. Orlistat improves blood pressure control in obese subjects with treated but inadequately controlled hypertension. J Hypertens 2002;20(11):2257-67. (In eng). DOI: 10.1097/00004872-200211000-00026.

16. Berne C. A randomized study of orlistat in combination with a weight management programme in obese patients with Type 2 diabetes treated with metformin. Diabet Med 2005;22(5):612-8. (In eng). DOI: 10.1111/j.1464-5491.2004.01474.x.

17. Davidson MH, Hauptman J, DiGirolamo M, Foreyt JP, Halsted CH, Heber D, et al. Weight control and risk factor reduction in obese subjects treated for 2 years with orlistat: a randomized controlled trial. Jama 1999;281(3):235-42. (In eng). DOI: 10.1001/jama.281.3.235.

18. Derosa G, Cicero AF, D'Angelo A, Fogari E, Maffioli P. Effects of 1-year orlistat treatment compared to placebo on insulin resistance parameters in patients with type 2 diabetes. J Clin Pharm Ther 2012;37(2):187-95. (In eng). DOI: 10.1111/j.1365-2710.2011.01280.x.

19. Derosa G, Mugellini A, Ciccarelli L, Fogari R. Randomized, double-blind, placebo-controlled comparison of the action of orlistat, fluvastatin, or both an anthropometric measurements, blood pressure, and lipid profile in obese patients with hypercholesterolemia prescribed a standardized diet. Clin Ther 2003;25(4):1107-22. (In eng). DOI: 10.1016/s0149-2918(03)80070-x.

20. Finer N, James WP, Kopelman PG, Lean ME, Williams G. One-year treatment of obesity: a randomized, double-blind, placebo-controlled, multicentre study of orlistat, a gastrointestinal lipase inhibitor. Int J Obes Relat Metab Disord 2000;24(3):306-13. (In eng). DOI: 10.1038/sj.ijo.0801128.

21. Hill JO, Hauptman J, Anderson JW, Fujioka K, O'Neil PM, Smith DK, et al. Orlistat, a lipase inhibitor, for weight maintenance after conventional dieting: a 1-y study. Am J Clin Nutr 1999;69(6):1108-16. (In eng). DOI: 10.1093/ajcn/69.6.1108.

22. James WP, Avenell A, Broom J, Whitehead J. A one-year trial to assess the value of orlistat in the management of obesity. Int J Obes Relat Metab Disord 1997;21 Suppl 3:S24-30. (In eng).

23. Karhunen L, Franssila-Kallunki A, Rissanen P, Valve R, Kolehmainen M, Rissanen A, et al. Effect of orlistat treatment on body composition and resting energy expenditure during a two-year weight-reduction programme in obese Finns. Int J Obes Relat Metab Disord 2000;24(12):1567-72. (In eng). DOI: 10.1038/sj.ijo.0801443.

24. Kelley DE, Bray GA, Pi-Sunyer FX, Klein S, Hill J, Miles J, et al. Clinical efficacy of orlistat therapy in overweight and obese patients with insulin-treated type 2 diabetes: A 1-year randomized controlled trial. Diabetes Care 2002;25(6):1033-41. (In eng). DOI: 10.2337/diacare.25.6.1033.

25. Miles JM, Leiter L, Hollander P, Wadden T, Anderson JW, Doyle M, et al. Effect of orlistat in overweight and obese patients with type 2 diabetes treated with metformin. Diabetes Care 2002;25(7):1123-8. (In eng). DOI: 10.2337/diacare.25.7.1123.

26. Poston WS, Reeves RS, Haddock CK, Stormer S, Balasubramanyam A, Satterwhite O, et al. Weight loss in obese Mexican Americans treated for 1-year with orlistat and lifestyle modification. Int J Obes Relat Metab Disord 2003;27(12):1486-93. (In eng). DOI: 10.1038/sj.ijo.0802439.

27. Sjöström L, Rissanen A, Andersen T, Boldrin M, Golay A, Koppeschaar HP, et al. Randomised placebo-controlled trial of orlistat for weight loss and prevention of weight regain in obese patients. European Multicentre Orlistat Study Group. Lancet 1998;352(9123):167-72. (In eng). DOI: 10.1016/s0140-6736(97)11509-4.

28. Svendsen M, Helgeland M, Tonstad S. The long-term influence of orlistat on dietary intake in obese subjects with components of metabolic syndrome. J Hum Nutr Diet 2009;22(1):55-63. (In eng). DOI: 10.1111/j.1365-277X.2008.00920.x.

29. Swinburn BA, Carey D, Hills AP, Hooper M, Marks S, Proietto J, et al. Effect of orlistat on cardiovascular disease risk in obese adults. Diabetes, obesity & metabolism 2005;7(3):254-62. (In eng). DOI: 10.1111/j.1463-1326.2004.00467.x.

30. Zavoral JH. Treatment with orlistat reduces cardiovascular risk in obese patients. J Hypertens 1998;16(12 Pt 2):2013-7. (In eng). DOI: 10.1097/00004872-199816121-00024.

31. Hollander PA, Elbein SC, Hirsch IB, Kelley D, McGill J, Taylor T, et al. Role of orlistat in the treatment of obese patients with type 2 diabetes. A 1-year randomized double-blind study. Diabetes Care 1998;21(8):1288-94. (In eng). DOI: 10.2337/diacare.21.8.1288.

32. Krempf M, Louvet JP, Allanic H, Miloradovich T, Joubert JM, Attali JR. Weight reduction and long-term maintenance after 18 months treatment with orlistat for obesity. Int J Obes Relat Metab Disord 2003;27(5):591-7. (In eng). DOI: 10.1038/sj.ijo.0802281.

33. Hauptman J, Lucas C, Boldrin MN, Collins H, Segal KR. Orlistat in the long-term treatment of obesity in primary care settings. Arch Fam Med 2000;9(2):160-7. (In eng). DOI: 10.1001/archfami.9.2.160.

34. Rössner S, Sjöström L, Noack R, Meinders AE, Noseda G. Weight loss, weight maintenance, and improved cardiovascular risk factors after 2 years treatment with orlistat for obesity. European Orlistat Obesity Study Group. Obes Res 2000;8(1):49-61. (In eng). DOI: 10.1038/oby.2000.8.

35. Richelsen B, Tonstad S, Rössner S, Toubro S, Niskanen L, Madsbad S, et al. Effect of orlistat on weight regain and cardiovascular risk factors following a very-low-energy diet in abdominally obese patients: a 3-year randomized, placebo-controlled study. Diabetes Care 2007;30(1):27-32. (In eng). DOI: 10.2337/dc06-0210.

36. Torgerson JS, Hauptman J, Boldrin MN, Sjöström L. XENical in the prevention of diabetes in obese subjects (XENDOS) study: a randomized study of orlistat as an adjunct to lifestyle changes for the prevention of type 2 diabetes in obese patients. Diabetes Care 2004;27(1):155-61. (In eng). DOI: 10.2337/diacare.27.1.155.

37. Allison DB, Gadde KM, Garvey WT, Peterson CA, Schwiers ML, Najarian T, et al. Controlled-release phentermine/topiramate in severely obese adults: a randomized controlled trial (EQUIP). Obesity (Silver Spring) 2012;20(2):330-42. (In eng). DOI: 10.1038/oby.2011.330.

38. Gadde KM, Allison DB, Ryan DH, Peterson CA, Troupin B, Schwiers ML, et al. Effects of low-dose, controlled-release, phentermine plus topiramate combination on weight and associated comorbidities in overweight and obese adults (CONQUER): a randomised, placebo-controlled, phase 3 trial. Lancet 2011;377(9774):1341-52. (In eng). DOI: 10.1016/s0140-6736(11)60205-5.

39. Kosiborod MN, Petrie MC, Borlaug BA, Butler J, Davies MJ, Hovingh GK, et al. Semaglutide in Patients with Obesity-Related Heart Failure and Type 2 Diabetes. N Engl J Med 2024;390(15):1394-1407. (In eng). DOI: 10.1056/NEJMoa2313917.

40. Kosiborod MN, Abildstrøm SZ, Borlaug BA, Butler J, Rasmussen S, Davies M, et al. Semaglutide in Patients with Heart Failure with Preserved Ejection Fraction and Obesity. N Engl J Med 2023;389(12):1069-1084. (In eng). DOI: 10.1056/NEJMoa2306963.

41. McGowan BM, Bruun JM, Capehorn M, Pedersen SD, Pietiläinen KH, Muniraju HAK, et al. Efficacy and safety of once-weekly semaglutide 2·4 mg versus placebo in people with obesity and prediabetes (STEP 10): a randomised, double-blind, placebo-controlled, multicentre phase 3 trial. Lancet Diabetes Endocrinol 2024;12(9):631-642. (In eng). DOI: 10.1016/s2213-8587(24)00182-7.

42. Bliddal H, Bays H, Czernichow S, Uddén Hemmingsson J, Hjelmesæth J, Hoffmann Morville T, et al. Once-Weekly Semaglutide in Persons with Obesity and Knee Osteoarthritis. N Engl J Med 2024;391(17):1573-1583. (In eng). DOI: 10.1056/NEJMoa2403664.

43. Davies M, Færch L, Jeppesen OK, Pakseresht A, Pedersen SD, Perreault L, et al. Semaglutide 2·4 mg once a week in adults with overweight or obesity, and type 2 diabetes (STEP 2): a randomised, double-blind, double-dummy, placebo-controlled, phase 3 trial. Lancet 2021;397(10278):971-984. (In eng). DOI: 10.1016/s0140-6736(21)00213-0.

44. Kadowaki T, Isendahl J, Khalid U, Lee SY, Nishida T, Ogawa W, et al. Semaglutide once a week in adults with overweight or obesity, with or without type 2 diabetes in an east Asian population (STEP 6): a randomised, double-blind, double-dummy, placebo-controlled, phase 3a trial. Lancet Diabetes Endocrinol 2022;10(3):193-206. (In eng). DOI: 10.1016/s2213-8587(22)00008-0.

45. Rubino D, Abrahamsson N, Davies M, Hesse D, Greenway FL, Jensen C, et al. Effect of Continued Weekly Subcutaneous Semaglutide vs Placebo on Weight Loss Maintenance in Adults With Overweight or Obesity: The STEP 4 Randomized Clinical Trial. Jama 2021;325(14):1414-1425. (In eng). DOI: 10.1001/jama.2021.3224.

46. Wadden TA, Bailey TS, Billings LK, Davies M, Frias JP, Koroleva A, et al. Effect of Subcutaneous Semaglutide vs Placebo as an Adjunct to Intensive Behavioral Therapy on Body Weight in Adults With Overweight or Obesity: The STEP 3 Randomized Clinical Trial. Jama 2021;325(14):1403-1413. (In eng). DOI: 10.1001/jama.2021.1831.

47. Wilding JPH, Batterham RL, Calanna S, Davies M, Van Gaal LF, Lingvay I, et al. Once-Weekly Semaglutide in Adults with Overweight or Obesity. N Engl J Med 2021;384(11):989-1002. (In eng). DOI: 10.1056/NEJMoa2032183.

48. Garvey WT, Batterham RL, Bhatta M, Buscemi S, Christensen LN, Frias JP, et al. Two-year effects of semaglutide in adults with overweight or obesity: the STEP 5 trial. Nat Med 2022;28(10):2083-2091. (In eng). DOI: 10.1038/s41591-022-02026-4.

49. Lincoff AM, Brown-Frandsen K, Colhoun HM, Deanfield J, Emerson SS, Esbjerg S, et al. Semaglutide and Cardiovascular Outcomes in Obesity without Diabetes. N Engl J Med 2023;389(24):2221-2232. (In eng). DOI: 10.1056/NEJMoa2307563.

50. Aronne LJ, Sattar N, Horn DB, Bays HE, Wharton S, Lin WY, et al. Continued Treatment With Tirzepatide for Maintenance of Weight Reduction in Adults With Obesity: The SURMOUNT-4 Randomized Clinical Trial. Jama 2024;331(1):38-48. (In eng). DOI: 10.1001/jama.2023.24945.

51. Loomba R, Hartman ML, Lawitz EJ, Vuppalanchi R, Boursier J, Bugianesi E, et al. Tirzepatide for Metabolic Dysfunction-Associated Steatohepatitis with Liver Fibrosis. N Engl J Med 2024;391(4):299-310. (In eng). DOI: 10.1056/NEJMoa2401943.

52. Malhotra A, Grunstein RR, Fietze I, Weaver TE, Redline S, Azarbarzin A, et al. Tirzepatide for the Treatment of Obstructive Sleep Apnea and Obesity. N Engl J Med 2024 (In eng). DOI: 10.1056/NEJMoa2404881.

53. Garvey WT, Frias JP, Jastreboff AM, le Roux CW, Sattar N, Aizenberg D, et al. Tirzepatide once weekly for the treatment of obesity in people with type 2 diabetes (SURMOUNT-2): a double-blind, randomised, multicentre, placebo-controlled, phase 3 trial. Lancet 2023;402(10402):613-626. (In eng). DOI: 10.1016/s0140-6736(23)01200-x.

54. Jastreboff AM, Aronne LJ, Ahmad NN, Wharton S, Connery L, Alves B, et al. Tirzepatide Once Weekly for the Treatment of Obesity. N Engl J Med 2022;387(3):205-216. (In eng). DOI: 10.1056/NEJMoa2206038.

55. Sullivan S, Stein R, Jonnalagadda S, Mullady D, Edmundowicz S. Aspiration therapy leads to weight loss in obese subjects: a pilot study. Gastroenterology 2013;145(6):1245-52.e1-5. (In eng). DOI: 10.1053/j.gastro.2013.08.056.

56. Thompson CC, Abu Dayyeh BK, Kushner R, Sullivan S, Schorr AB, Amaro A, et al. Percutaneous Gastrostomy Device for the Treatment of Class II and Class III Obesity: Results of a Randomized Controlled Trial. Am J Gastroenterol 2017;112(3):447-457. (In eng). DOI: 10.1038/ajg.2016.500.

57. Glaysher MA, Mohanaruban A, Prechtl CG, Goldstone AP, Miras AD, Lord J, et al. A randomised controlled trial of a duodenal-jejunal bypass sleeve device (EndoBarrier) compared with standard medical therapy for the management of obese subjects with type 2 diabetes mellitus. BMJ open 2017;7(11):e018598. (In eng). DOI: 10.1136/bmjopen-2017-018598.

58. Koehestanie P, de Jonge C, Berends FJ, Janssen IM, Bouvy ND, Greve JW. The effect of the endoscopic duodenal-jejunal bypass liner on obesity and type 2 diabetes mellitus, a multicenter randomized controlled trial. Ann Surg 2014;260(6):984-92. (In eng). DOI: 10.1097/sla.0000000000000794.

59. Caiazzo R, Branche J, Raverdy V, Czernichow S, Carette C, Robert M, et al. Efficacy and Safety of the Duodeno-Jejunal Bypass Liner in Patients With Metabolic Syndrome: A Multicenter Randomized Controlled Trial (ENDOMETAB). Ann Surg 2020;272(5):696-702. (In eng). DOI: 10.1097/sla.0000000000004339.

60. Ruban A, Miras AD, Glaysher MA, Goldstone AP, Prechtl CG, Johnson N, et al. Duodenal-Jejunal Bypass Liner for the management of Type 2 Diabetes Mellitus and Obesity: A Multicenter Randomized Controlled Trial. Ann Surg 2022;275(3):440-447. (In eng). DOI: 10.1097/sla.0000000000004980.

61. Rodriguez L, Reyes E, Fagalde P, Oltra MS, Saba J, Aylwin CG, et al. Pilot clinical study of an endoscopic, removable duodenal-jejunal bypass liner for the treatment of type 2 diabetes. Diabetes Technol Ther 2009;11(11):725-32. (In eng). DOI: 10.1089/dia.2009.0063.

62. Petry TZ, Fabbrini E, Otoch JP, Carmona MA, Caravatto PP, Salles JE, et al. Effect of Duodenal-Jejunal Bypass Surgery on Glycemic Control in Type 2 Diabetes: A Randomized Controlled Trial. Obesity (Silver Spring) 2015;23(10):1973-9. (In eng). DOI: 10.1002/oby.21190.

63. Abu Dayyeh BK, Bazerbachi F, Vargas EJ, Sharaiha RZ, Thompson CC, Thaemert BC, et al. Endoscopic sleeve gastroplasty for treatment of class 1 and 2 obesity (MERIT): a prospective, multicentre, randomised trial. Lancet 2022;400(10350):441-451. (In eng). DOI: 10.1016/s0140-6736(22)01280-6.

64. Coffin B, Maunoury V, Pattou F, Hébuterne X, Schneider S, Coupaye M, et al. Impact of Intragastric Balloon Before Laparoscopic Gastric Bypass on Patients with Super Obesity: a Randomized Multicenter Study. Obes Surg 2017;27(4):902-909. (In eng). DOI: 10.1007/s11695-016-2383-x.

65. Genco A, Maselli R, Frangella F, Cipriano M, Paone E, Meuti V, et al. Effect of consecutive intragastric balloon (BIB®) plus diet versus single BIB® plus diet on eating disorders not otherwise specified (EDNOS) in obese patients. Obes Surg 2013;23(12):2075-9. (In eng). DOI: 10.1007/s11695-013-1028-6.

66. Dargent J, Mion F, Costil V, Ecochard R, Pontette F, Mion V, et al. Multicenter Randomized Study of Obesity Treatment with Minimally Invasive Injection of Hyaluronic Acid Versus and Combined with Intragastric Balloon. Obes Surg 2015;25(10):1842-7. (In eng). DOI: 10.1007/s11695-015-1648-0.

67. Courcoulas A, Abu Dayyeh BK, Eaton L, Robinson J, Woodman G, Fusco M, et al. Intragastric balloon as an adjunct to lifestyle intervention: a randomized controlled trial. Int J Obes (Lond) 2017;41(3):427-433. (In eng). DOI: 10.1038/ijo.2016.229.

68. Fuller NR, Pearson S, Lau NS, Wlodarczyk J, Halstead MB, Tee HP, et al. An intragastric balloon in the treatment of obese individuals with metabolic syndrome: a randomized controlled study. Obesity (Silver Spring) 2013;21(8):1561-70. (In eng). DOI: 10.1002/oby.20414.

69. Ponce J, Quebbemann BB, Patterson EJ. Prospective, randomized, multicenter study evaluating safety and efficacy of intragastric dual-balloon in obesity. Surg Obes Relat Dis 2013;9(2):290-5. (In eng). DOI: 10.1016/j.soard.2012.07.007.

70. Abu Dayyeh BK, Maselli DB, Rapaka B, Lavin T, Noar M, Hussan H, et al. Adjustable intragastric balloon for treatment of obesity: a multicentre, open-label, randomised clinical trial. Lancet 2021;398(10315):1965-1973. (In eng). DOI: 10.1016/s0140-6736(21)02394-1.

71. Lee YM, Low HC, Lim LG, Dan YY, Aung MO, Cheng CL, et al. Intragastric balloon significantly improves nonalcoholic fatty liver disease activity score in obese patients with nonalcoholic steatohepatitis: a pilot study. Gastrointest Endosc 2012;76(4):756-60. (In eng). DOI: 10.1016/j.gie.2012.05.023.

72. Sullivan S, Swain J, Woodman G, Edmundowicz S, Hassanein T, Shayani V, et al. Randomized sham-controlled trial of the 6-month swallowable gas-filled intragastric balloon system for weight loss. Surg Obes Relat Dis 2018;14(12):1876-1889. (In eng). DOI: 10.1016/j.soard.2018.09.486.

73. Miller K, Turró R, Greve JW, Bakker CM, Buchwald JN, Espinós JC. MILEPOST Multicenter Randomized Controlled Trial: 12-Month Weight Loss and Satiety Outcomes After pose (SM) vs. Medical Therapy. Obes Surg 2017;27(2):310-322. (In eng). DOI: 10.1007/s11695-016-2295-9.

74. Sullivan S, Swain JM, Woodman G, Antonetti M, De La Cruz-Muñoz N, Jonnalagadda SS, et al. Randomized sham-controlled trial evaluating efficacy and safety of endoscopic gastric plication for primary obesity: The ESSENTIAL trial. Obesity (Silver Spring) 2017;25(2):294-301. (In eng). DOI: 10.1002/oby.21702.

75. Feigel-Guiller B, Drui D, Dimet J, Zair Y, Le Bras M, Fuertes-Zamorano N, et al. Laparoscopic Gastric Banding in Obese Patients with Sleep Apnea: A 3-Year Controlled Study and Follow-up After 10 Years. Obes Surg 2015;25(10):1886-92. (In eng). DOI: 10.1007/s11695-015-1627-5.

76. Simonson DC, Vernon A, Foster K, Halperin F, Patti ME, Goldfine AB. Adjustable gastric band surgery or medical management in patients with type 2 diabetes and obesity: three-year results of a randomized trial. Surg Obes Relat Dis 2019;15(12):2052-2059. (In eng). DOI: 10.1016/j.soard.2019.03.038.

77. O'Brien PE, Brennan L, Laurie C, Brown W. Intensive medical weight loss or laparoscopic adjustable gastric banding in the treatment of mild to moderate obesity: long-term follow-up of a prospective randomised trial. Obes Surg 2013;23(9):1345-53. (In eng). DOI: 10.1007/s11695-013-0990-3.

78. Morino M, Toppino M, Bonnet G, del Genio G. Laparoscopic adjustable silicone gastric banding versus vertical banded gastroplasty in morbidly obese patients: a prospective randomized controlled clinical trial. Ann Surg 2003;238(6):835-41; discussion 841-2. (In eng). DOI: 10.1097/01.sla.0000098627.18574.72.

79. Dowsey MM, Brown WA, Cochrane A, Burton PR, Liew D, Choong PF. Effect of Bariatric Surgery on Risk of Complications After Total Knee Arthroplasty: A Randomized Clinical Trial. JAMA Netw Open 2022;5(4):e226722. (In eng). DOI: 10.1001/jamanetworkopen.2022.6722.

80. Dixon JB, O'Brien PE, Playfair J, Chapman L, Schachter LM, Skinner S, et al. Adjustable gastric banding and conventional therapy for type 2 diabetes: a randomized controlled trial. Jama 2008;299(3):316-23. (In eng). DOI: 10.1001/jama.299.3.316.

81. Dixon JB, Schachter LM, O'Brien PE, Jones K, Grima M, Lambert G, et al. Surgical vs conventional therapy for weight loss treatment of obstructive sleep apnea: a randomized controlled trial. Jama 2012;308(11):1142-9. (In eng). DOI: 10.1001/2012.jama.11580.

82. Xiang AH, Trigo E, Martinez M, Katkhouda N, Beale E, Wang X, et al. Impact of Gastric Banding Versus Metformin on β-Cell Function in Adults With Impaired Glucose Tolerance or Mild Type 2 Diabetes. Diabetes Care 2018;41(12):2544-2551. (In eng). DOI: 10.2337/dc18-1662.

83. Nilsell K, Thörne A, Sjöstedt S, Apelman J, Pettersson N. Prospective randomised comparison of adjustable gastric banding and vertical banded gastroplasty for morbid obesity. Eur J Surg 2001;167(7):504-9. (In eng). DOI: 10.1080/110241501316914876.

84. Scozzari G, Farinella E, Bonnet G, Toppino M, Morino M. Laparoscopic adjustable silicone gastric banding vs laparoscopic vertical banded gastroplasty in morbidly obese patients: long-term results of a prospective randomized controlled clinical trial. Obes Surg 2009;19(8):1108-15. (In eng). DOI: 10.1007/s11695-009-9871-1.

85. Lundell L, Ruth M, Olbe L. Vertical banded gastroplasty or gastric banding for morbid obesity: effects on gastro-oesophageal reflux. Eur J Surg 1997;163(7):525-31. (In eng).

86. Darabi S, Talebpour M, Zeinoddini A, Heidari R. Laparoscopic gastric plication versus mini-gastric bypass surgery in the treatment of morbid obesity: a randomized clinical trial. Surg Obes Relat Dis 2013;9(6):914-9. (In eng). DOI: 10.1016/j.soard.2013.07.012.

87. Musella M, Vitiello A, Berardi G, Velotti N, Pesce M, Sarnelli G. Evaluation of reflux following sleeve gastrectomy and one anastomosis gastric bypass: 1-year results from a randomized open-label controlled trial. Surg Endosc 2021;35(12):6777-6785. (In eng). DOI: 10.1007/s00464-020-08182-3.

88. Roushdy A, Abdel-Razik MA, Emile SH, Farid M, Elbanna HG, Khafagy W, et al. Fasting Ghrelin and Postprandial GLP-1 Levels in Patients With Morbid Obesity and Medical Comorbidities After Sleeve Gastrectomy and One-anastomosis Gastric Bypass: A Randomized Clinical Trial. Surg Laparosc Endosc Percutan Tech 2020;31(1):28-35. (In eng). DOI: 10.1097/sle.0000000000000844.

89. Hany M, Zidan A, Aboelsoud MR, Torensma B. Laparoscopic sleeve gastrectomy vs one-anastomosis gastric bypass 5-year follow-up: a single-blinded randomized controlled trial. J Gastrointest Surg 2024;28(5):621-633. (In eng). DOI: 10.1016/j.gassur.2024.01.038.

90. Jain M, Tantia O, Goyal G, Chaudhuri T, Khanna S, Poddar A, et al. LSG vs MGB-OAGB: 5-Year Follow-up Data and Comparative Outcome of the Two Procedures over Long Term-Results of a Randomised Control Trial. Obes Surg 2021;31(3):1223-1232. (In eng). DOI: 10.1007/s11695-020-05119-6.

91. Lee WJ, Chong K, Lin YH, Wei JH, Chen SC. Laparoscopic sleeve gastrectomy versus single anastomosis (mini-) gastric bypass for the treatment of type 2 diabetes mellitus: 5-year results of a randomized trial and study of incretin effect. Obes Surg 2014;24(9):1552-62. (In eng). DOI: 10.1007/s11695-014-1344-5.

92. Hedberg S, Thorell A, Österberg J, Peltonen M, Andersson E, Näslund E, et al. Comparison of Sleeve Gastrectomy vs Roux-en-Y Gastric Bypass: A Randomized Clinical Trial. JAMA Netw Open 2024;7(1):e2353141. (In eng). DOI: 10.1001/jamanetworkopen.2023.53141.

93. Skroubis G, Kouri N, Mead N, Kalfarentzos F. Long-term results of a prospective comparison of Roux-en-Y gastric bypass versus a variant of biliopancreatic diversion in a non-superobese population (BMI 35-50 kg/m(2)). Obes Surg 2014;24(2):197-204. (In eng). DOI: 10.1007/s11695-013-1081-1.

94. Mingrone G, Panunzi S, De Gaetano A, Guidone C, Iaconelli A, Capristo E, et al. Metabolic surgery versus conventional medical therapy in patients with type 2 diabetes: 10-year follow-up of an open-label, single-centre, randomised controlled trial. Lancet 2021;397(10271):293-304. (In eng). DOI: 10.1016/s0140-6736(20)32649-0.

95. Salte OBK, Olbers T, Risstad H, Fagerland MW, Søvik TT, Blom-Høgestøl IK, et al. Ten-Year Outcomes Following Roux-en-Y Gastric Bypass vs Duodenal Switch for High Body Mass Index: A Randomized Clinical Trial. JAMA Netw Open 2024;7(6):e2414340. (In eng). DOI: 10.1001/jamanetworkopen.2024.14340.

96. Hall JC, Watts JM, O'Brien PE, Dunstan RE, Walsh JF, Slavotinek AH, et al. Gastric surgery for morbid obesity. The Adelaide Study. Ann Surg 1990;211(4):419-27. (In eng). DOI: 10.1097/00000658-199004000-00007.

97. Casajoana A, Guerrero-Pérez F, García Ruiz de Gordejuela A, Admella V, Sorribas M, Vidal-Alabró A, et al. Role of Gastrointestinal Hormones as a Predictive Factor for Long-Term Diabetes Remission: Randomized Trial Comparing Metabolic Gastric Bypass, Sleeve Gastrectomy, and Greater Curvature Plication. Obes Surg 2021;31(4):1733-1744. (In eng). DOI: 10.1007/s11695-020-05192-x.

98. Nguyen NT, Kim E, Vu S, Phelan M. Ten-year Outcomes of a Prospective Randomized Trial of Laparoscopic Gastric Bypass Versus Laparoscopic Gastric Banding. Ann Surg 2018;268(1):106-113. (In eng). DOI: 10.1097/sla.0000000000002348.

99. Courcoulas AP, Gallagher JW, Neiberg RH, Eagleton EB, DeLany JP, Lang W, et al. Bariatric Surgery vs Lifestyle Intervention for Diabetes Treatment: 5-Year Outcomes From a Randomized Trial. J Clin Endocrinol Metab 2020;105(3):866-76. (In eng). DOI: 10.1210/clinem/dgaa006.

100. Cohen RV, Pereira TV, Aboud CM, Petry TBZ, Lopes Correa JL, Schiavon CA, et al. Effect of Gastric Bypass vs Best Medical Treatment on Early-Stage Chronic Kidney Disease in Patients With Type 2 Diabetes and Obesity: A Randomized Clinical Trial. JAMA Surg 2020;155(8):e200420. (In eng). DOI: 10.1001/jamasurg.2020.0420.

101. Cheng A, Yeoh E, Moh A, Low S, Tan CH, Lam B, et al. Roux-en-Y gastric bypass versus best medical treatment for type 2 diabetes mellitus in adults with body mass index between 27 and 32 kg/m(2): A 5-year randomized controlled trial. Diabetes research and clinical practice 2022;188:109900. (In eng). DOI: 10.1016/j.diabres.2022.109900.

102. Schauer PR, Bhatt DL, Kirwan JP, Wolski K, Aminian A, Brethauer SA, et al. Bariatric Surgery versus Intensive Medical Therapy for Diabetes - 5-Year Outcomes. N Engl J Med 2017;376(7):641-651. (In eng). DOI: 10.1056/NEJMoa1600869.

103. Lee WJ, Huang MT, Yu PJ, Wang W, Chen TC. Laparoscopic vertical banded gastroplasty and laparoscopic gastric bypass: a comparison. Obes Surg 2004;14(5):626-34. (In eng). DOI: 10.1381/096089204323093390.

104. Olbers T, Fagevik-Olsén M, Maleckas A, Lönroth H. Randomized clinical trial of laparoscopic Roux-en-Y gastric bypass versus laparoscopic vertical banded gastroplasty for obesity. Br J Surg 2005;92(5):557-62. (In eng). DOI: 10.1002/bjs.4974.

105. Werling M, Fändriks L, Björklund P, Maleckas A, Brandberg J, Lönroth H, et al. Long-term results of a randomized clinical trial comparing Roux-en-Y gastric bypass with vertical banded gastroplasty. Br J Surg 2013;100(2):222-30. (In eng). DOI: 10.1002/bjs.8975.

106. MacLean LD, Rhode BM, Sampalis J, Forse RA. Results of the surgical treatment of obesity. Am J Surg 1993;165(1):155-60; discussion 160-2. (In eng). DOI: 10.1016/s0002-9610(05)80420-9.

107. Cummings DE, Arterburn DE, Westbrook EO, Kuzma JN, Stewart SD, Chan CP, et al. Gastric bypass surgery vs intensive lifestyle and medical intervention for type 2 diabetes: the CROSSROADS randomised controlled trial. Diabetologia 2016;59(5):945-53. (In eng). DOI: 10.1007/s00125-016-3903-x.

108. Liang Z, Wu Q, Chen B, Yu P, Zhao H, Ouyang X. Effect of laparoscopic Roux-en-Y gastric bypass surgery on type 2 diabetes mellitus with hypertension: a randomized controlled trial. Diabetes research and clinical practice 2013;101(1):50-6. (In eng). DOI: 10.1016/j.diabres.2013.04.005.

109. Schiavon CA, Bhatt DL, Ikeoka D, Santucci EV, Santos RN, Damiani LP, et al. Three-Year Outcomes of Bariatric Surgery in Patients With Obesity and Hypertension : A Randomized Clinical Trial. Ann Intern Med 2020;173(9):685-693. (In eng). DOI: 10.7326/m19-3781.

110. Ikramuddin S, Korner J, Lee WJ, Thomas AJ, Connett JE, Bantle JP, et al. Lifestyle Intervention and Medical Management With vs Without Roux-en-Y Gastric Bypass and Control of Hemoglobin A1c, LDL Cholesterol, and Systolic Blood Pressure at 5 Years in the Diabetes Surgery Study. Jama 2018;319(3):266-278. (In eng). DOI: 10.1001/jama.2017.20813.

111. Delko T, Kraljević M, Lazaridis, II, Köstler T, Jomard A, Taheri A, et al. Laparoscopic Roux-Y-gastric bypass versus laparoscopic one-anastomosis gastric bypass for obesity: clinical & metabolic results of a prospective randomized controlled trial. Surg Endosc 2024;38(7):3875-3886. (In eng). DOI: 10.1007/s00464-024-10907-7.

112. Eskandaros MS, Abbass A, Zaid MH, Darwish AA. Laparoscopic One Anastomosis Gastric Bypass Versus Laparoscopic Roux-en-Y Gastric Bypass Effects on Pre-existing Mild-to-Moderate Gastroesophageal Reflux Disease in Patients with Obesity: a Randomized Controlled Study. Obes Surg 2021;31(11):4673-4681. (In eng). DOI: 10.1007/s11695-021-05667-5.

113. Karagul S, Senol S, Karakose O, Uzunoglu K, Kayaalp C. One Anastomosis Gastric Bypass versus Roux-en-Y Gastric Bypass: A Randomized Prospective Trial. Medicina (Kaunas) 2024;60(2) (In eng). DOI: 10.3390/medicina60020256.

114. Singh B, Saikaustubh Y, Singla V, Kumar A, Ahuja V, Gupta Y, et al. One Anastomosis Gastric Bypass (OAGB) vs Roux en Y Gastric Bypass (RYGB) for Remission of T2DM in Patients with Morbid Obesity: a Randomized Controlled Trial. Obes Surg 2023;33(4):1218-1227. (In eng). DOI: 10.1007/s11695-023-06515-4.

115. Level L, Rojas A, Piñango S, Avariano Y. One anastomosis gastric bypass vs. Roux-en-Y gastric bypass: a 5-year follow-up prospective randomized trial. Langenbecks Arch Surg 2021;406(1):171-179. (In eng). DOI: 10.1007/s00423-020-01949-1.

116. Robert M, Espalieu P, Pelascini E, Caiazzo R, Sterkers A, Khamphommala L, et al. Efficacy and safety of one anastomosis gastric bypass versus Roux-en-Y gastric bypass for obesity (YOMEGA): a multicentre, randomised, open-label, non-inferiority trial. Lancet 2019;393(10178):1299-1309. (In eng). DOI: 10.1016/s0140-6736(19)30475-1.

117. Biter LU, van Buuren MMA, Mannaerts GHH, Apers JA, Dunkelgrün M, Vijgen G. Quality of Life 1 Year After Laparoscopic Sleeve Gastrectomy Versus Laparoscopic Roux-en-Y Gastric Bypass: a Randomized Controlled Trial Focusing on Gastroesophageal Reflux Disease. Obes Surg 2017;27(10):2557-2565. (In eng). DOI: 10.1007/s11695-017-2688-4.

118. Karamanakos SN, Vagenas K, Kalfarentzos F, Alexandrides TK. Weight loss, appetite suppression, and changes in fasting and postprandial ghrelin and peptide-YY levels after Roux-en-Y gastric bypass and sleeve gastrectomy: a prospective, double blind study. Ann Surg 2008;247(3):401-7. (In eng). DOI: 10.1097/SLA.0b013e318156f012.

119. Keidar A, Hershkop KJ, Marko L, Schweiger C, Hecht L, Bartov N, et al. Roux-en-Y gastric bypass vs sleeve gastrectomy for obese patients with type 2 diabetes: a randomised trial. Diabetologia 2013;56(9):1914-8. (In eng). DOI: 10.1007/s00125-013-2965-2.

120. Paluszkiewicz R, Kalinowski P, Wróblewski T, Bartoszewicz Z, Białobrzeska-Paluszkiewicz J, Ziarkiewicz-Wróblewska B, et al. Prospective randomized clinical trial of laparoscopic sleeve gastrectomy versus open Roux-en-Y gastric bypass for the management of patients with morbid obesity. Wideochir Inne Tech Maloinwazyjne 2012;7(4):225-32. (In eng). DOI: 10.5114/wiitm.2012.32384.

121. Ramón JM, Salvans S, Crous X, Puig S, Goday A, Benaiges D, et al. Effect of Roux-en-Y gastric bypass vs sleeve gastrectomy on glucose and gut hormones: a prospective randomised trial. J Gastrointest Surg 2012;16(6):1116-22. (In eng). DOI: 10.1007/s11605-012-1855-0.

122. Verrastro O, Panunzi S, Castagneto-Gissey L, De Gaetano A, Lembo E, Capristo E, et al. Bariatric-metabolic surgery versus lifestyle intervention plus best medical care in non-alcoholic steatohepatitis (BRAVES): a multicentre, open-label, randomised trial. Lancet 2023;401(10390):1786-1797. (In eng). DOI: 10.1016/s0140-6736(23)00634-7.

123. Tang Q, Sun Z, Zhang N, Xu G, Song P, Xu L, et al. Cost-Effectiveness of Bariatric Surgery for Type 2 Diabetes Mellitus: A Randomized Controlled Trial in China. Medicine (Baltimore) 2016;95(20):e3522. (In eng). DOI: 10.1097/md.0000000000003522.

124. Wallenius V, Alaraj A, Björnfot N, Orrenius B, Kylebäck A, Björklund P, et al. Sleeve gastrectomy and Roux-en-Y gastric bypass in the treatment of type 2 diabetes. Two-year results from a Swedish multicenter randomized controlled trial. Surg Obes Relat Dis 2020;16(8):1035-1044. (In eng). DOI: 10.1016/j.soard.2020.04.033.

125. Pajecki D, Dos Anjos Pinheiro MC, Dantas ACB, Corsi GC, Dias MCG, Santo MA. Sleeve Gastrectomy Versus Roux-en-Y Gastric Bypass for Treating Obesity in Patients > 65 Years Old: 3-Year Outcomes of a Randomized Trial. J Gastrointest Surg 2023;27(4):780-782. (In eng). DOI: 10.1007/s11605-023-05608-w.

126. Catheline JM, Fysekidis M, Bendacha Y, Portal JJ, Huten N, Chouillard E, et al. Prospective, multicentric, comparative study between sleeve gastrectomy and Roux-en-Y gastric bypass, 277 patients, 3 years follow-up. J Visc Surg 2019;156(6):497-506. (In eng). DOI: 10.1016/j.jviscsurg.2019.04.013.

127. Kehagias I, Karamanakos SN, Argentou M, Kalfarentzos F. Randomized clinical trial of laparoscopic Roux-en-Y gastric bypass versus laparoscopic sleeve gastrectomy for the management of patients with BMI < 50 kg/m2. Obes Surg 2011;21(11):1650-6. (In eng). DOI: 10.1007/s11695-011-0479-x.

128. Svanevik M, Lorentzen J, Borgeraas H, Sandbu R, Seip B, Medhus AW, et al. Patient-reported outcomes, weight loss, and remission of type 2 diabetes 3 years after gastric bypass and sleeve gastrectomy (Oseberg); a single-centre, randomised controlled trial. Lancet Diabetes Endocrinol 2023;11(8):555-566. (In eng). DOI: 10.1016/s2213-8587(23)00127-4.

129. Yang J, Wang C, Cao G, Yang W, Yu S, Zhai H, et al. Long-term effects of laparoscopic sleeve gastrectomy versus roux-en-Y gastric bypass for the treatment of Chinese type 2 diabetes mellitus patients with body mass index 28-35 kg/m(2). BMC Surg 2015;15:88. (In eng). DOI: 10.1186/s12893-015-0074-5.

130. Ignat M, Vix M, Imad I, D'Urso A, Perretta S, Marescaux J, et al. Randomized trial of Roux-en-Y gastric bypass versus sleeve gastrectomy in achieving excess weight loss. Br J Surg 2017;104(3):248-256. (In eng). DOI: 10.1002/bjs.10400.

131. Peterli R, Wölnerhanssen BK, Peters T, Vetter D, Kröll D, Borbély Y, et al. Effect of Laparoscopic Sleeve Gastrectomy vs Laparoscopic Roux-en-Y Gastric Bypass on Weight Loss in Patients With Morbid Obesity: The SM-BOSS Randomized Clinical Trial. Jama 2018;319(3):255-265. (In eng). DOI: 10.1001/jama.2017.20897.

132. Zhang Y, Zhao H, Cao Z, Sun X, Zhang C, Cai W, et al. A randomized clinical trial of laparoscopic Roux-en-Y gastric bypass and sleeve gastrectomy for the treatment of morbid obesity in China: a 5-year outcome. Obes Surg 2014;24(10):1617-24. (In eng). DOI: 10.1007/s11695-014-1258-2.

133. Pullman JS, Plank LD, Nisbet S, Murphy R, Booth MWC. Seven-Year Results of a Randomized Trial Comparing Banded Roux-en-Y Gastric Bypass to Sleeve Gastrectomy for Type 2 Diabetes and Weight Loss. Obes Surg 2023;33(7):1989-1996. (In eng). DOI: 10.1007/s11695-023-06635-x.

134. Salminen P, Grönroos S, Helmiö M, Hurme S, Juuti A, Juusela R, et al. Effect of Laparoscopic Sleeve Gastrectomy vs Roux-en-Y Gastric Bypass on Weight Loss, Comorbidities, and Reflux at 10 Years in Adult Patients With Obesity: The SLEEVEPASS Randomized Clinical Trial. JAMA Surg 2022;157(8):656-666. (In eng). DOI: 10.1001/jamasurg.2022.2229.

135. Axer S, Al-Tai S, Ihle C, Alwan M, Hoffmann L. Perioperative Safety and 1-Year Outcomes of Single-Anastomosis Duodeno-Ileal Bypass (SADI) vs. Biliopancreatic Diversion with Duodenal Switch (BPD/DS): A Randomized Clinical Trial. Obes Surg 2024;34(9):3382-3389. (In eng). DOI: 10.1007/s11695-024-07421-z.

136. Talebpour M, Sadid D, Talebpour A, Sharifi A, Davari FV. Comparison of Short-Term Effectiveness and Postoperative Complications: Laparoscopic Gastric Plication vs Laparoscopic Sleeve Gastrectomy. Obes Surg 2018;28(4):996-1001. (In eng). DOI: 10.1007/s11695-017-2951-8.

137. Grubnik VV, Ospanov OB, Namaeva KA, Medvedev OV, Kresyun MS. Randomized controlled trial comparing laparoscopic greater curvature plication versus laparoscopic sleeve gastrectomy. Surg Endosc 2016;30(6):2186-91. (In eng). DOI: 10.1007/s00464-015-4373-9.

138. Spaggiari M, Di Cocco P, Tulla K, Kaylan KB, Masrur MA, Hassan C, et al. Simultaneous robotic kidney transplantation and bariatric surgery for morbidly obese patients with end-stage renal failure. Am J Transplant 2021;21(4):1525-1534. (In eng). DOI: 10.1111/ajt.16322.
